# Non-*β*-Lactam Allosteric Inhibitors Target Methicillin-Resistant *Staphylococcus aureus*: An *In Silico* Drug Discovery Study

**DOI:** 10.3390/antibiotics10080934

**Published:** 2021-08-01

**Authors:** Mahmoud A. A. Ibrahim, Khlood A. A. Abdeljawaad, Alaa H. M. Abdelrahman, Othman R. Alzahrani, Fahad M. Alshabrmi, Esraa Khalaf, Mahmoud F. Moustafa, Faris Alrumaihi, Khaled S. Allemailem, Mahmoud E. S. Soliman, Paul W. Paré, Mohamed-Elamir F. Hegazy, Mohamed A. M. Atia

**Affiliations:** 1 Computational Chemistry Laboratory, Chemistry Department, Faculty of Science, Minia University, Minia 61519, Egypt; kh.abdeljawaad@compchem.net (K.A.A.A.); a.abdelrahman@compchem.net (A.H.M.A.); 2 Department of Biology, Faculty of Sciences, University of Tabuk, Tabuk 71491, Saudi Arabia; O-alzahrani@ut.edu.sa; 3 Department of Medical Laboratories, College of Applied Medical Sciences, Qassim University, Buraydah 51452, Saudi Arabia; fshbrmy@qu.edu.sa (F.M.A.); f_alrumaihi@qu.edu.sa (F.A.); k.allemailem@qu.edu.sa (K.S.A.); 4 Department of Bacteriology, Mycology and Immunology, Faculty of Veterinary Medicine, Beni-Suef University, Beni-Suef 62511, Egypt; dresraa.fathy@yahoo.com; 5 Department of Biology, College of Science, King Khalid University, Abha 9004, Saudi Arabia; hamdony@yahoo.com or; 6 Department of Botany & Microbiology, Faculty of Science, South Valley University, Qena 83523, Egypt; 7 Molecular Bio-Computation and Drug Design Lab, School of Health Sciences, University of KwaZulu-Natal, Westville, Durban 4000, South Africa; soliman@ukzn.ac.za; 8 Department of Chemistry and Biochemistry, Texas Tech University, Lubbock, TX 79409, USA; paul.pare@ttu.edu; 9 Chemistry of Medicinal Plants Department, National Research Centre, Giza 12622, Egypt; 10 Department of Pharmaceutical Biology, Institute of Pharmaceutical and Biomedical Sciences, Johannes Gutenberg University, 55128 Mainz, Germany; 11 Molecular Genetics and Genome Mapping Laboratory, Genome Mapping Department, Agricultural Genetic Engineering Research Institute (AGERI), Agricultural Research Center (ARC), Giza 12619, Egypt

**Keywords:** *Staphylococcus aureus*, PBP2a, *MecA*, pharmacophore, molecular docking, molecular dynamics simulations

## Abstract

Penicillin-binding proteins (PBPs) catalyze the final stages for peptidoglycan cell-wall bio-synthesis. Mutations in the PBP2a subunit can attenuate *β*-lactam antibiotic activity, resulting in unimpeded cell-wall formation and methicillin-resistant *Staphylococcus aureus* (MRSA). A double mutation in PBP2a (i.e., N146K and E150K) is resistant to *β*-lactam inhibitors; however, (*E*)-3-(2-(4-cyanostyryl)-4-oxoquinazolin-3(4*H*)-yl) benzoic acid (QNZ), a heterocyclic antibiotic devoid of a *β*-lactam ring, interacts non-covalently with PBP2a allosteric site and inhibits PBP enzymatic activity. In the search for novel inhibitors that target this PBP2a allosteric site in acidic medium, an *in silico* screening was performed. Chemical databases including eMolecules, ChEMBL, and ChEBI were virtually screened for candidate inhibitors with a physicochemical similarity to QNZ. PBP2a binding affinities from the screening were calculated based on molecular docking with co-crystallized ligand QNZ serving as a reference. Molecular minimization calculations were performed for inhibitors with docking scores lower than QNZ (calc. −8.3 kcal/mol) followed by combined MD simulations and MM-GBSA binding energy calculations. Compounds eMol26313223 and eMol26314565 exhibited promising inhibitor activities based on binding affinities (Δ*G*_binding_) that were twice that of QNZ (−38.5, −34.5, and −15.4 kcal/mol, respectively). Structural and energetic analyses over a 50 ns MD simulation revealed high stability for the inhibitors when complexed with the double mutated PBP2a. The pharmacokinetic properties of the two inhibitors were predicted using an *in silico* ADMET analysis. Calculated binding affinities hold promise for eMol26313223 and eMol26314565 as allosteric inhibitors of PBP2a in acidic medium and establish that further *in vitro* and *in vivo* inhibition experimentation is warranted.

## 1. Introduction

With multidrug-resistant bacteria on the rise and bacterial infections imposing a major threat to public health, there is an imminent need for the development of new antibiotics [1,2]. For example, methicillin-resistant *Staphylococcus aureus* (MRSA) has acquired drug resistance to many *β*-lactam antibiotics. In the 1960s, MRSA was identified in European hospitals as a causative agent in skin and respiratory infections [3,4,5,6,7]; since then, MRSA has become a leading cause of death in the United States, accounting for more than 19,000 deaths annually [8]. While anti-MRSA drugs have been developed, including methicillin, penicillin, cephalosporins, and carbapenems [9,10,11], new MRSA-resistant strains continue to appear, threatening the current repository of antibiotics [12,13,14]. At the heart of antibiotic resistance is a *mecA* gene that encodes for a penicillin-binding protein (PBP). PBPs catalyze the final stages of peptidoglycan cell-wall synthesis with mutations in the PBP2a subunit attenuating *β*-lactam inhibitor binding, resulting in unimpeded cell-wall formation [15,16]. PBP2a inhibition depends on the opening of the active site through a conformational change in the *β*3–*β*4 loops to allow drug entry [17,18]. This conformational change is mediated by a small molecule allosteric regulation 60 Å from the active site [18,19]. Communication between these two sites has been characterized by targeted molecular dynamics (TMD) simulations [17].

Ceftaroline (CFT) is an anti-MRSA inhibitor that interacts covalently with the PBP2a active site and non-covalently with the allosteric site [17,18]. Bacterial resistance to CFT originating in the allosteric site is caused by N146K and E150K mutations in PBP2a [20]. Quinazolinone (QNZ), a heterocyclic antibiotic devoid of a *β*-lactam ring, inhibits PBP activity [21,22,23,24]. QNZ, similar to CFT, binds non-covalently to the allosteric site, triggering an active site opening, allowing a second quinazolinone to bind to the active site that inhibits cell-wall biosynthesis [21]. QNZ has greater PBP2a inhibitor activity than *β*-lactam based drugs [23].

In addition to the developed antibiotic resistance, the pH of medium is a critical factor for the antibacterial activity of drugs [25]. MRSA causes skin infections, where skin pH is normally acidic (a range of pH values of 4–6). Oral administration of antibacterial drugs for treating skin infections is an additional parameter that should be considered in the drug discovery and development process. Considering the pH influence, pharmacophore-based virtual screenings were utilized to identify inhibitors that have similar physicochemical properties to those of QNZ that can effectively bind to the double mutated PBP2a allosteric site in acidic medium. Three large chemical databases—namely, eMolecules, ChEMBL and ChEBI—were screened, and potent inhibitors were filtered based on molecular docking calculations and molecular mechanics (MM) minimizations. Binding energies of the potent inhibitors in complex with PBP2a protein are evaluated over 50 ns molecular dynamics (MD) simulations using a molecular mechanics-generalized born surface area (MM-GBSA) approach. The stabilities of identified inhibitors were then examined based on pharmacokinetic properties.

## 2. Results

### 2.1. Validation of In Silico Protocol

**Figure 1:**
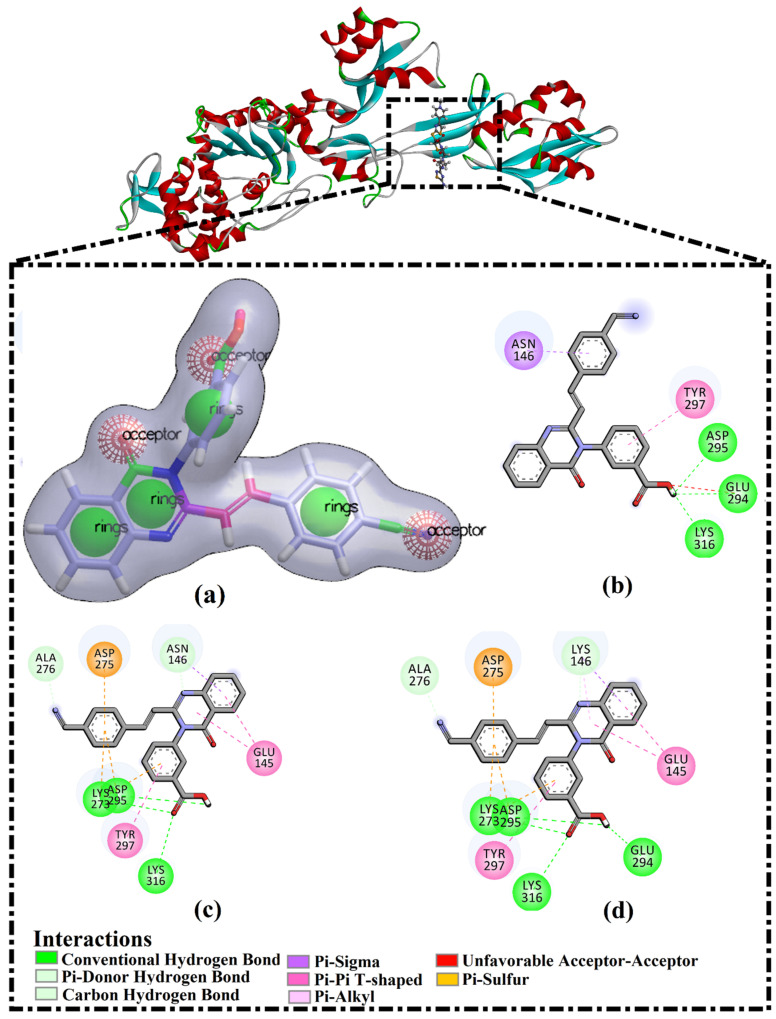
(**a**) 3D pharmacophore model of QNZ, (**b**) 2D representations of the experimental binding mode of QNZ with wild PBP2a allosteric site, and predicted binding modes of QNZ with (**c**) wild and (**d**) double mutated PBP2a allosteric site in acidic medium.

Prior to molecular docking calculations, AutoDock parameters and protocol were assessed according to the available experimental data. The co-crystallized QNZ inhibitor in acidic form was re-docked against a wild and double mutated PBP2a allosteric site, and the anticipated binding modes were compared to an experimental structure (PDB code: 4CJN) (Figure 1). As anticipated, docking poses were in conformity with the crystal structure, exhibiting essential hydrogen modes with LYS316 and ASP295 in the allosteric site (Figure 1). The docking software accurately predicted the correct binding mode for QNZ inside the allosteric site of PBP2a. Moreover, the double N146K and E150K mutants did not affect the binding of QNZ. Based on the anticipated docking scores, QNZ-wild PBP2a and QNZ-mutated PBP2a complexes demonstrated a robust binding affinity with docking scores of −8.0 and −8.3 kcal/mol, respectively. Compared to QNZ, CFT exhibited a lower binding affinity towards wild and mutated PBP2a with docking scores of −3.5 and −3.5 kcal/mol, respectively. Notably, QNZ was found to be more potent than CFT (calculated *k*_i_ = 804.12 and 2.73 mM, respectively) towards the mutated PBP2a. The greatest potency of QNZ against PBP2a could be imputed to its capability of a carboxylic group of QNZ to form two hydrogen bonds with the backbone carbonyl group and ammonium group (NH_3_^+^) of ASP295 and LYS316 with bond lengths of 3.18 and 2.94 Å, respectively (Figure 1).

The influence of pH on the binding mode and affinity of QNZ was inspected by re-docking the unionized and ionized forms of QNZ (i.e., in carboxylic and carboxylate states) against the mutated PBP2a (Figure S1). As shown in Figure S1, the binding modes of the QNZ in the two states were almost identical with an RMSD of 0.12 Å, giving similar docking scores of −8.3 and −8.1 kcal/mol, for unionized and ionized states, respectively.

In contrast, CFT was not able to form hydrogen bonds with allosteric site amino acids, resulting in weak binding with PBP2a (Figure S2). In addition, the 3D and 2D representations of the predicted binding modes of CFT and QNZ inside PBP2a allosteric site were depicted in Figure S3. Inspecting the experimental structure of CFT with wild PBP2a (PDB code: 3ZG0 [18]) revealed that CFT binds in different orientations with A and B chains, demonstrating the instability of CFT inside PBP2a allosteric site. These findings justify the high ligand affinity of QNZ over CFT for PBP2a. Consequently, chemical databases were explored to identify similar physicochemical inhibitors to QNZ as potent allosteric inhibitors using a pharmacophore-based virtual screening. Prior to the virtual screening study, further calculations, including molecular minimization, molecular dynamics simulations, and binding affinity calculations, were needed to confirm the potency of QNZ against both wild and mutated PBP2a.

### 2.2. QNZ Complexed with Wild and Mutated PBP2a

Molecular mechanics (MM) minimization of small molecules in complex with a receptor in an implicit solvent followed by MM-GBSA binding energy calculations can predict binding affinity with a satisfactory correlation with the experimental data [26]. Therefore, in order to confirm the higher binding affinity of QNZ, molecular minimization, molecular dynamics simulations followed by MM-GBSA binding energy calculations were carried out for QNZ with double mutated PBP2a and compared to that of QNZ with wild PBP2a (Table 1). The estimated binding energy based on the minimized structure (i.e., MM-GBSA//MM) of QNZ with mutated PBP2a was similar to that of QNZ with wild PBP2a (Table 1). More exactly, QNZ demonstrated robust MM-GBSA//MM binding energies with values of −30.5 and −31.4 kcal/mol with wild and mutated PBP2a, respectively.

In an attempt to gain further confidence in the results and specifically identify the impact of N146K and E150K mutations on QNZ binding, molecular dynamics simulations were carried out over 50 ns with wild and double mutated PBP2a complexed with QNZ. In addition, the corresponding MM-GBSA//MD binding energies were estimated over the 50 ns MD simulations (Table 1). From these data, it is apparent that the average MM-GBSA binding energies (∆*G*_binding_) for QNZ in complex with wild and mutated PBP2a at −15.3 and −15.4 kcal/mol, respectively, are robust. To probe the nature of QNZ interactions when complexed with PBP2a, decomposition of the MM-GBSA//MD binding energies was performed (Table 1). Binding energy decompositions for QNZ with wild and mutated PBP2a were dominated by *E*_vdw_ interactions with an average value of −25.3 and −24.7 kcal/mol, respectively. Additionally, the *E*_ele_ interaction of QNZ with PBP2a is favorable (calc. −9.4 and −15.7 kcal/mol for QNZ with wild and mutated PBP2a, respectively). All presented results herein demonstrated that N146K and E150K mutations of PBP2a do not have a resistant effect on QNZ.

To evaluate the structural and energetic stabilities for complexed QNZ, post-MD analyses were performed, including root-mean-square deviation (RMSD) and binding energy per frame. The root-mean-square deviations (RMSDs) for the whole complex backbone atoms were inspected to examine the dynamic stability of QNZ in complex with PBP2a (Figure S4). The RMSD plot elucidated that QNZ in complex with wild and mutated PBP2a achieved stability in a short time with RMSD values of about 0.45 nm (Figure S4). Moreover, the binding energy per frame plot (Figure S5) showed that there was overall QNZ stability in complex with wild and mutated PBP2a throughout the MD simulation with average binding energies (Δ*G*_binding_) of −15.3 and −15.4 kcal/mol, respectively. These findings demonstrated the high stability of complexed QNZ throughout the 50 ns MD simulations and that QNZ does not impact the overall topology of the PBP2a allosteric site.

**Figure 2:**
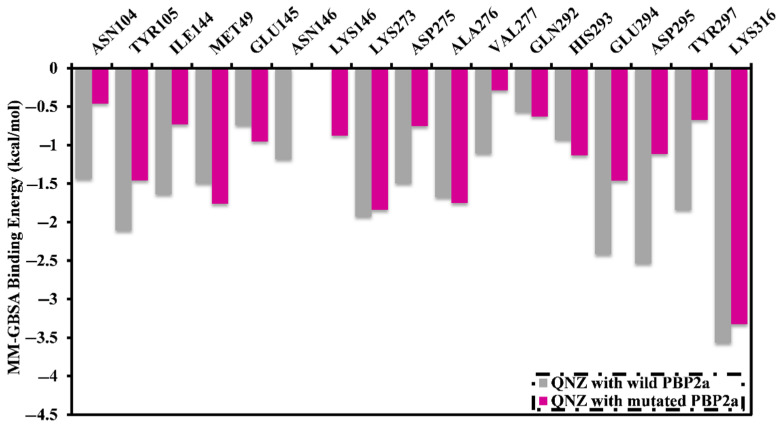
Energy contributions (in kcal/mol) for PBP2a amino acid residues to the total MM-GBSA binding energies of QNZ in complex with wild and mutated PBP2a allosteric site in acidic medium.

Binding energies for QNZ in complex with wild and mutated PBP2a were further decomposed at the pre-residue level, and the amino acid residues with free energy participation <−0.50 kcal/mol were illustrated (Figure 2). PBP2a allosteric site residues LYS273, GLU294, ASP295, and LYS316 share interactions with QNZ (Figure 2). Moreover, there was participation by LYS316 towards the total binding free energy with values of −3.6 and −3.3 kcal/mol for complex QNZ with wild and mutated PBP2a, respectively. This is consistent with X-ray crystal resolved structural analysis of QNZ with PBP2a (PDB code: 4CJN [21]).

### 2.3. Pharmacophore-Based Virtual Screening

QNZ pharmacophore screening identified the chemical features likely involved in allosteric inhibition against the double mutated PBP2a. Common pharmacophoric features involved hydrogen bond donors (HBD), hydrogen bond acceptors (HBA), hydrophobic interactions (H), and aromatic ring systems (RA) (see Figure 1). A similarity screening based on a QNZ 3D pharmacophore model was executed with eMolecules (http://www.emolecules.com), ChEMBL [27], and ChEBI [28] databases, containing more than 25 million molecules, employing ROCS software [29,30]. Hits were ranked based on Tanimoto combo similarity values, considering shape similarity (ShapeTanimoto) and chemical pattern (ColorScore) similarity [31].

### 2.4. Database Filteration

In order to reduce computational costs and run time, the top 5000 pharmacophore screening hits were docked against a mutated PBP2a complex using a conventional docking protocol (see computational methodology section for details) (Table S1). From conventional molecular docking calculations, the top 1000 hits were selected and re-docked with the mutated PBP2a using intermediate molecular docking protocol (see computational methodology section for details) (Table S2). Based on evaluated intermediate molecular docking calculations, the best 250 hits were subjected to expensive molecular docking calculations. The estimated docking scores for the top 250 hits against the mutated PBP2a allosteric site are summarized in Table S3. Thirty-five inhibitors in this group exhibited docking scores equal to or lower than the co-crystalized inhibitor (QNZ = −8.3 kcal/mol). The 2D chemical structures and the estimated conventional, intermediate, and expensive docking scores for those most promising inhibitors are summarized in Table 2.

Inspecting the 2D structures of the potent inhibitors demonstrated that these inhibitors are close analogs of QNZ, and 4(3*H*)-quinazolinone plays a vital role in the high binding affinity of these inhibitors with the mutated PBP2a allosteric site (Table 2). The 2D inhibitor binding interacting with residues in the allosteric site of the mutated PBP2a (Figure S6) again shows hydrogen bonding with LYS*273*, GLU294, ASP295, and LYS316. Hydrophobic, van der Waals, and pi-based interactions were also observed between the investigated compounds and proximal residues in the mutated PBP2a allosteric site (Figure S6).

**Figure 3:**
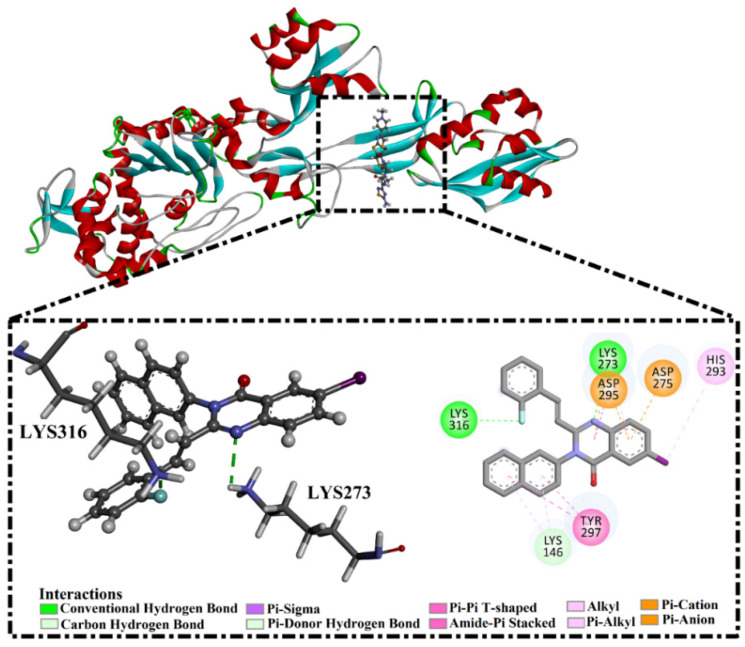
3D and 2D representations of the binding modes of eMol26313223 in a mutated PBP2a allosteric site in acidic medium.

Specifically, eMol26313223 demonstrated outstanding mutated PBP2a binding affinity with a docking score of −10.0 kcal/mol. Structural insights into the binding mode of eMol26313223 towards the mutated PBP2a revealed that fluorine and nitrogen atoms of the iodo-methylquinazolin and fluorobenzene rings form two hydrogen bonds with the ammonium group (NH_3_^+^) of LYS273 and LYS316 with bond lengths of 2.50 and 2.02 Å, respectively (Figure 3).

### 2.5. Inhibitor-PBP2a Complex Minimization

**Figure 4:**
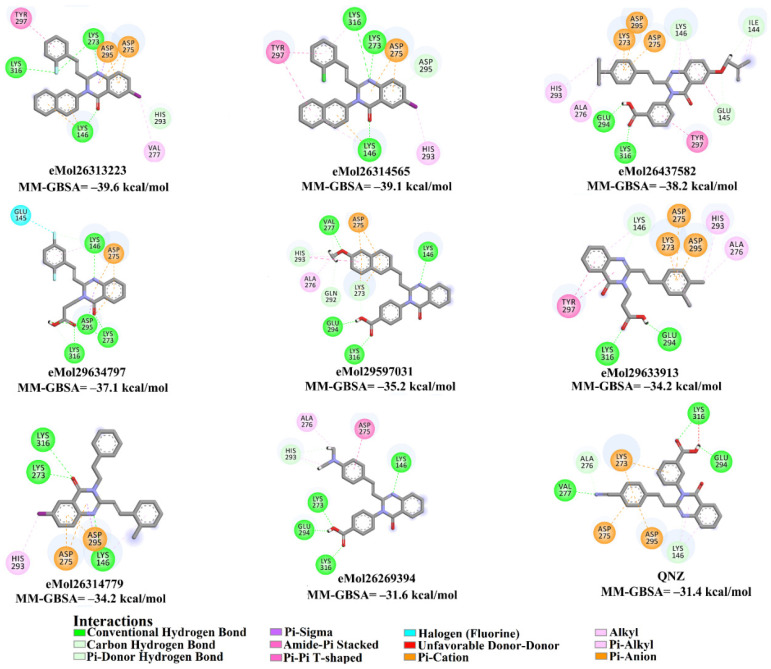
2D representation of AMBER-based minimized structures, as well as estimated MM-GBSA//MM binding energies, of the eight potent molecules and QNZ in complex with the mutated PBP2a allosteric site in acidic medium.

The 35 inhibitors that complexed most strongly with the mutated PBP2a were energetically minimized using an AMBER-based molecular mechanics force field. Based on the minimized complexes, the MM-GBSA binding energies were estimated (Table S4) with eight compounds showing MM-GBSA//MM binding energies lower than QNZ (Δ*G*_binding_ = −31.4 kcal/mol). The 2D and 3D representations of eight molecules complexed with mutated PBP2a are depicted in Figure 4 and Figure S7, respectively. The investigated molecules exhibited similar binding modes, forming hydrogen bonds with the proximal residue LYS316, in addition to other hydrogen bonding inside the mutated PBP2a allosteric site; van der Waals, pi-based, and hydrophobic interactions were also observed (Figure 4 and Table S4). Inspecting the 2D structure of the top eight inhibitors revealed that these inhibitors are 2-styrenyl-4(3*H*)-quinazolinones. Compound eMol26313223 manifested a superior binding affinity towards the mutated PBP2a with an MM-GBSA//MM binding energy of −39.6 kcal/mol with a fluorine atom of fluorobenzene ring exhibiting two hydrogen bonds with the ammonium group (NH_3_^+^) of LYS273 and LYS316 with bond lengths of 2.21 and 2.19 Å, respectively (Figure 4). At the same time, the carbonyl group and nitrogen atom of the iodo-methyl quinazoline ring form two hydrogen bonds with the ammonium group (NH_3_^+^) of LYS146 and LYS273 with bond lengths of 1.89 and 1.94 Å, respectively (Figure 4).

Compound eMol26314565 displayed the second-highest mutated PBP2a allosteric binding affinity with an MM-GBSA//MM binding energy of −39.1 kcal/mol; similarly, a carbonyl group and nitrogen atom of an iodo-methyl quinazoline ring form three hydrogen bonds with the ammonium group (NH_3_^+^) of LYS146, LYS273 and LYS316 with bond lengths of 1.97, 2.03, and 3.08 Å, respectively (Figure 4).

Compound eMol26437582 demonstrated the third-highest binding affinity towards mutated PBP2a with an MM-GBSA//MM binding energy of −38.2 kcal/mol; again, carboxylic group interactions were observed with the carboxylate and ammonium group (NH_3_^+^) of GLU294 and LYS316 with bond lengths of 1.59 and 1.82 Å, respectively (Figure 4). Compared to the three newly identified compounds, QNZ exhibited a moderate MM-GBSA//MM binding energy of −34.4 kcal/mol against the mutated PBP2a allosteric site. Examining the binding mode of QNZ in the mutated PBP2a allosteric site revealed that (i) a nitrogen atom of benzonitrile forms a hydrogen bond with the backbone of NH_2_ of VAL277 with the bond length of 2.26 Å, (ii) the hydroxyl of the carboxylic acid group exhibits a hydrogen bond with the carboxylate of GLU294 with a bond length of 1.57 Å, and (iii) the carbonyl group of carboxylic acid forms two hydrogen bonds with the ammonium group (NH_3_^+^) of LYS273 with bond lengths of 2.60 and 2.72 Å (Figure 4). It is also worth noting that the 4-carbonyl group significantly contributed to the interaction of eMol26313223 and eMol26314565 with the ammonium group (NH_3_^+^) of LYS146. However, such an interaction was not demonstrated by eMol26437582, resulting in a lower binding affinity compared to eMol26313223 and eMol26314565.

### 2.6. Molecular Dynamics (MD) Simulations

**Figure 5:**
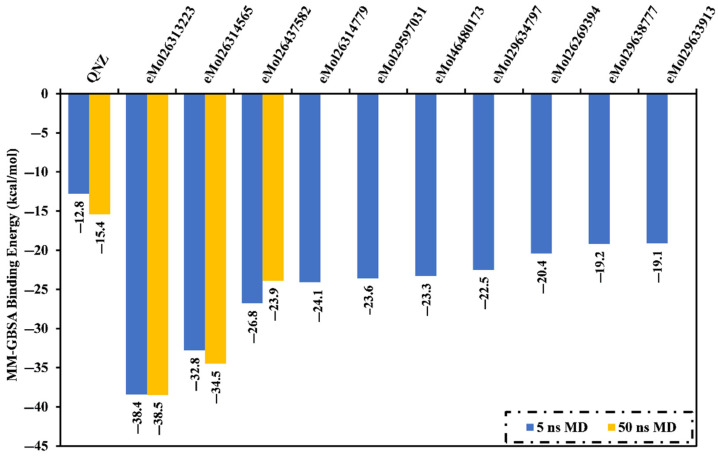
Evaluated MM-GBSA binding energies for the co-crystallized QNZ ligand and the best eight potent inhibitors in complex with mutated PBP2a allosteric site in acidic medium over 5 ns and 50 ns MD simulations.

Molecular dynamics (MD) simulations were used to probe the stability of the inhibitor-receptor complex, conformational flexibilities, structural details, and recognize dependable ligand-receptor binding affinities [32,33]. Eight compounds that complexed with the mutated PBP2a allosteric site were submitted to MD simulations, and corresponding MM-GBSA binding energies were estimated (Figure 5). Interestingly, all investigated inhibitors demonstrated binding affinities higher than QNZ (calc. −12.8 kcal/mol) towards mutated PBP2a (Figure 5). Three inhibitors, compounds eMol26313223, eMol26314565, and eMol26437582, demonstrated promising binding energies for mutated PBP2a with Δ*G*_binding_ <−25.0 kcal/mol, attributed to a capacity to form stable hydrogen bonds with LYS316. Interestingly, predicted docking for eMol26314565 and eMol26437582 lacks hydrogen bonding with LYS316 (Figure S5).

To increase the reliability of the observed findings, MD simulations were extended to 50 ns, and the corresponding binding energies were calculated (Figure 5). Compound eMol26437582 demonstrated an increase in MM-GBSA binding energies compared to 5 ns MD simulations (calc. −26.8 and −23.9 kcal/mol, respectively). In contrast, compounds eMol26313223 and eMol26314565 exhibited a decrease in the binding energies throughout the 50 ns MD simulations with Δ*G*_binding_ of −38.5 and −34.5 kcal/mol, respectively, compared to Δ*G*_binding_ = −15.4 kcal/mol for QNZ.

The calculated MM-GBSA binding energies were further decomposed into independent components. For compounds eMol26313223, eMol26314565, and QNZ, binding was observed in the mutated PBP2a allosteric site (Table 3) with a dominance of *E*_vdw_ forces with values of −50.8, −47.1, and −24.7 kcal/mol, respectively, with QNZ exhibiting approximately two times lower force than for the screened inhibitors. Additionally, electrostatic interactions (*E*_ele_) were observed with an average value of −24.9, −19.0, and −15.7 kcal/mol for eMol26313223, eMol26314565, and QNZ, respectively, in the mutated PBP2a allosteric site.

### 2.7. Post-Dynamics Analyses

To further examine the stability and behavior of eMol26313223 and eMol26314565 in complex with mutated PBP2a, structural and energetic analyses were performed throughout 50 ns MD simulations and compared to those of QNZ. Four features were evaluated from specific simulation trajectories, involving root-mean-square deviation (RMSD), hydrogen bond length, center-of-mass (CoM) distance, and binding energy per frame.

#### 2.7.1. Binding Energy per Frame

**Figure 6:**
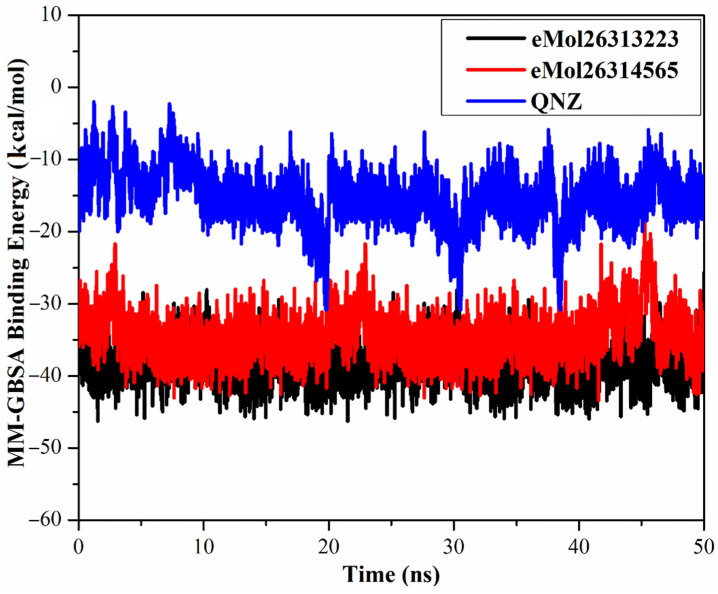
Estimated MM-GBSA binding energy per frame for eMol26313223 (in black), eMol26314565 (in red), and QNZ (in blue) with mutated PBP2a allosteric site in acidic medium throughout the 50 ns MD simulations.

To inspect the comprehensive structural stability of the identified inhibitors inside the allosteric site of the mutated PBP2a, a correlation between binding energy per frame and time was evaluated and plotted (Figure 6). Overall stabilities for eMol26313223, eMol26314565 and QNZ were observed with average binding energies (Δ*G*_binding_) of −38.4, −34.5, and −15.4 kcal/mol, respectively. The most obvious finding to emerge from the analysis is that all investigated complexes maintain stability over 50 ns MD simulations.

#### 2.7.2. Hydrogen Bond Analysis

Hydrogen bond analysis was performed to define the modality and period of hydrogen bonding between the target and identified inhibitors, as hydrogen bond interactions are pivotal in underpinning intermolecular specificity as well as in stabilizing target inhibitor complexes. Consequently, the specifics of the hydrogen bond distance and percentage occupancy were identified for eMol26313223- and eMol26314565-mutated PBP2a complexes and compared to the QNZ-mutated PBP2a complex over the 50 ns MD simulations (Table 4).

Both eMol26313223 and eMol26314565 formed stable hydrogen bonds with LYS316 with H-bond occupancies of 94.2% and 85.7%, respectively. This noticeably great H-bond occupancy points out the significance of LYS316 inside the allosteric site of the mutated PBP2a. Comparing the hydrogen bond distances of the investigated inhibitors with QNZ, it can be seen that eMol26313223 and eMol26314565 demonstrated a higher stability than QNZ. Again, eMol26313223 exhibited hydrogen bonding with LYS146, LYS273, and LYS316 with an average H-bond distance of 2.9, 2.9, and 2.7 Å, respectively. Similarly, a QNZ hydrogen bond was observed of LYS316 with an average H-bond distance of 2.6 and manifested H-bond occupancy of 79.7%. In addition, QNZ performed an intermediate stable hydrogen bond with GLU294 with an average value of 2.8 Å with a H-bond occupancy of 50.9%.

#### 2.7.3. Center-of-Mass Distance

**Figure 7:**
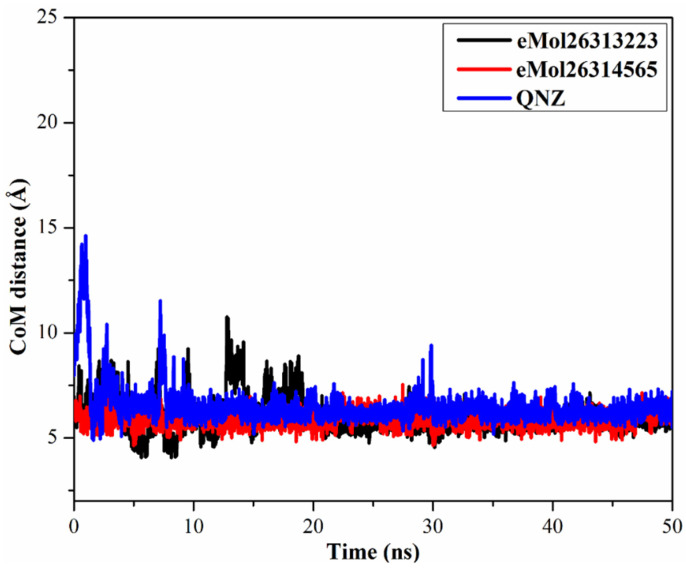
Center-of-mass (CoM) distances (in Å) between eMol26313223 (in black), eMol26314565 (in red), and QNZ (in blue) and LYS273 of mutated PBP2a allosteric site in acidic medium throughout the 50 ns MD simulations.

To obtain a more in-depth insight into the stability of inhibitor-mutated PBP2a throughout the 50 ns MD simulations, center-of-mass (CoM) distances were inspected between eMol26313223, eMol26314565, and QNZ and LYS316 (Figure 7). CoM distances were more narrow-fluctuated for eMol26313223 and eMol26314565 complexed with mutated PBP2a than for QNZ, with average values of 6.0, 5.9, and 7.5 Å, respectively. In summary, these results show that the identified inhibitors bind more tightly with the mutated PBP2a compared to QNZ. 

#### 2.7.4. Root-Mean-Square Deviation

**Figure 8:**
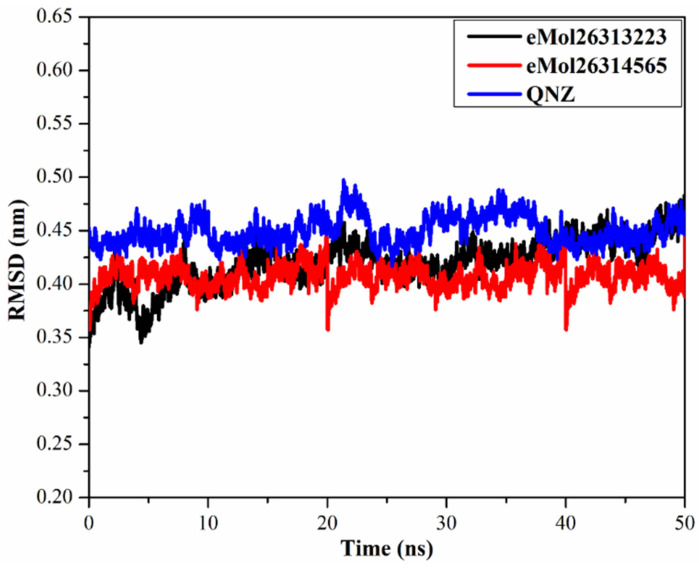
Root-mean-square deviation (RMSD) of the backbone atoms from the initial structure of eMol26313223 (in black), eMol26314565 (in red), and QNZ (in blue) towards mutated PBP2a allosteric site in acidic medium throughout the 50 ns MD simulations.

To observe the structural stability of the eMol26313223, eMol26314565, and QNZ-mutated PBP2a complexes, root-mean-square deviation (RMSD) values of the backbone atoms of the entire complex were calculated (Figure 8) with RMSD values remaining below 0.50 nm for the inspected complexes. Compounds eMol26313223, eMol26314565, and QNZ within the mutated PBP2a complex attained the fixed state within the first 10 ns of the simulation and remained stable for the remaining 40 ns simulations. These results suggest that eMol26313223 and eMol26314565 are tightly bound and not influenced by the topology of the mutated PBP2a.

### 2.8. In Silico ADMET Analysis

A pharmacodynamics study for the most potent inhibitors was performed using admetSAR to predict inhibitor action inside a host. The pharmacokinetic properties focus on absorption, distribution, metabolism, excretion, and toxicity (Table 5). Absorption (A) analysis revealed that eMol26313223, eMol26314565, and QNZ have high Caco-2 permeability, with human intestinal absorption (HIA) less than 30% recognized as poorly absorbed. P-glycoproteins with an ATP-binding trans-membrane component play a vital role in the secretion of incoming drugs from the cells [34]. While all inhibitors are non-substrates for P-glycoprotein, eMol26313223 and eMol26314565 are inhibitors of P-glycoprotein. In a distribution (D) analysis, metabolites that have a value of greater than 0.2 A are predicted to pass the blood-brain barrier (BBB permeability) readily [35]. In a metabolism (M) analysis, while CYP2D6 and CYP3A4 are leading cytochrome P450s enzymes that metabolize drugs in the liver [36], the investigated inhibitors were non-inhibitors of CYP2D6 and CYP3A4, except for eMol26314565, which was a substrate of CYP3A4. The excretion (E) of drugs is related to their hydrophilicity as well as molecular weight. Organic cation transporters (OCTs) and multidrug and toxin extrusion proteins (MATEs) in the kidney are the main carriers in the movement of cationic drugs into the urine [37]. Compounds eMol26313223 and eMol26314565 are predicted as non-inhibitors to MATE-1, OCT-1, and OCT-2, with such targeted inhibition confirming a potential safety trait. In the toxicity (T) analysis, eMol26313223 and eMol26314565 were non-carcinogenic and non-eye corrosive. In summary, the results demonstrate that the ADMET properties of the identified inhibitors showed robust binding, with some of the values comparable or better than the reference inhibitor (QNZ).

## 3. Computational Methodology

### 3.1. PBP2a Preparation

The X-ray resolved crystal structures of wild and double N146K and E150K mutated PBP2a (PDB codes: 4CJN (chain B) [21] and 4CPK (chain B) [20]) were selected as templates for molecular docking and molecular dynamics calculations. All missing residues were constructed using Modeller software [38]. Water molecules, ions, heteroatoms, and all ligands were eliminated. Finally, the protonation states of the wild and mutated PBP2a were then deliberated using a H++ server [39]. Additionally, all missing hydrogen atoms were inserted upon setting its parameters as follows: internal dielectric = 10, external dielectric = 80, salinity = 0.15, and pH = 5.5.

### 3.2. Resolved PBP2a Allosteric Inhibitors 

In the current study, two X-ray resolved PBP2a allosteric inhibitors—namely, 4-(2-{[(6*R*,7*R*)-2-carboxylato-7-[(2*Z*)-2-(ethoxyimino)-2-[5-(phosphonoamino)-1,2,4-thiadiazol-3-yl]acetamido]-8-oxo-5-thia-1-azabicyclo[4.2.0]oct-2-en-3-yl]sulfanyl}-1,3-thiazol-4-yl)-1-methylpyridin-1-ium (Ceftaroline/CFT) and (*E*)-3-(2-(4-cyanostyryl)-4-oxoquinazolin-3(4*H*)-yl) benzoic acid (QNZ)—were investigated. The 3D structures of CFT and QNZ were taken from resolved crystal structures with PDB codes of 3ZG0 [18] and 4CJN [21], respectively. The inhibitors were initially optimized at B3LYP/6-31G* level with the help of Gaussian09 software [40]. The atomic partial charges were assigned using the restrained electrostatic potential (RESP) approach [41].

### 3.3. Pharmacophore-Based Virtual Screening

**Figure 9:**
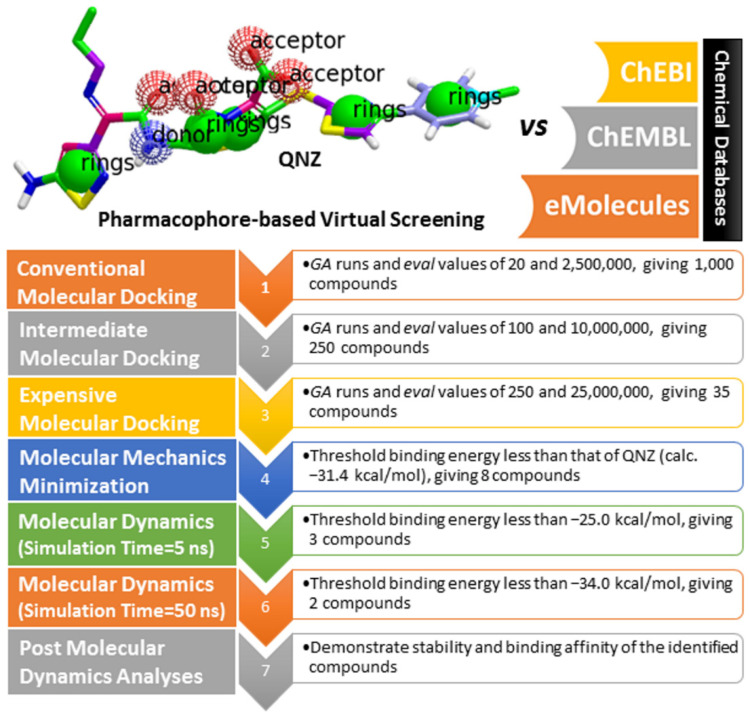
Schematic representation of the used *in silico* approaches as well as the filtration process.

To identify novel and potent inhibitors of double mutated PBP2a, a pharmacophore-based virtual screening was undertaken based on the physicochemical similarity to QNZ from the eMolecules (http://www.emolecules.com), ChEMBL [27], and ChEBI [28] databases containing more than 25 million compounds. The geometrical structures of unique inhibitors were then minimized using an MMFF94S force field with the assistance of SZYBKI software [42,43]. For each inhibitor, different conformations within an energy window 10 kcal/mol were generated using Omega2 software [44,45]. The protonation state and tautomer enumeration of the compounds were examined by fixpka and tautomer applications, respectively, included in the QUACPAC software [46]. The unionized state of the compounds was considered as in acidic medium. A pharmacophore-based virtual screening was then carried out on the generated conformations using ROCS software [29,30]. All parameters were kept in default mode, and ranking hits were executed using a Tanimoto combo scoring function [31]. A schematic representation of the used *in silico* techniques, in addition to the databases’ filtration process, is illustrated in Figure 9.

### 3.4. Molecular Docking

In the current study, three levels of molecular docking calculations—namely, conventional, intermediate, and expensive—were executed using AutoDock4.2.6 software [47]. For molecular docking calculations, the pdbqt files for the wild and double mutated PBP2a were prepared according to the AutoDock protocol [48]. All docking parameters were conserved to their default values, except the maximum number of energy evaluation (*eval*) and the number of genetic algorithms (*GA*) runs. The last-mentioned variables were adjusted to 20, 100 and 250, and 2,500,000, 10,000,000, and 25,000,000 for conventional, intermediate, and expensive, respectively. The docking grid was made to embrace the allosteric site for the PBP2a receptor with a grid size of 52 Å × 52 Å × 52 Å, and centered at XYZ coordinates 9.658, −1.662, and −70.269. The grid spacing value was adjusted to 0.375 Å. Gasteiger atomic partial charges were assigned for all investigated inhibitors [49]. The predicted binding modes for each inhibitor were adjusted using a built-in clustering analysis with an RMSD tolerance of 1.0 Å, and the lowest energy conformation from the largest cluster was selected as a representative binding pose. Throughout the selection of the most potent PBP2a inhibitors, duplicated compounds with identical InChIKey were removed [50].

### 3.5. Inhibitor-PBP2a Complex Minimization

All docked inhibitor-PBP2a complexes were submitted for molecular mechanical (MM) minimization with an RMSD value of 10^−9^ Å using a Sander code inside AMBER16 software [51]. In MM minimization, the truncated Newton linear conjugate gradient method with LBFGS pre-conditioning [52] was utilized in an implicit solvent using a generalized Born solvent model (igb = 1 [53]). AMBER force field 14SB [54] and a general AMBER force field (GAFF) [55] were used for the parameter assignment of PBP2a and the studied inhibitors, respectively. The restrained electrostatic potential (RESP) approach [41] was used to describe the atomic partial charges for the investigated inhibitors. No periodic boundary conditions were applied and a non-bonded cutoff of 999 Å was used.

### 3.6. Molecular Dynamics Simulations

All molecular dynamics (MD) simulations were run only on the most potent inhibitors using mutated PBP2a and AMBER16 software. The docked inhibitor-PBP2a complexes were first solvated with TIP3P water molecules and positioned in the center of a cubic box of size 15 × 15 × 15 A^3^ [56]. The solvated inhibitor-PBP2a complexes were then subjected to energy minimization for 5000 steps using a combination of steepest descent and conjugate gradient algorithms. The minimized systems were afterward smoothly heated from 0 to 300 K over 50 ps with a weak restraint of 10 kcal mol^−1^ Å^−1^ on the PBP2a protein. Further MD simulations were executed for 1000 ps to equilibrate the investigated complexes. Eventually, MD simulations for each inhibitor-PBP2a complex were conducted for 5 and 50 ns. In MD simulations, the Particle Mesh Ewald (PME) method was applied for treating the long-range electrostatic interactions under periodic conditions with a direct space cutoff of 12 Å [57]. In addition, the linear constraint solver (LINCS) algorithm was applied for covalent bond constraints [58]. In order to maintain the temperature at 298 K, Langevin dynamics with collision frequency gamma_ln set to 1.0 was applied. The pressure was controlled using a Berendsen barostat with a relaxation time of 2 ps [59]. Bonds involving hydrogen atoms were also constrained via a SHAKE algorithm with a time step of 2 fs [60]. All MD simulations were carried out using a CPU version of pmemd (pmemd.MPI) implemented in AMBER16 software. The Discovery Studio module of Biovia software was employed for 3D and 2D visualizations of the inhibitor-PBP2a interactions.

### 3.7. MM-GBSA Binding Energy

Binding energies of inhibitors with PBP2a protein were calculated using molecular mechanics-generalized born surface area (MM-GBSA) approach [61] with a GB model (igb = 2). The binding energies (Δ*G*_binding_) were computed using the molecular mechanics minimized complex as well as uncorrelated snapshots collected from the generated MD trajectories, given by:

(1) $$\Delta G_{\mathrm{binding}}=G_{\mathrm{Complex}}-\left(G_{\mathrm{Inhibitor}}+G_{\mathrm{PBP}2a}\right)$$

where the energy term (*G*) is estimated as:

(2) $$G=E_{\mathrm{vdw}}+E_{\mathrm{ele}}+G_{\mathrm{GB}}+G_{\mathrm{SA}}$$

With *E*_vdw_, *E*_ele_, *G*_GB_, and *G*_SA_ as the van der Waals, electrostatic, general Born solvation and surface area energies, respectively. For the inhibitors, entropy contributions were neglected.

### 3.8. In Silico ADMET Analysis

The AdmetSAR server (http://lmmd.ecust.edu.cn/admetsar2/) is an open access, *in silico* tool for predicting chemical pharmacokinetic properties [62]. An admetSAR server was utilized to predict the absorption, distribution, metabolism, excretion, and toxicity (ADMET) of the active PBP2a allosteric inhibitors. The absorption (A) of perfect drugs relies on agents like membrane permeability (created by colon cancer cell line (Caco-2)), human intestinal absorption (HIA), and the status of either P-glycoprotein substrate or inhibitor. The distribution (D) of drugs fundamentally counts on the capability of passing the blood-brain barrier (BBB). The metabolism (M) of drugs is estimated by the CYP models. Excretion (E) of the drugs is calculated according to the renal OCT substrate. Thereafter, the toxicity (T) of the drugs is forecasted on the Human Ether-a-go-go-related gene inhibition, mutagenic status, carcinogenic status, and acute oral toxicity [63].

## 4. Conclusions

Mutations in a penicillin-binding protein 2a (PBP2a) is key to *β*-lactam antibiotic resistance in *Staphylococcus aureus*. To identify novel allosteric inhibitors against mutated PBP2a in acidic medium and to explore potential antibacterial effects against methicillin-resistant *Staphylococcus aureus* (MRSA), chemical databases were screened to identify inhibitors with physicochemical similarity to co-crystallized QNZ ligand using ROCS software. Compounds eMol26313223 and eMol26314565 were identified based on molecular docking and molecular minimization calculations, and so were MM-GBSA binding energies for these two compounds in complex with mutated PBP2a. On the basis of MM-GBSA energies, eMol26313223 and eMol26314565 demonstrated promising binding affinities towards mutated PBP2a with binding energies double that of QNZ. Post-dynamics analyses manifested the high stability and binding affinity of the two identified inhibitors with mutated PBP2a. The ADMET predictions revealed that the identified inhibitors are non-toxic and demonstrate high oral absorption. The current results underscore eMol26313223 and eMol26314565 as promising inhibitors towards mutated PBP2a and hold promise for *in vitro* and *in vivo* inhibition studies with antibiotic-resistant bacterial strains.

## Figures and Tables

**Table 1:** Estimated MM-GBSA//MM and MM-GBSA//MD binding energies of QNZ with wild and mutated PBP2a allosteric site in acidic medium.

PBP2a	MM-GBSA//MM ^a^ (kcal/mol)	Calculated MM-GBSA Binding Energy (kcal/mol) ^b^						
		∆*E*_vdw_	∆*E*_ele_	∆*E*_GB_	∆*E*_SUR_	∆*G*_gas_	∆*G*_Solv_	∆*G*_binding_
Wild	−30.5	−25.3	−9.4	22.5	−3.2	−34.6	19.3	−15.3
Mutated	−31.4	−24.7	−15.7	28.3	−3.3	−40.4	25.0	−15.4

^a^ MM-GBSA//MM refers to MM-GBSA binding energies calculated based on the molecular minimized QNZ-PBP2a complexes. ^b^ MM-GBSA//MD refers to MM-GBSA binding energies calculated over 50 ns MD simulations.

**Table 2:** Estimated conventional, intermediate, and expensive docking scores and 2D chemical structures for QNZ and the top 35 potent inhibitors against mutated PBP2a allosteric site in acidic medium ^a^.

Compound Name/Code	Chemical Structure	Docking Score (kcal/mol)			Compound Name/Code	Chemical Structure	Docking Score (kcal/mol)		
		Conv. ^b^	Inter. ^c^	Exp. ^d^			Conv. ^b^	Inter. ^c^	Exp. ^d^
QNZ	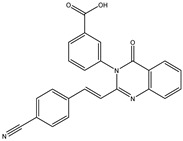	−6.9	−8.0	−8.3	eMol29597031	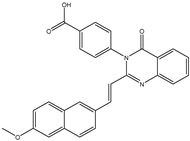	−9.2	−9.3	−9.3
eMol26313223	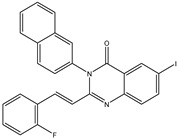	−8.3	−8.4	−10.0	eMol26269394	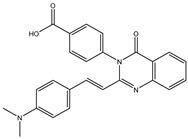	−8.5	−8.7	−9.1
eMol26437582	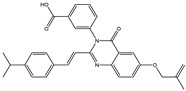	−8.2	−8.9	−9.9	eMol27202760	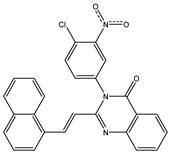	−8.0	−8.1	−9.1
eMol26314565	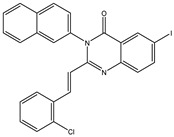	−8.2	−8.2	−9.6	eMol26242018	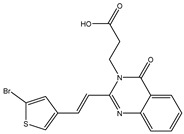	−8.0	−8.8	−9.0
eMol26313117	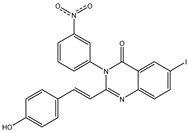	−8.9	−9.4	−9.5	eMol29633913	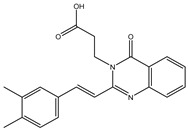	−7.8	−8.6	−8.9
eMol26293960	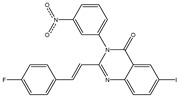	−8.1	−9.2	−9.4	CHEMBL1215080	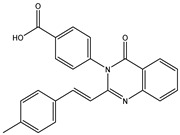	−8.4	−8.4	−8.9
eMol3021959	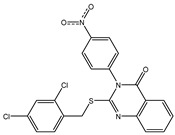	−7.1	−8.9	−9.4	eMol30017880	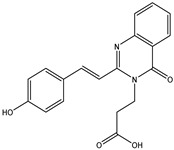	−7.4	−8.3	−8.7
eMol27252412	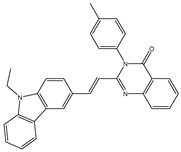	−8.1	−8.2	−8.7	eMol29634797	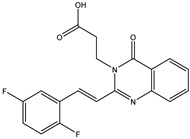	−7.6	−8.1	−8.6
eMol300094331	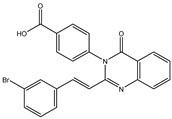	−8.2	−8.3	−8.7	eMol27202252	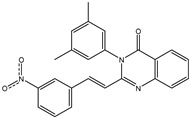	−8.0	−8.1	−8.6
eMol26264570	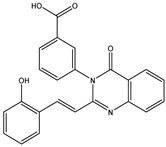	−8.1	−8.4	−8.7	eMol26262168	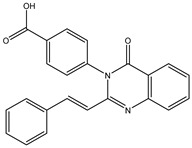	−7.0	−8.0	−8.5
eMol26319231	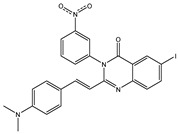	−8.2	−8.6	−8.7	CHEMBL1215082	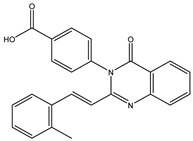	−7.4	−8.0	−8.5
eMol299980544	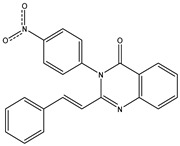	−8.2	−8.2	−8.6	eMol26242042	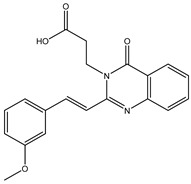	−7.6	−8.5	−8.5
eMol27406062	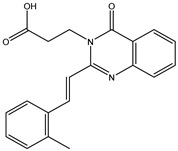	−7.8	−8.3	−8.6	eMol300154219	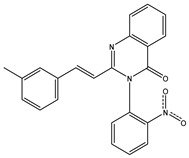	−7.3	−8.3	−8.5
eMol29565259	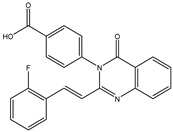	−7.8	−8.2	−8.6	eMol27091498	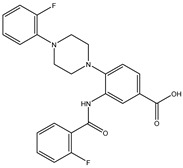	−8.0	−8.1	−8.4
eMol27202248	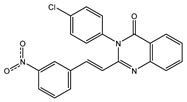	−8.4	−8.3	−8.6	eMol26242026	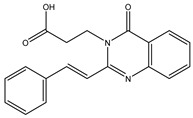	−7.8	−8.2	−8.4
CHEMBL1215004	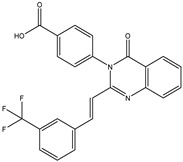	−7.7	−8.2	−8.4	eMol301527162	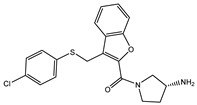	−8.0	−8.1	−8.3
eMol26330545	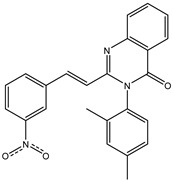	−8.2	−8.2	−8.4	eMol27202948	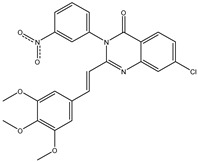	−7.4	−8.0	−8.3
eMol26314779	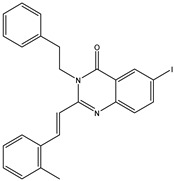	−8.1	−8.2	−8.3	eMol26314779	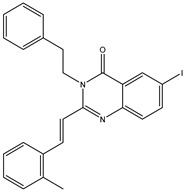	−8.1	−8.2	−8.3

^a^ Data ranked based on the expensive docking scores. ^b^ Conv. refers to the conventional docking calculation. ^c^ Inter. refers to the intermediate docking calculation. ^d^ exp. refers to the expensive docking calculation.

**Table 3:** Components of the MM-GBSA binding energies for eMol26313223, eMol26314565, and QNZ complexed with mutated PBP2a allosteric site in acidic medium throughout the MD course of 50 ns.

Compound Name/Code	Estimated MM-GBSA Binding Energy (kcal/mol)						
	∆*E*_vdw_	∆*E*_ele_	∆*E*_GB_	∆*E*_SUR_	∆*G*_gas_	∆*G*_solv_	∆*G_binding_*
QNZ	−24.7	−15.7	28.3	−3.3	−40.4	25.0	−15.4
eMol26313223	−50.8	−26.9	44.9	−5.6	−73.3	39.3	−38.4
eMol26314565	−47.1	−19.0	36.8	−5.2	−64.3	31.6	−34.5

**Table 4:** Hydrogen bonds demonstrated between the identified inhibitors and the key amino acid residues inside the allosteric site of the mutated PBP2a in acidic medium.

Compound Name/Code	Acceptor	Donor	Distance (Å) ^a^	Angle (Degree) ^a^	Occupied (%) ^b^
QNZ	GLU294@O	QNZ@O-H	2.8	160	50.9
	QNZ@O	LYS316@N-H	2.6	151	79.7
eMol26313223	eMol26313223@O	LYS146@N-H	2.9	141	60.6
	eMol26313223@N	LYS273@N-H	2.9	148	90.0
	eMol26313223@F	LYS316@N-H	2.7	162	94.2
eMol26314565	eMol26314565@O	LYS146@N-H	2.9	141	52.0
	eMol26314565@N	LYS273@N-H	2.9	139	54.8
	eMol26314565@N	LYS316@N-H	3.0	143	85.7

^a^ The hydrogen bonds are scrutinized by the acceptor-donor atom distance of <3.5 Å as well as acceptor-H-donor angle of >120°. ^b^ Only hydrogen bonds with occupancy greater than 50% were noticed.

**Table 5:** Predicted ADMET properties of the top potent inhibitors.

ADME Parameters	QNZ	eMol26313223	eMol26314565
Absorption			
Human Intestinal Absorption (% Absorbed)	+	+	+
	+ 98.3%	+ 99.4%	+ 99.4%
P-glycoprotein Inhibitor	−	+	+
P-glycoprotein Substrate	−	−	−
Distribution			
Blood–Brain Barrier	+	+	+
Subcellular localization	Mitochondria	Mitochondria	Mitochondria
Metabolism			
CYP450 2D6 Inhibition	−	−	−
CYP450 2D6 Substrate	−	−	−
CYP450 3A4 Inhibition	−	−	−
CYP450 3A4 Substrate	−	−	+
Excretion			
OCT1 Inhibitor	−	−	−
OCT2 Inhibitor	−	−	−
MATE1 Inhibitor	−	−	−
Toxicity			
Carcinogens	−	−	−
Acute Toxicity (Class)	II	III	III
Eye corrosion	−	−	−
Eye irritation	−	−	−
Human Ether-a-go-go-Related Inhibition	−	−	−

## Data Availability

The data presented in this study are available in the article/supplementary material.

## Supplementary Materials

The following are available online at https://www.mdpi.com/article/10.3390/antibiotics10080934/s1. Figure S1: 3D representations of the predicted binding modes of ionized (in gray) and unionized (in cyan) QNZ complexed with the PBP2a inside the allosteric site. Figure S2: 3D and 2D representations of the experimental binding modes of CFT complexed with the PBP2a inside the allosteric site of chains A and B (PDB code: 3ZG0). Figure S3: (a) 3D representations of the docked structures of QNZ (in cyan) and CFT (in gray) and 2D representations of the predicted binding modes of (b) QNZ and (c) CFT complexed with the PBP2a inside the allosteric site in acidic medium. Figure S4: root-mean-square deviation (RMSD) of the backbone atoms from the initial structure of QNZ with wild (in black), and double mutated PBP2a allosteric site (in red) in acidic medium throughout the 50 ns MD simulations. Figure S5: Estimated MM-GBSA binding energy per frame for QNZ with wild (in black) and mutated PBP2a allosteric site (in red) in acidic medium throughout the 50 ns MD simulations. Figure S6: 2D representations of the predicted docked binding modes of the top 35 potent inhibitors in complex with mutated PBP2a allosteric site in acidic medium. Figure S7: 3D representations of AMBER-based minimized structures of the eight potent molecules and QNZ in complex with the mutated PBP2a in acidic medium. Table S1: calculated conventional docking scores (in kcal/mol) for QNZ and the top 5000 potent allosteric inhibitors against mutated PBP2a allosteric site in acidic medium. Table S2: estimated conventional and intermediate docking scores (in kcal/mol) for QNZ and the top 1000 potent allosteric inhibitors against mutated PBP2a allosteric site in acidic medium. Table S3: estimated conventional, intermediate, and expensive docking scores for QNZ and the top 250 potent allosteric inhibitors against mutated PBP2a allosteric site in acidic medium. Table S4: estimated conventional, intermediate, and expensive docking scores, MM-GBSA//MM binding energies, and binding features of AMBER-based minimized structures for QNZ and the top 35 potent inhibitors against mutated PBP2a allosteric site in acidic medium.
