# Supplementary material for: Non-β-Lactam Allosteric Inhibitors Target Methicillin-Resistant Staphylococcus aureus: An In Silico Drug Discovery Study
Source: Antibiotics (Basel). 2021 Aug 1;10(8):934. doi: 10.3390/antibiotics10080934 (PMC8388891; doi:10.3390/antibiotics10080934)
Supplement: Supplementary file 1 [file antibiotics-10-00934-s001.zip › antibiotics-1252106-supplementary.pdf]

Non-*β*-Lactam Allosteric Inhibitors Target Methicillin-Resistant *Staphylococcus aureus*: An *In Silico* Drug Discovery Study

Mahmoud A. A. Ibrahim [1,*], Khlood A. A. Abdeljawaad [1], Alaa H. M. Abdelrahman [1], Othman R. Alzahrani [2], Fahad M. Alshabrmi [3], Esraa Khalaf [4], Mahmoud F. Moustafa [5,6], Faris Alrumaihi [3], Khaled S. Allemailem [3], Mahmoud E. S. Soliman [7], Paul W. Paré [8], Mohamed-Elamir F. Hegazy [9,10,*] and Mohamed A. M. Atia [11,*]

1 Computational Chemistry Laboratory, Chemistry Department, Faculty of Science, Minia University, Minia 61519, Egypt; kh.abdeljawaad@compchem.net (K.A.A.A); a.abdelrahman@compchem.net (A.H.M.A.)
2 Department of Biology, Faculty of Sciences, University of Tabuk, Tabuk 71491, Saudi Arabia; O-alzahrani@ut.edu.sa (O.R.A.)
3 Department of Medical Laboratories, College of Applied Medical Sciences, Qassim University, Buraydah 51452, Saudi Arabia; fshbrmy@qu.edu.sa (F.M.A.); f_alrumaihi@qu.edu.sa (F.A.); k.allemailem@qu.edu.sa (K.S.A.)
4 Department of Bacteriology, Mycology and Immunology, Faculty of Veterinary Medicine, Beni-Suef University, Beni-Suef 62511, Egypt; dresraa.fathy@yahoo.com (E.K.)
5 Department of Biology, College of Science, King Khalid University, Abha 9004, Saudi Arabia; hamdony@yahoo.com or mfmostfa@kku.edu.sa (M.F.M.)
6 Department of Botany & Microbiology, Faculty of Science, South Valley University, Qena 83523, Egypt
7 Molecular Bio-computation and Drug Design Lab, School of Health Sciences, University of KwaZulu-Natal, Westville, Durban 4000, South Africa; soliman@ukzn.ac.za (M.E.S.S.)
8 Department of Chemistry and Biochemistry, Texas Tech University, Lubbock, TX 79409, USA; paul.pare@ttu.edu (P.W.P.)
9 Chemistry of Medicinal Plants Department, National Research Centre, Giza 12622, Egypt (M.E.F.H.)
10 Department of Pharmaceutical Biology, Institute of Pharmaceutical and Biomedical Sciences, Johannes Gutenberg University, Mainz 55128, Germany
11 Molecular Genetics and Genome Mapping Laboratory, Genome Mapping Department, Agricultural Genetic Engineering Research Institute (AGERI), Agricultural Research Center (ARC), Giza 12619, Egypt (M.A.M.A.)
* Correspondence: m.ibrahim@compchem.net (M.A.A.I.); mohegazy@uni-mainz.de (M.E.F.H.); matia@ageri.sci.eg (M.A.M.A); Tel.: +2010-241-61-444 (M.A.A.I.); +2-033-371-635 (M.E.F.H.); +2-010-001-64-922 (M.A.M.A.)

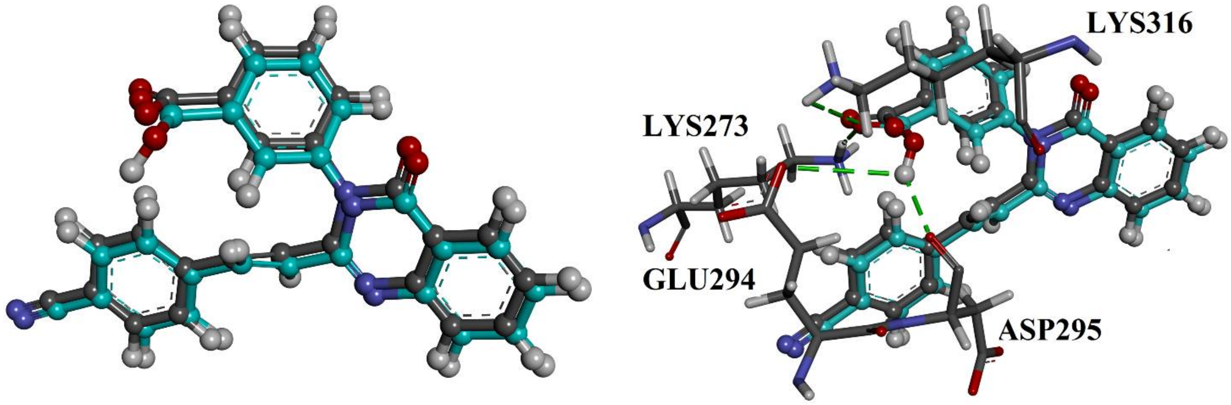

Figure S1. 3D representations of the predicted binding modes of ionized (in gray) and unionized (in cyan) QNZ complexed with the PBP2a inside the allosteric site.

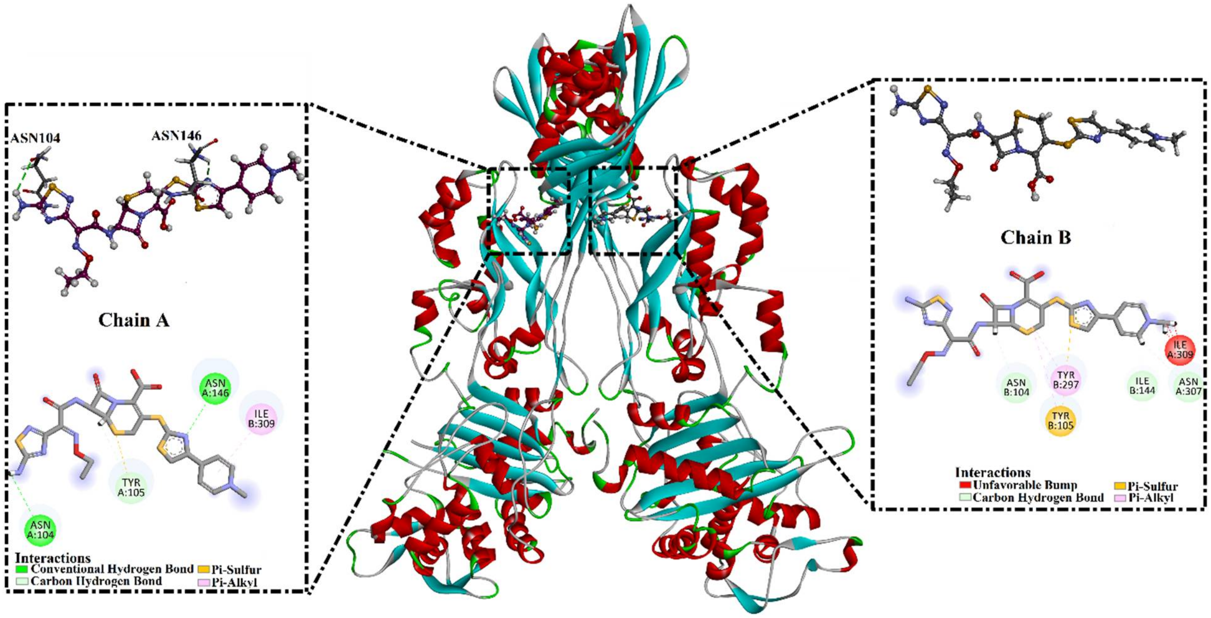

Figure S2. 3D and 2D representations of the experimental binding modes of CFT complexed with the PBP2a inside the allosteric site of chains A and B (PDB code: 3ZG0).

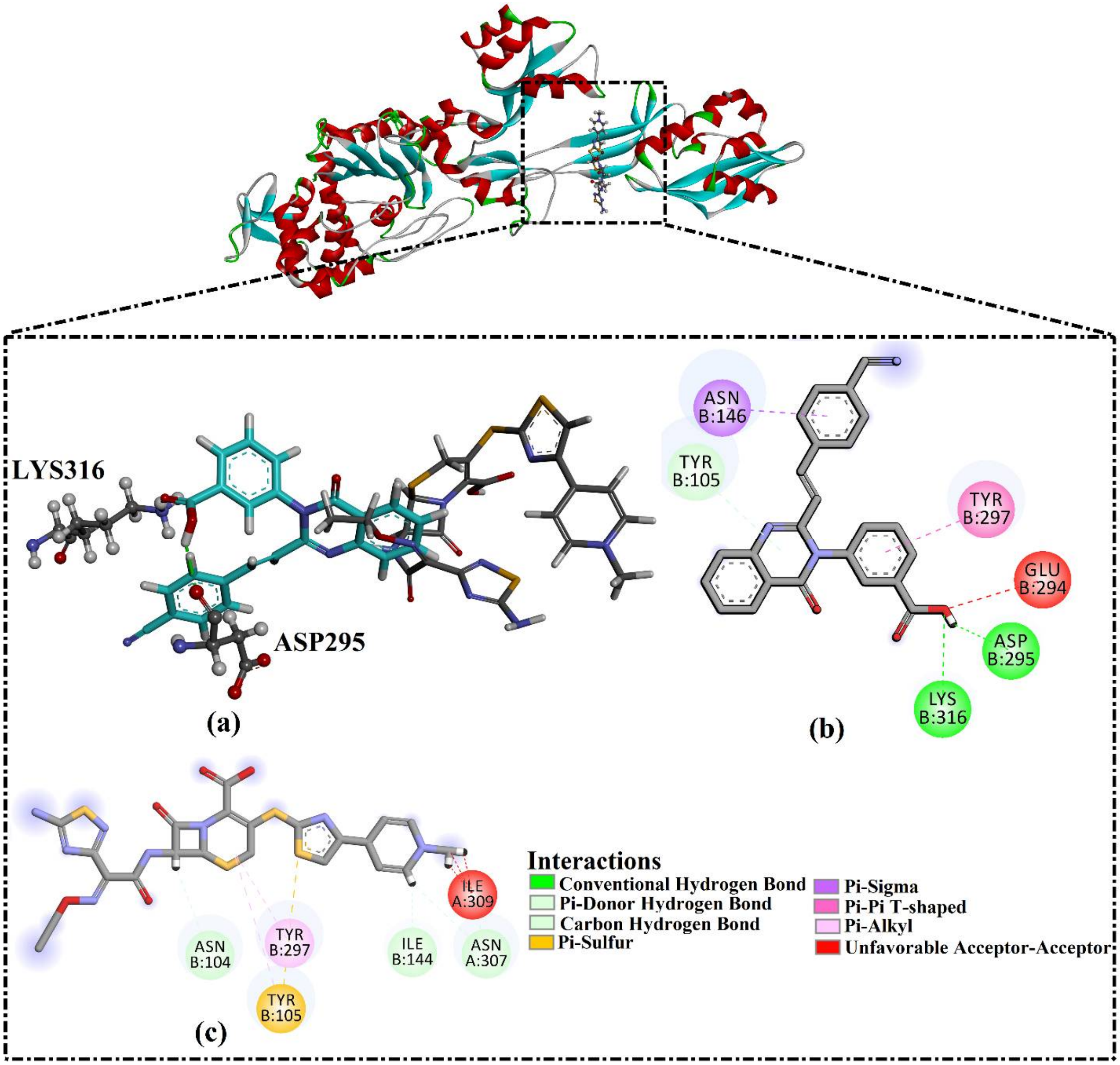

Figure S3: (a) 3D representations of the docked structures of QNZ (in cyan) and CFT (in gray) and 2D representations of the predicted binding modes of (b) QNZ and (c) CFT complexed with the PBP2a inside the allosteric site in acidic medium.

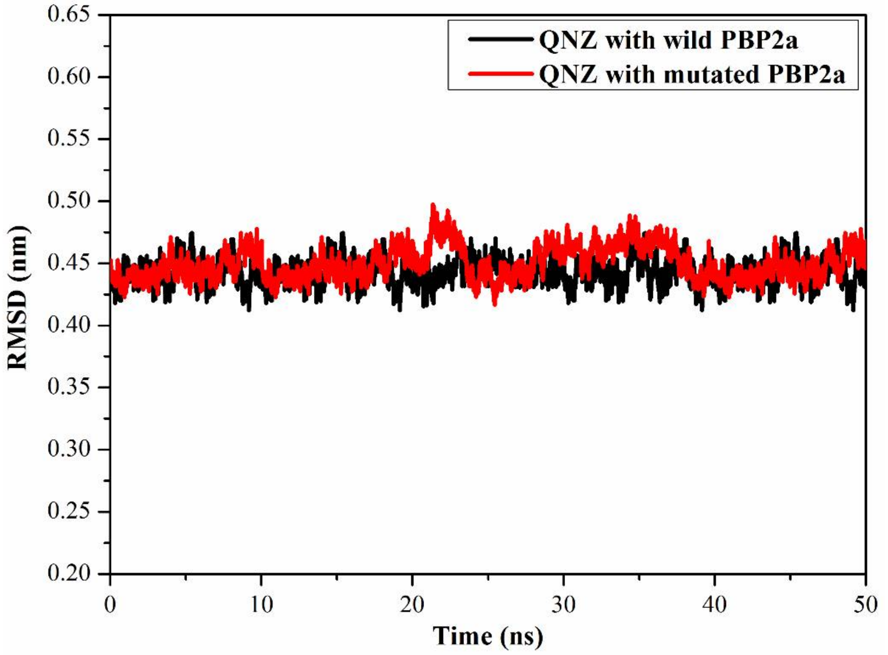

Figure S4. Root-mean-square deviation (RMSD) of the backbone atoms from the initial structure of QNZ with wild (in black), and double mutated PBP2a allosteric site (in red) in acidic medium throughout the 50 ns MD simulations.

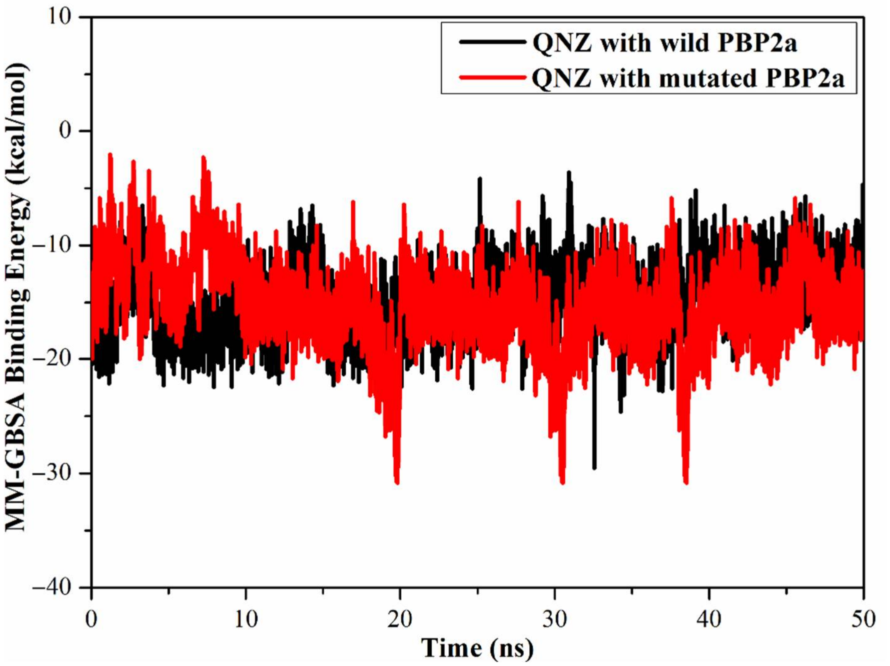

Figure S5. Estimated MM-GBSA binding energy per frame for QNZ with wild (in black) and mutated PBP2a allosteric site (in red) in acidic medium throughout the 50 ns MD simulations.

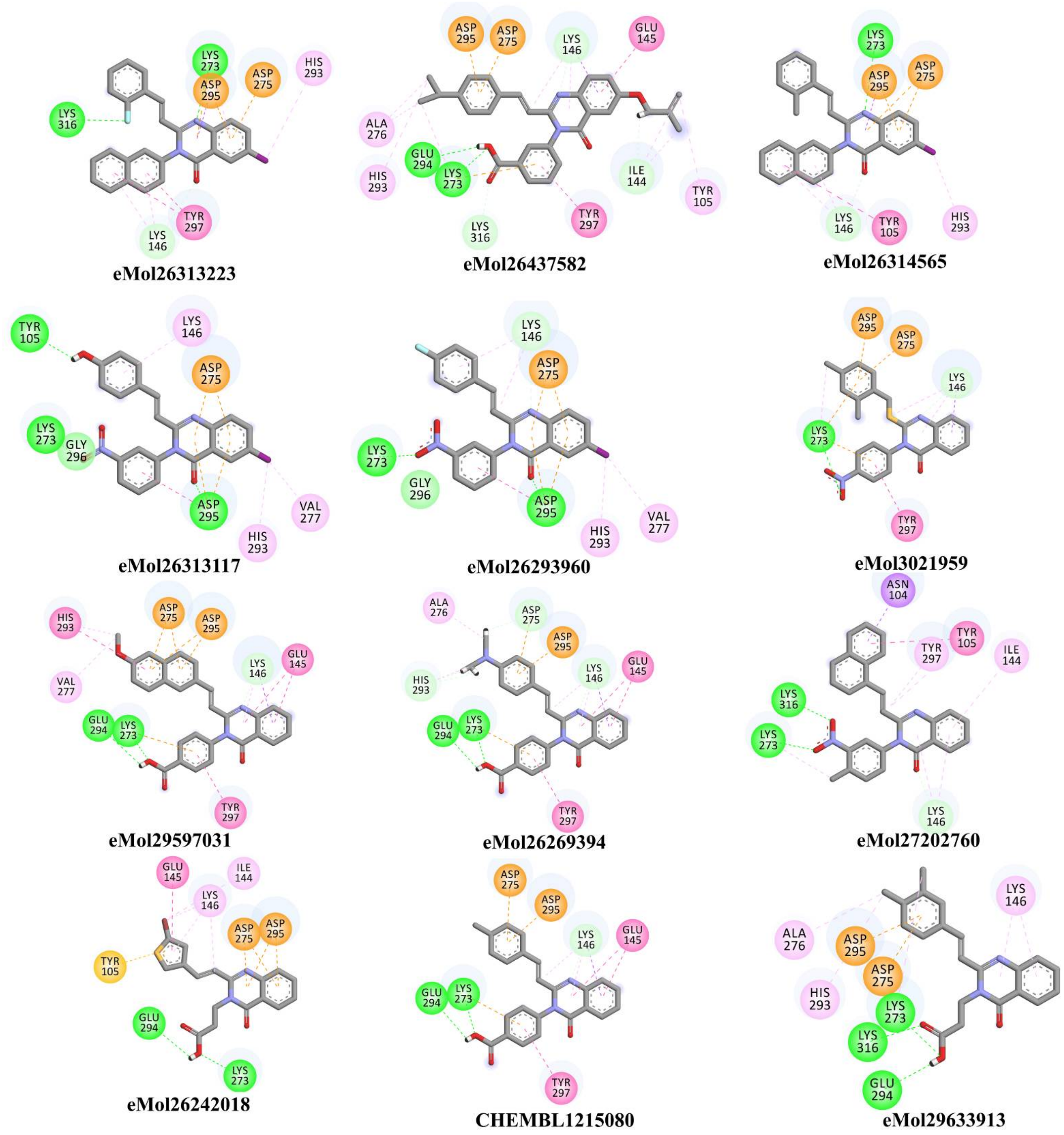

Figure S6. 2D representations of the predicted docked binding modes of the top 35 potent inhibitors in complex with mutated PBP2a allosteric site in acidic medium.

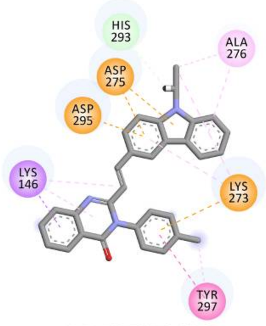

eMol27252412

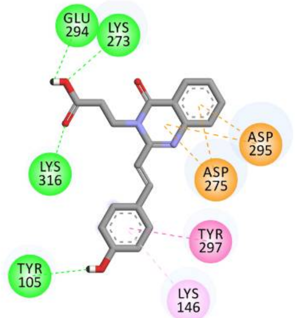

eMol30017880

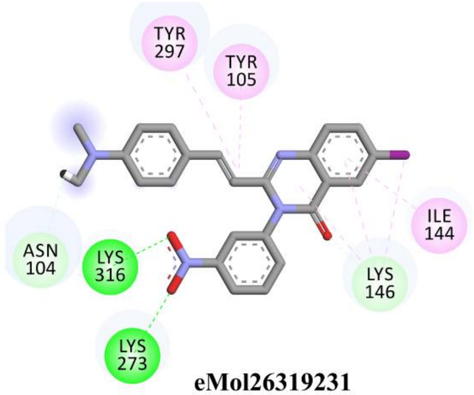

eMol26319231

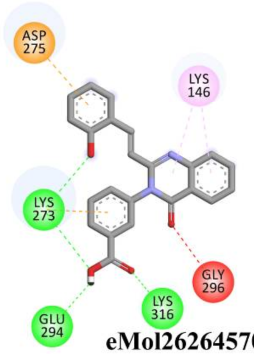

eMol26264570

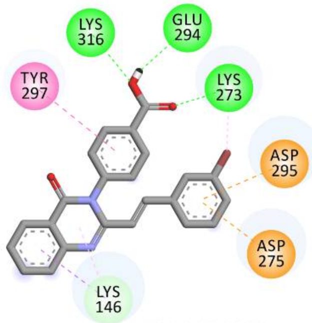

eMol300094331

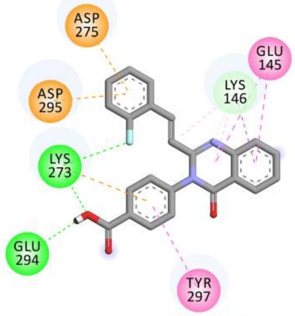

eMol29565259

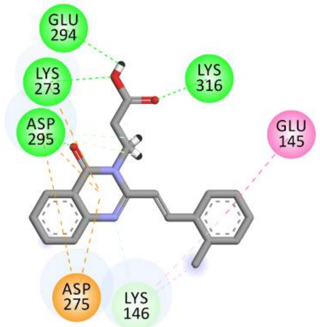

eMol27406062

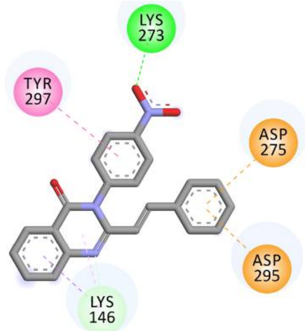

eMol299980544

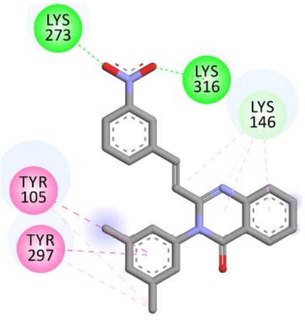

eMol27202252

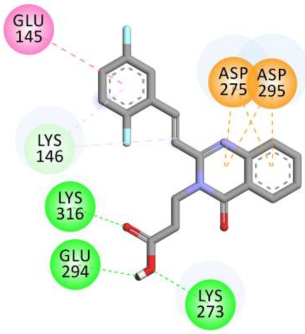

eMol29634797

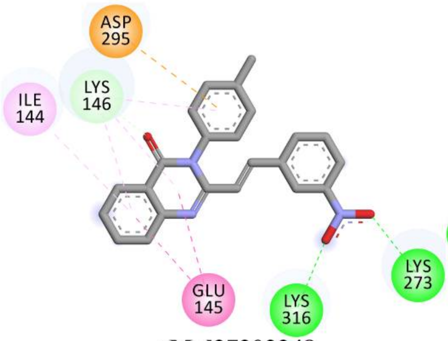

eMol27202248

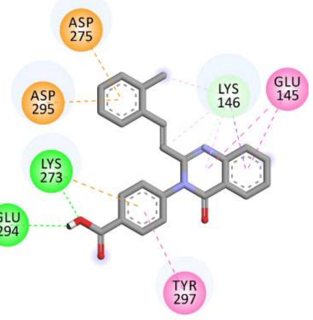

CHEMBL1215082

Figure S6. *Continued.*

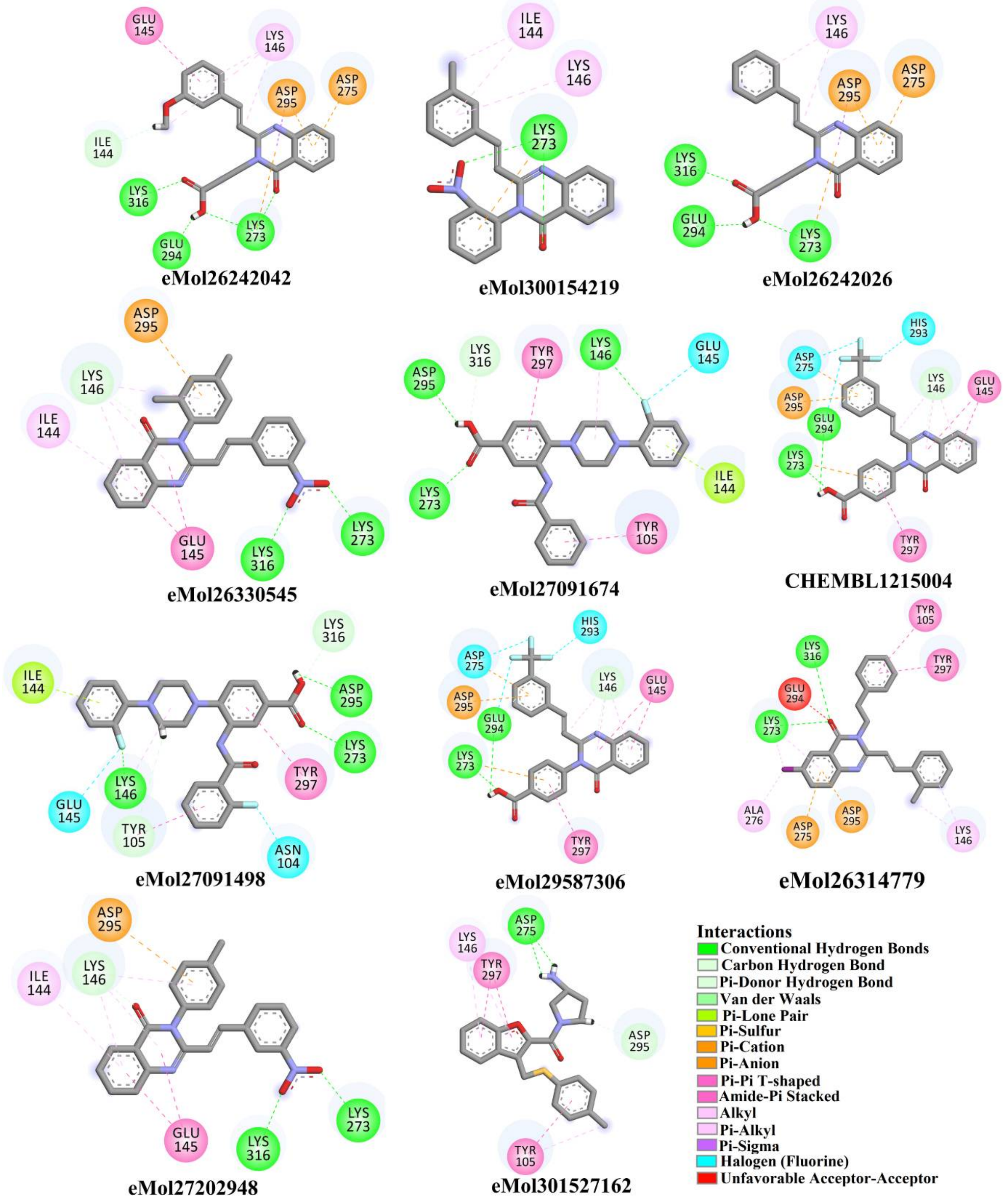

Figure S6. *Continued.*

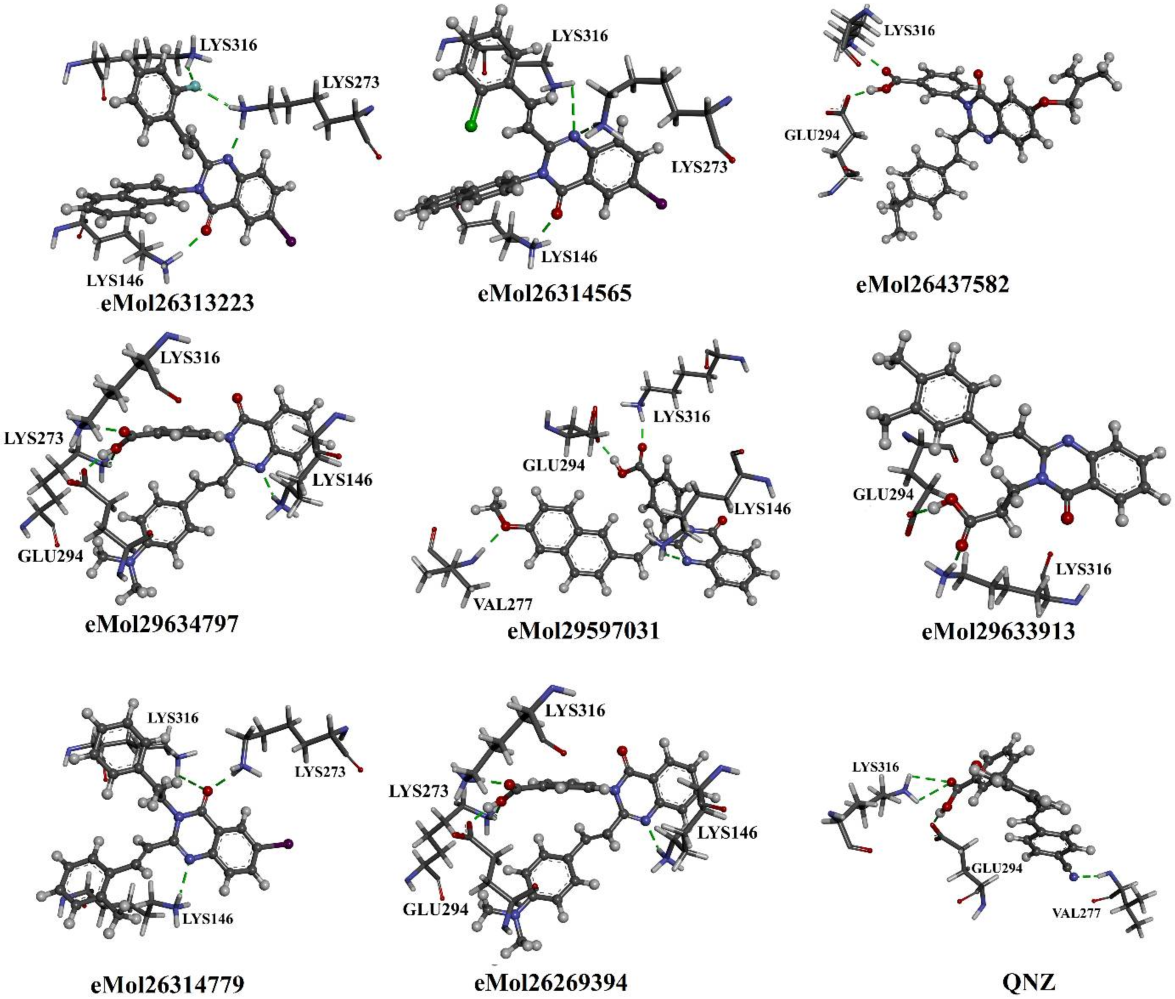

Figure S7. 3D representations of AMBER-based minimized structures of the eight potent molecules and QNZ in complex with the mutated PBP2a in acidic medium.

Table S1. Calculated conventional docking scores (in kcal/mol) for QNZ and the top 5000 potent allosteric inhibitors against mutated PBP2a allosteric site in acidic medium.

No.	Compound Name/Code	Docking Score (kcal/mol)	No.	Compound Name/Code	Docking Score (kcal/mol)	No.	Compound Name/Code	Docking Score (kcal/mol)
	QNZ	−6.9	49	eMol23246696	−8.5	98	eMol45585832	−8.2
1	eMol30323345	−9.8	50	eMol23157132	−8.4	99	eMol956623	−8.2
2	CHEMBL1214717	−9.4	51	CHEMBL3818377	−8.4	100	CHEMBL3818775	−8.2
3	eMol29587310	−9.4	52	eMol31786896	−8.4	101	eMol46480173	−8.2
4	eMol45585846	−9.4	53	eMol19076714	−8.4	102	eMol24530483	−8.2
5	eMol23239806	−9.3	54	eMol579568	−8.4	103	eMol26437582	−8.2
6	CHEMBL1214718	−9.2	55	eMol300994534	−8.4	104	eMol6263471	−8.2
7	eMol29597031	−9.2	56	eMol48938145	−8.4	105	eMol681936	−8.2
8	eMol45585848	−9.2	57	eMol18962382	−8.4	106	CHEMBL1558437	−8.2
9	eMol23186178	−9.1	58	eMol24519844	−8.4	107	eMol26809774	−8.2
10	eMol18542844	−9.1	59	eMol27202240	−8.4	108	eMol26330545	−8.2
11	eMol30323633	−9.0	60	eMol27202248	−8.4	109	CHEMBL3818929	−8.2
12	CHEMBL1534883	−9.0	61	CHEMBL1215080	−8.4	110	eMol1088331	−8.2
13	eMol1344114	−9.0	62	eMol43906059	−8.4	111	eMol26314565	−8.2
14	eMol24519694	−9.0	63	CHEMBL4103504	−8.3	112	eMol26319231	−8.2
15	eMol19076718	−8.9	64	eMol27202910	−8.3	113	eMol46480165	−8.2
16	eMol26313117	−8.9	65	eMol31532412	−8.3	114	eMol20347873	−8.2
17	eMol19076712	−8.9	66	CHEMBL395193	−8.3	115	eMol206926236	−8.2
18	CHEMBL1214794	−8.9	67	eMol23239141	−8.3	116	eMol300094331	−8.2
19	eMol26196290	−8.9	68	eMol26313223	−8.3	117	eMol23178762	−8.1
20	eMol45585824	−8.9	69	CHEMBL3805924	−8.3	118	eMol4518648	−8.1
21	CHEMBL1214720	−8.8	70	eMol23181799	−8.3	119	CHEMBL1214935	−8.1
22	eMol30341049	−8.8	71	eMol23175530	−8.3	120	eMol18550846	−8.1
23	eMol43906045	−8.8	72	CHEMBL3818584	−8.3	121	eMol43906051	−8.1
24	eMol20379909	−8.8	73	eMol18542848	−8.3	122	eMol566574	−8.1
25	eMol18542846	−8.8	74	eMol27202258	−8.3	123	CHEMBL3819445	−8.1
26	eMol20337486	−8.8	75	eMol679826	−8.3	124	eMol20368046	−8.1
27	eMol20378769	−8.7	76	eMol25810071	−8.3	125	eMol27252412	−8.1
28	eMol31781482	−8.7	77	eMol25810075	−8.3	126	CHEMBL1214647	−8.1
29	eMol2403964	−8.7	78	eMol25810083	−8.3	127	CHEMBL508966	−8.1
30	eMol1166007	−8.7	79	eMol26242044	−8.3	128	eMol2992482	−8.1
31	CHEMBL1214938	−8.7	80	eMol193446288	−8.3	129	eMol46490873	−8.1
32	eMol20375178	−8.7	81	eMol25819217	−8.3	130	CHEMBL3817888	−8.1
33	CHEMBL1214941	−8.6	82	CHEMBL1215003	−8.2	131	eMol26314779	−8.1
34	eMol19076754	−8.6	83	CHEMBL518358	−8.2	132	eMol27202956	−8.1
35	eMol19076742	−8.6	84	eMol29653471	−8.2	133	eMol566488	−8.1
36	eMol23241028	−8.6	85	eMol43906053	−8.2	134	eMol20361212	−8.1
37	eMol30323569	−8.6	86	eMol45622745	−8.2	135	eMol27202348	−8.1
38	CHEMBL1215648	−8.6	87	eMol587826	−8.2	136	eMol2992792	−8.1
39	eMol26196326	−8.6	88	eMol588192	−8.2	137	eMol26264570	−8.1
40	eMol45585842	−8.6	89	CHEMBL1214713	−8.2	138	eMol26293960	−8.1
41	eMol565168	−8.6	90	CHEMBL1384683	−8.2	139	eMol565798	−8.1
42	CHEMBL1215650	−8.5	91	eMol23243407	−8.2	140	eMol11920256	−8.1
43	CHEMBL1579061	−8.5	92	eMol26270654	−8.2	141	eMol4562556	−8.1
44	eMol26269394	−8.5	93	eMol46365626	−8.2	142	eMol16797514	−8.1
45	CHEMBL1215649	−8.5	94	CHEMBL1214936	−8.2	143	eMol26242050	−8.1
46	eMol22447774	−8.5	95	eMol26196312	−8.2	144	eMol23147497	−8.1
47	eMol26196316	−8.5	96	eMol26242034	−8.2	145	CHEMBL1443074	−8.0
48	CHEMBL3818129	−8.5	97	eMol299980544	−8.2	146	eMol18940433	−8.0

Table S1. *Continued.*

No.	Compound Name/Code	Docking Score (kcal/mol)	No.	Compound Name/Code	Docking Score (kcal/mol)	No.	Compound Name/Code	Docking Score (kcal/mol)
147	eMol26242018	−8.0	196	eMol1035565	−7.9	245	eMol4552457	−7.8
148	eMol27202760	−8.0	197	eMol26242038	−7.9	246	eMol679554	−7.8
149	eMol30248263	−8.0	198	eMol31297102	−7.9	247	CHEMBL1212961	−7.8
150	eMol43731200	−8.0	199	eMol4381378	−7.9	248	eMol18550688	−7.8
151	eMol679556	−8.0	200	eMol679552	−7.9	249	eMol24313582	−7.8
152	eMol679822	−8.0	201	eMol19266609	−7.9	250	eMol27406062	−7.8
153	CHEMBL1215797	−8.0	202	eMol23190351	−7.9	251	eMol300136046	−7.8
154	eMol20377597	−8.0	203	eMol26242032	−7.9	252	eMol314064301	−7.8
155	eMol27091498	−8.0	204	eMol30671942	−7.9	253	eMol46480212	−7.8
156	eMol4397966	−8.0	205	eMol33366267	−7.9	254	eMol566288	−7.8
157	eMol4577396	−8.0	206	eMol36912832	−7.9	255	eMol26315830	−7.8
158	CHEMBL1214648	−8.0	207	eMol46363574	−7.9	256	eMol4579154	−7.8
159	CHEMBL3818572	−8.0	208	eMol75958504	−7.9	257	eMol46367311	−7.8
160	eMol13856171	−8.0	209	eMol587628	−7.9	258	CHEMBL3819597	−7.8
161	eMol27091674	−8.0	210	eMol1035521	−7.9	259	eMol206970196	−7.8
162	eMol43906055	−8.0	211	eMol20342278	−7.9	260	eMol29633913	−7.8
163	eMol4558540	−8.0	212	eMol20378767	−7.9	261	eMol4555647	−7.8
164	CHEMBL1601398	−8.0	213	eMol25908513	−7.9	262	eMol45622743	−7.8
165	eMol2507718	−8.0	214	eMol26309353	−7.9	263	eMol4581546	−7.8
166	eMol26242046	−8.0	215	CHEMBL2259943	−7.9	264	CHEMBL2403015	−7.8
167	eMol36912834	−8.0	216	CHEMBL3818035	−7.9	265	eMol20342220	−7.8
168	eMol580072	−8.0	217	eMol27091442	−7.9	266	eMol24520354	−7.8
169	eMol28321864	−8.0	218	eMol56752349	−7.9	267	eMol27202992	−7.8
170	eMol579718	−8.0	219	CHEMBL3819481	−7.9	268	eMol29638777	−7.8
171	CHEMBL1215578	−8.0	220	CHEMBL462179	−7.9	269	eMol45622749	−7.8
172	eMol1755402	−8.0	221	eMol179164959	−7.9	270	CHEMBL1214861	−7.8
173	eMol18551342	−8.0	222	eMol206910852	−7.9	271	eMol25813417	−7.8
174	CHEMBL3818732	−8.0	223	eMol25810001	−7.9	272	eMol208103615	−7.8
175	eMol206992517	−8.0	224	eMol25810077	−7.9	273	eMol29565259	−7.8
176	eMol256911679	−8.0	225	eMol27202994	−7.9	274	eMol45585838	−7.8
177	eMol27202252	−8.0	226	eMol43906047	−7.9	275	eMol561887	−7.8
178	eMol301527162	−8.0	227	eMol43906063	−7.9	276	eMol23202652	−7.8
179	eMol579756	−8.0	228	eMol679550	−7.9	277	eMol26242026	−7.8
180	eMol679664	−8.0	229	CHEMBL3582080	−7.9	278	eMol26242040	−7.8
181	CHEMBL2259944	−8.0	230	eMol177199339	−7.9	280	eMol4555657	−7.8
182	CHEMBL485839	−8.0	231	eMol20342262	−7.9	281	eMol4572924	−7.8
183	eMol27202242	−8.0	232	eMol206911267	−7.9	282	CHEMBL3819187	−7.8
184	eMol4552519	−8.0	233	eMol300994251	−7.9	283	eMol4389171	−7.8
185	eMol25810089	−8.0	234	eMol46480254	−7.9	279	eMol4740224	−7.8
186	eMol4581322	−8.0	235	eMol48768479	−7.9	284	eMol30646140	−7.7
187	eMol679670	−8.0	236	eMol566380	−7.9	285	eMol4580802	−7.7
188	eMol679824	−8.0	237	eMol581308	−7.9	286	eMol58034831	−7.7
189	CHEMBL1215081	−7.9	238	eMol681814	−7.9	287	eMol72993476	−7.7
190	eMol24389393	−7.9	239	CHEMBL2259942	−7.8	288	eMol1748795	−7.7
191	eMol25966646	−7.9	240	CHEMBL3819186	−7.8	289	eMol179165002	−7.7
192	eMol26196328	−7.9	241	eMol179164947	−7.8	290	eMol206985996	−7.7
193	eMol26242022	−7.9	242	eMol19267414	−7.8	291	eMol26310961	−7.7
194	eMol45585844	−7.9	243	eMol26251042	−7.8	292	eMol27202264	−7.7
195	eMol4566756	−7.9	244	eMol27380659	−7.8	293	eMol4446697	−7.7

Table S1. *Continued.*

No.	Compound Name/Code	Docking Score (kcal/mol)	No.	Compound Name/Code	Docking Score (kcal/mol)	No.	Compound Name/Code	Docking Score (kcal/mol)
294	eMol4579587	−7.7	346	eMol25810163	−7.6	398	eMol26312078	−7.5
295	eMol25909178	−7.7	347	eMol27202262	−7.6	399	eMol26405201	−7.5
296	eMol26311238	−7.7	348	eMol27202268	−7.6	400	eMol45935562	−7.5
297	eMol300445092	−7.7	349	eMol7635480	−7.6	401	eMol46400681	−7.5
298	eMol30665797	−7.7	350	eMol935864	−7.6	402	eMol24116475	−7.5
299	CHEMBL1215004	−7.7	351	CHEMBL3819338	−7.6	403	eMol25896479	−7.5
300	CHEMBL3817850	−7.7	352	CHEMBL1215803	−7.6	404	eMol4587779	−7.5
301	eMol179165014	−7.7	353	CHEMBL485838	−7.6	405	eMol679668	−7.5
302	eMol19076364	−7.7	354	eMol26327269	−7.6	406	CHEMBL1584455	−7.5
303	eMol25965034	−7.7	355	eMol588102	−7.6	407	eMol26314331	−7.5
304	eMol29587306	−7.7	356	eMol8267101	−7.6	408	eMol4386746	−7.5
305	eMol4116278	−7.7	357	eMol26242048	−7.6	409	eMol4480890	−7.5
306	eMol43731202	−7.7	358	eMol3060078	−7.6	410	eMol45935466	−7.5
307	eMol45585836	−7.7	359	eMol46480052	−7.6	411	eMol19266607	−7.5
308	eMol46480194	−7.7	360	eMol956409	−7.6	412	eMol206908008	−7.5
309	eMol679666	−7.7	361	eMol29634797	−7.6	413	eMol26255667	−7.5
310	eMol681860	−7.7	362	eMol4574833	−7.6	414	eMol300014864	−7.5
311	CHEMBL1371074	−7.7	363	eMol46210804	−7.6	415	eMol316351191	−7.5
312	eMol24511821	−7.7	364	eMol46365712	−7.6	416	eMol565754	−7.5
313	eMol26242024	−7.7	365	eMol1675430	−7.6	417	eMol587646	−7.5
314	eMol27202958	−7.7	366	eMol25784115	−7.6	418	CHEMBL3819326	−7.5
315	eMol20342268	−7.7	367	eMol2992488	−7.6	419	CHEMBL602627	−7.5
316	eMol233046940	−7.7	368	eMol46509917	−7.6	420	eMol18940061	−7.5
317	CHEMBL1528362	−7.7	369	eMol944638	−7.6	421	eMol26313085	−7.5
318	eMol19076348	−7.7	370	CHEMBL1214868	−7.6	422	eMol28321866	−7.5
319	eMol24525196	−7.7	371	eMol1764376	−7.6	423	eMol43745241	−7.5
320	eMol26141700	−7.7	372	eMol18958642	−7.6	424	eMol4567258	−7.5
321	eMol26311024	−7.7	373	eMol20377433	−7.6	425	eMol4580053	−7.5
322	eMol27202742	−7.7	374	eMol26683947	−7.6	426	eMol46528447	−7.5
323	eMol4413761	−7.7	375	eMol3318312	−7.6	427	eMol49384601	−7.5
324	CHEMBL517897	−7.7	376	eMol4577787	−7.6	428	eMol50556474	−7.5
325	eMol20342205	−7.7	377	eMol5779489	−7.6	429	eMol566256	−7.5
326	eMol23249723	−7.7	378	CHEMBL520033	−7.5	430	eMol1040103	−7.5
327	eMol43744848	−7.7	379	eMol17203477	−7.5	431	eMol20337444	−7.5
328	eMol48982653	−7.7	380	eMol18860491	−7.5	432	eMol206985560	−7.5
329	CHEMBL3805841	−7.7	381	eMol193447787	−7.5	433	eMol25816251	−7.5
330	CHEMBL3817906	−7.7	382	CHEMBL3817957	−7.5	434	eMol27187406	−7.5
331	eMol25955419	−7.7	383	CHEMBL3818012	−7.5	435	eMol3535618	−7.5
332	eMol562493	−7.7	384	eMol16374637	−7.5	436	eMol4551561	−7.5
333	eMol588242	−7.7	385	eMol24029848	−7.5	437	eMol46479990	−7.5
334	eMol7667302	−7.7	386	eMol3427847	−7.5	438	eMol566118	−7.5
335	eMol26242042	−7.6	387	eMol43906067	−7.5	439	eMol588256	−7.5
336	eMol43731476	−7.6	388	eMol4448933	−7.5	440	eMol6753441	−7.5
337	eMol588388	−7.6	389	eMol51435800	−7.5	441	CHEMBL114364	−7.5
338	CHEMBL323866	−7.6	390	eMol11690694	−7.5	442	CHEMBL250917	−7.5
339	eMol2992508	−7.6	391	eMol16683412	−7.5	443	CHEMBL3818515	−7.5
340	eMol43757885	−7.6	392	eMol27202960	−7.5	444	eMol1429358	−7.5
341	eMol7265152	−7.6	393	eMol45944068	−7.5	445	eMol1679768	−7.5
342	CHEMBL3819140	−7.6	394	eMol935130	−7.5	446	eMol2208925	−7.5
343	eMol3103759	−7.6	395	eMol1751766	−7.5	447	eMol23250559	−7.5
344	eMol32369766	−7.6	396	eMol206987969	−7.5	441	CHEMBL114364	−7.5
345	eMol4726858	−7.6	397	eMol233088901	−7.5	442	CHEMBL250917	−7.5

Table S1. *Continued.*

No.	Compound Name/Code	Docking Score (kcal/mol)	No.	Compound Name/Code	Docking Score (kcal/mol)	No.	Compound Name/Code	Docking Score (kcal/mol)
448	eMol25810127	−7.5	500	eMol26242054	−7.4	552	eMol25901925	−7.3
449	eMol27202290	−7.5	501	eMol26316348	−7.4	553	eMol26245604	−7.3
450	eMol45701494	−7.5	502	eMol27202980	−7.4	554	eMol26259383	−7.3
451	eMol23247039	−7.4	503	eMol43906057	−7.4	555	eMol26324339	−7.3
452	eMol24493983	−7.4	504	eMol46479912	−7.4	556	eMol299991997	−7.3
453	eMol24520756	−7.4	505	eMol46479980	−7.4	557	eMol4075283	−7.3
454	eMol25965334	−7.4	506	CHEMBL3818704	−7.4	558	eMol44673597	−7.3
455	eMol26320021	−7.4	507	eMol26242036	−7.4	559	eMol4485131	−7.3
456	eMol37002179	−7.4	508	eMol565478	−7.4	560	eMol4708971	−7.3
457	eMol4388132	−7.4	509	CHEMBL1402619	−7.4	561	eMol561655	−7.3
458	eMol46480026	−7.4	510	CHEMBL3805023	−7.4	562	eMol956477	−7.3
459	eMol579044	−7.4	511	eMol1025907	−7.4	563	CHEMBL3818910	−7.3
460	eMol587808	−7.4	512	eMol16351683	−7.4	564	eMol207355370	−7.3
461	eMol588272	−7.4	513	eMol206875270	−7.4	565	eMol24520050	−7.3
462	CHEMBL117642	−7.4	514	eMol25810171	−7.4	566	eMol25918241	−7.3
463	CHEMBL1994876	−7.4	515	eMol25937897	−7.4	567	eMol26242052	−7.3
464	eMol27202402	−7.4	516	eMol26311934	−7.4	568	eMol26300049	−7.3
465	eMol3018072	−7.4	517	eMol26936203	−7.4	569	eMol4578697	−7.3
466	eMol46210806	−7.4	518	eMol27202936	−7.4	570	eMol4715267	−7.3
467	eMol75932399	−7.4	519	eMol27202948	−7.4	571	CHEMBL455639	−7.3
468	CHEMBL1499977	−7.4	520	eMol299962831	−7.4	572	eMol1040791	−7.3
469	eMol20354197	−7.4	521	eMol566142	−7.4	573	eMol1678605	−7.3
470	eMol20378545	−7.4	522	CHEMBL1360793	−7.4	574	eMol27091590	−7.3
471	eMol24427880	−7.4	523	CHEMBL1988177	−7.4	575	eMol27115486	−7.3
472	eMol24428326	−7.4	524	CHEMBL3805378	−7.4	576	eMol316344188	−7.3
473	eMol26356948	−7.4	525	CHEMBL511010	−7.4	577	CHEMBL3582082	−7.3
474	eMol27202250	−7.4	526	eMol20356176	−7.4	578	eMol1149947	−7.3
475	eMol27203000	−7.4	527	eMol30017880	−7.4	579	eMol179165005	−7.3
476	eMol2993022	−7.4	528	eMol4934666	−7.4	580	eMol180102976	−7.3
477	eMol46479157	−7.4	529	eMol16352353	−7.4	581	eMol20375154	−7.3
478	eMol24058094	−7.4	530	eMol179177638	−7.4	582	eMol26262902	−7.3
479	eMol24524408	−7.4	531	eMol19076404	−7.4	583	eMol26314721	−7.3
480	eMol26189153	−7.4	532	eMol25902329	−7.4	584	eMol46370139	−7.3
481	eMol27202902	−7.4	533	eMol45928112	−7.4	585	eMol565838	−7.3
482	eMol27338418	−7.4	534	eMol4698359	−7.4	586	CHEMBL116634	−7.3
483	eMol44127206	−7.4	535	eMol4739467	−7.4	587	eMol19076688	−7.3
484	eMol4521223	−7.4	536	CHEMBL1214645	−7.3	588	eMol206964256	−7.3
485	eMol181128729	−7.4	537	CHEMBL1215006	−7.3	589	eMol23191317	−7.3
486	eMol26246365	−7.4	538	eMol1683032	−7.3	590	eMol25902283	−7.3
487	eMol26327656	−7.4	539	eMol24538139	−7.3	591	eMol26230266	−7.3
488	eMol32812942	−7.4	540	eMol26250892	−7.3	592	eMol26315986	−7.3
489	eMol46411087	−7.4	541	eMol276247952	−7.3	593	eMol276247970	−7.3
490	eMol4725672	−7.4	542	eMol299966748	−7.3	594	eMol300990951	−7.3
491	eMol581076	−7.4	543	eMol300978663	−7.3	595	eMol31358919	−7.3
492	eMol956403	−7.4	544	eMol4090808	−7.3	596	eMol43745829	−7.3
493	eMol9919488	−7.4	545	eMol4566508	−7.3	597	eMol1764190	−7.3
494	CHEMBL1215082	−7.4	546	eMol4713633	−7.3	598	eMol207259512	−7.3
495	CHEMBL3819363	−7.4	547	eMol4732807	−7.3	599	eMol23206884	−7.3
496	CHEMBL3819532	−7.4	548	CHEMBL397391	−7.3	600	eMol23242679	−7.3
497	CHEMBL82690	−7.4	549	eMol1324430	−7.3	601	eMol2385157	−7.3
498	eMol25903079	−7.4	550	eMol206850549	−7.3	602	eMol24520802	−7.3
499	eMol25903125	−7.4	551	eMol24526444	−7.3	603	eMol24532024	−7.3

Table S1. *Continued.*

No.	Compound Name/Code	Docking Score (kcal/mol)	No.	Compound Name/Code	Docking Score (kcal/mol)	No.	Compound Name/Code	Docking Score (kcal/mol)
604	eMol25797977	−7.3	654	eMol316831112	−7.2	704	CHEMBL3806097	−7.2
605	eMol27202984	−7.3	655	eMol35699388	−7.2	705	CHEMBL577676	−7.2
606	eMol27202996	−7.3	656	eMol43730464	−7.2	706	CHEMBL585981	−7.2
607	eMol27252901	−7.3	657	eMol46479958	−7.2	707	eMol18516265	−7.2
608	eMol35717814	−7.3	658	eMol935636	−7.2	708	eMol18550720	−7.2
609	eMol43745056	−7.3	659	CHEMBL296691	−7.2	709	eMol20337448	−7.2
610	eMol4549830	−7.3	660	eMol179164953	−7.2	710	eMol2409922	−7.2
611	eMol588158	−7.3	661	eMol23208796	−7.2	711	eMol24521662	−7.2
612	eMol684240	−7.3	662	eMol23241510	−7.2	712	eMol25825125	−7.2
613	eMol956435	−7.3	663	eMol24521330	−7.2	713	eMol27202756	−7.2
614	CHEMBL115809	−7.3	664	eMol25770408	−7.2	714	eMol32813234	−7.2
615	eMol1429356	−7.3	665	eMol25902291	−7.2	715	eMol4433627	−7.2
616	eMol19076598	−7.3	666	eMol25968613	−7.2	716	eMol936632	−7.2
617	eMol19076750	−7.3	667	eMol26219237	−7.2	717	eMol2231344	−7.2
618	eMol206907968	−7.3	668	eMol4726520	−7.2	718	eMol23201309	−7.2
619	eMol25907499	−7.3	669	eMol5847921	−7.2	719	eMol26300005	−7.2
620	eMol25913211	−7.3	670	eMol588228	−7.2	720	eMol27202506	−7.2
621	eMol25936279	−7.3	671	eMol935898	−7.2	721	eMol27202920	−7.2
622	eMol27202388	−7.3	672	CHEMBL3817866	−7.2	722	eMol31760166	−7.2
623	eMol300154219	−7.3	673	CHEMBL4099863	−7.2	723	eMol43746869	−7.2
624	eMol4477952	−7.3	674	CHEMBL47157	−7.2	724	eMol4408548	−7.2
625	eMol579714	−7.3	675	CHEMBL579620	−7.2	725	eMol4706083	−7.2
626	eMol581304	−7.3	676	eMol16056893	−7.2	726	eMol565918	−7.2
627	CHEMBL116408	−7.3	677	eMol17192354	−7.2	727	CHEMBL3806178	−7.2
628	eMol2330950	−7.3	678	eMol180102764	−7.2	728	eMol18551352	−7.2
629	eMol25895023	−7.3	679	eMol19076602	−7.2	729	eMol2130046	−7.2
630	eMol25966304	−7.3	680	eMol26285666	−7.2	730	eMol23170772	−7.2
631	eMol26285150	−7.3	681	eMol26289047	−7.2	731	eMol24518463	−7.2
632	eMol26326968	−7.3	682	eMol26314361	−7.2	732	eMol25958221	−7.2
633	eMol27202912	−7.3	683	eMol26569999	−7.2	733	eMol26277244	−7.2
634	eMol27202970	−7.3	684	eMol26936207	−7.2	734	eMol26292416	−7.2
635	eMol2880336	−7.3	685	eMol27202540	−7.2	735	eMol26310907	−7.2
636	eMol30744925	−7.3	686	eMol2768990	−7.2	736	eMol26316052	−7.2
637	eMol43731530	−7.3	687	eMol4606566	−7.2	737	eMol26328784	−7.2
638	eMol46400679	−7.3	688	eMol46370137	−7.2	738	eMol27202410	−7.2
639	eMol4728946	−7.3	689	eMol46400677	−7.2	739	eMol27202762	−7.2
640	eMol8238281	−7.3	690	eMol46491554	−7.2	740	eMol32813226	−7.2
641	CHEMBL3818933	−7.3	691	eMol4713307	−7.2	741	eMol33346435	−7.2
642	eMol11071566	−7.3	692	CHEMBL117438	−7.2	742	eMol4490859	−7.2
643	eMol24521048	−7.3	693	CHEMBL3818229	−7.2	743	eMol46445765	−7.2
644	eMol25771182	−7.3	694	eMol22370884	−7.2	744	eMol46479996	−7.2
645	eMol29649914	−7.3	695	eMol27045407	−7.2	745	CHEMBL326628	−7.2
646	eMol45622753	−7.3	696	eMol27202398	−7.2	746	CHEMBL4079984	−7.2
647	eMol588168	−7.3	697	eMol27202754	−7.2	747	eMol1120546	−7.2
648	CHEMBL116817	−7.2	698	eMol27202914	−7.2	748	eMol181109768	−7.2
649	CHEMBL1632342	−7.2	699	eMol27202918	−7.2	749	eMol27202350	−7.2
650	eMol179164956	−7.2	700	eMol32246523	−7.2	750	eMol43731774	−7.2
651	eMol24419686	−7.2	701	eMol4709495	−7.2	751	eMol45133343	−7.2
652	eMol26285928	−7.2	702	eMol4718679	−7.2	752	eMol46400687	−7.2
653	eMol27202916	−7.2	703	eMol5444969	−7.2	753	eMol566406	−7.2

Table S1. *Continued.*

No.	Compound Name/Code	Docking Score (kcal/mol)	No.	Compound Name/Code	Docking Score (kcal/mol)	No.	Compound Name/Code	Docking Score (kcal/mol)
754	eMol679820	−7.2	806	eMol587916	−7.1	858	eMol27187414	−7.1
755	CHEMBL3805042	−7.2	807	CHEMBL34185	−7.1	859	eMol27202282	−7.1
756	CHEMBL3819311	−7.2	808	CHEMBL3804857	−7.1	860	eMol30104228	−7.1
757	eMol206921779	−7.2	809	CHEMBL3818747	−7.1	861	eMol5444645	−7.1
758	eMol2141753	−7.2	810	CHEMBL38330	−7.1	862	eMol5444883	−7.1
759	eMol23246986	−7.2	811	eMol20337442	−7.1	863	eMol588078	−7.1
760	eMol24113359	−7.2	812	eMol20380394	−7.1	864	CHEMBL3818856	−7.1
761	eMol24428240	−7.2	813	eMol23185851	−7.1	865	eMol18542840	−7.1
762	eMol24520240	−7.2	814	eMol25770418	−7.1	866	eMol24428006	−7.1
763	eMol25810099	−7.2	815	eMol25902049	−7.1	867	eMol2517109	−7.1
764	eMol25829045	−7.2	816	eMol26319543	−7.1	868	eMol25819233	−7.1
765	eMol27045405	−7.2	817	eMol26936201	−7.1	869	eMol25903659	−7.1
766	eMol27045521	−7.2	818	eMol3033983	−7.1	870	eMol25945602	−7.1
767	eMol30322739	−7.2	819	eMol6263477	−7.1	871	eMol25966294	−7.1
768	eMol44548966	−7.2	820	CHEMBL114005	−7.1	872	eMol26189151	−7.1
769	eMol45708568	−7.2	821	CHEMBL3805407	−7.1	873	eMol26312838	−7.1
770	eMol4738130	−7.2	822	eMol16351675	−7.1	874	eMol26314117	−7.1
771	eMol50301465	−7.2	823	eMol24521248	−7.1	875	eMol27202280	−7.1
772	eMol1156520	−7.2	824	eMol26264151	−7.1	876	eMol27202380	−7.1
773	eMol18550714	−7.2	825	eMol26276502	−7.1	877	eMol29665906	−7.1
774	eMol18961180	−7.2	826	eMol26313449	−7.1	878	eMol32812998	−7.1
775	eMol23241342	−7.2	827	eMol26331720	−7.1	879	eMol33339294	−7.1
776	eMol24519079	−7.2	828	eMol27202362	−7.1	880	eMol4572232	−7.1
777	eMol25963663	−7.2	829	eMol33346433	−7.1	881	eMol46545587	−7.1
778	eMol26289870	−7.2	830	eMol43731198	−7.1	882	eMol48597407	−7.1
779	eMol26330383	−7.2	831	eMol45935468	−7.1	883	eMol579510	−7.1
780	eMol27202358	−7.2	832	eMol46509913	−7.1	884	eMol588368	−7.1
781	eMol3317346	−7.2	833	eMol4732869	−7.1	885	CHEMBL1215796	−7.1
782	CHEMBL1302012	−7.1	834	CHEMBL113923	−7.1	886	CHEMBL1591720	−7.1
783	CHEMBL3806218	−7.1	835	CHEMBL1383006	−7.1	887	CHEMBL3818171	−7.1
784	CHEMBL3818209	−7.1	836	CHEMBL1559857	−7.1	888	CHEMBL395117	−7.1
785	CHEMBL3818582	−7.1	837	CHEMBL3805665	−7.1	889	eMol1165997	−7.1
786	eMol18550716	−7.1	838	eMol16351673	−7.1	890	eMol20310561	−7.1
787	eMol206980481	−7.1	839	eMol19076826	−7.1	891	eMol20378615	−7.1
788	eMol26250339	−7.1	840	eMol20380392	−7.1	892	eMol206920578	−7.1
789	eMol26310839	−7.1	841	eMol25810081	−7.1	893	eMol206965980	−7.1
790	eMol43731776	−7.1	842	eMol26251302	−7.1	894	eMol2194316	−7.1
791	eMol46534208	−7.1	843	eMol26270766	−7.1	895	eMol2408165	−7.1
792	eMol5672543	−7.1	844	eMol26276632	−7.1	896	eMol24520553	−7.1
793	CHEMBL1414265	−7.1	845	eMol26314895	−7.1	897	eMol25790309	−7.1
794	CHEMBL247353	−7.1	846	eMol26936209	−7.1	898	eMol25913057	−7.1
795	eMol16351883	−7.1	847	eMol27187400	−7.1	899	eMol26314359	−7.1
796	eMol16375743	−7.1	848	eMol4146706	−7.1	900	eMol26315688	−7.1
797	eMol17202599	−7.1	849	eMol45932769	−7.1	901	eMol27202336	−7.1
798	eMol18866942	−7.1	850	eMol936420	−7.1	902	eMol27202496	−7.1
799	eMol20344945	−7.1	851	CHEMBL1551922	−7.1	903	eMol27202778	−7.1
800	eMol206964558	−7.1	852	CHEMBL3818977	−7.1	904	eMol300089978	−7.1
801	eMol2115166	−7.1	853	eMol206990555	−7.1	905	eMol3181655	−7.1
802	eMol23242609	−7.1	854	eMol2166708	−7.1	906	eMol579926	−7.1
803	eMol26292060	−7.1	855	eMol24524699	−7.1	907	CHEMBL1439596	−7.1
804	eMol36016142	−7.1	856	eMol25771206	−7.1	908	CHEMBL1562035	−7.1
805	eMol560303	−7.1	857	eMol26286396	−7.1	909	CHEMBL3818499	−7.1

Table S1. *Continued.*

No.	Compound Name/Code	Docking Score (kcal/mol)	No.	Compound Name/Code	Docking Score (kcal/mol)	No.	Compound Name/Code	Docking Score (kcal/mol)
910	CHEMBL3819196	−7.1	961	eMol2308510	−7.0	1012	CHEMBL3818158	−7.0
911	CHEMBL550397	−7.1	962	eMol25901881	−7.0	1013	eMol18550710	−7.0
912	eMol1035551	−7.1	963	eMol26263266	−7.0	1014	eMol24430685	−7.0
913	eMol18550722	−7.1	964	eMol26315041	−7.0	1015	eMol2513652	−7.0
914	eMol206849669	−7.1	965	eMol26524217	−7.0	1016	eMol25770414	−7.0
915	eMol24430231	−7.1	966	eMol46400683	−7.0	1017	eMol25931375	−7.0
916	eMol24531722	−7.1	967	eMol4709315	−7.0	1018	eMol26141682	−7.0
917	eMol24531798	−7.1	968	eMol72578631	−7.0	1019	eMol26309887	−7.0
918	eMol25912447	−7.1	969	CHEMBL2297150	−7.0	1020	eMol26313748	−7.0
919	eMol26288955	−7.1	970	CHEMBL3818347	−7.0	1021	eMol26762439	−7.0
920	eMol26326313	−7.1	971	CHEMBL3818366	−7.0	1022	eMol27202208	−7.0
921	eMol26350850	−7.1	972	eMol16379841	−7.0	1023	eMol36015268	−7.0
922	eMol27187440	−7.1	973	eMol19076734	−7.0	1024	eMol566554	−7.0
923	eMol299994300	−7.1	974	eMol19076828	−7.0	1025	ChEBI114797	−7.0
924	eMol3021959	−7.1	975	eMol20356657	−7.0	1026	CHEMBL1452417	−7.0
925	eMol31278496	−7.1	976	eMol23152351	−7.0	1027	CHEMBL1967636	−7.0
926	eMol36016132	−7.1	977	eMol25816255	−7.0	1028	CHEMBL3627816	−7.0
927	eMol4491662	−7.1	978	eMol25895009	−7.0	1029	CHEMBL38232	−7.0
928	eMol956263	−7.1	979	eMol26310653	−7.0	1030	CHEMBL90574	−7.0
929	CHEMBL1423402	−7.1	980	eMol26314551	−7.0	1031	eMol16351609	−7.0
930	CHEMBL1552848	−7.1	981	eMol27202320	−7.0	1032	eMol206978896	−7.0
931	CHEMBL299107	−7.1	982	eMol29987858	−7.0	1033	eMol206996167	−7.0
932	CHEMBL3804866	−7.1	983	eMol3775722	−7.0	1034	eMol2232474	−7.0
933	eMol106491702	−7.1	984	eMol4565043	−7.0	1035	eMol23237618	−7.0
934	eMol16351677	−7.1	985	eMol565850	−7.0	1036	eMol24520280	−7.0
935	eMol206909136	−7.1	986	eMol6157528	−7.0	1037	eMol25827835	−7.0
936	eMol23244410	−7.1	987	CHEMBL1214934	−7.0	1038	eMol25896473	−7.0
937	eMol24520023	−7.1	988	CHEMBL1900041	−7.0	1039	eMol26262940	−7.0
938	eMol24526765	−7.1	989	CHEMBL2297667	−7.0	1040	eMol26349365	−7.0
939	eMol25784531	−7.1	990	CHEMBL3818626	−7.0	1041	eMol26355834	−7.0
940	eMol25913175	−7.1	991	eMol15983174	−7.0	1042	eMol26581878	−7.0
941	eMol25924517	−7.1	992	eMol19076732	−7.0	1043	eMol44057517	−7.0
942	eMol26293580	−7.1	993	eMol206965358	−7.0	1044	eMol4719207	−7.0
943	eMol26570001	−7.1	994	eMol206987841	−7.0	1045	eMol48554523	−7.0
944	eMol27202232	−7.1	995	eMol24427986	−7.0	1046	eMol560313	−7.0
945	eMol27202422	−7.1	996	eMol24519100	−7.0	1047	eMol566074	−7.0
946	eMol316862506	−7.1	997	eMol25895011	−7.0	1048	eMol784256	−7.0
947	eMol36904288	−7.1	998	eMol26262168	−7.0	1049	CHEMBL1357492	−7.0
948	eMol43744846	−7.1	999	eMol26299531	−7.0	1050	eMol1324432	−7.0
949	eMol679754	−7.1	1000	eMol26324447	−7.0	1051	eMol206979384	−7.0
950	CHEMBL1373990	−7.0	1001	eMol27202224	−7.0	1052	eMol2194269	−7.0
951	CHEMBL1394809	−7.0	1002	eMol27202288	−7.0	1053	eMol24548037	−7.0
952	CHEMBL3804880	−7.0	1003	eMol27202520	−7.0	1054	eMol25903928	−7.0
953	CHEMBL3819459	−7.0	1004	eMol276260499	−7.0	1055	eMol25964562	−7.0
954	eMol1149869	−7.0	1005	eMol29262171	−7.0	1056	eMol2597848	−7.0
955	eMol16352193	−7.0	1006	eMol36016032	−7.0	1057	eMol26237970	−7.0
956	eMol17203597	−7.0	1007	eMol4708889	−7.0	1058	eMol26317983	−7.0
957	eMol1752196	−7.0	1008	eMol5779704	−7.0	1059	eMol26775365	−7.0
958	eMol19076370	−7.0	1009	CHEMBL1553991	−7.0	1060	eMol27187482	−7.0
959	eMol206843436	−7.0	1010	CHEMBL1882658	−7.0	1061	eMol30132606	−7.0
960	eMol206964210	−7.0	1011	CHEMBL3805512	−7.0	1062	eMol3138656	−7.0

Table S1. *Continued.*

No.	Compound Name/Code	Docking Score (kcal/mol)	No.	Compound Name/Code	Docking Score (kcal/mol)	No.	Compound Name/Code	Docking Score (kcal/mol)
1063	eMol36015282	−7.0	1114	eMol206904349	−7.0	1165	eMol300979175	−6.9
1064	eMol46400685	−7.0	1115	eMol24512023	−7.0	1166	eMol36015346	−6.9
1065	eMol46525150	−7.0	1116	eMol25927095	−7.0	1167	eMol36015494	−6.9
1066	eMol4744993	−7.0	1117	eMol27045527	−7.0	1168	eMol43678312	−6.9
1067	eMol49849265	−7.0	1118	eMol27202360	−7.0	1169	eMol46544815	−6.9
1068	eMol685328	−7.0	1119	eMol27202532	−7.0	1170	eMol11867096	−6.9
1069	CHEMBL3804836	−7.0	1120	eMol27202896	−7.0	1171	eMol17203549	−6.9
1070	CHEMBL3818974	−7.0	1121	eMol27435660	−7.0	1172	eMol206964418	−6.9
1071	CHEMBL3819285	−7.0	1122	eMol43749117	−7.0	1173	eMol206969744	−6.9
1072	CHEMBL46581	−7.0	1123	eMol4475588	−7.0	1174	eMol206972648	−6.9
1073	eMol18542077	−7.0	1124	eMol45621707	−7.0	1175	eMol206987102	−6.9
1074	eMol19031814	−7.0	1125	eMol4712268	−7.0	1176	eMol22299802	−6.9
1075	eMol24430273	−7.0	1126	eMol4745700	−7.0	1177	eMol24519081	−6.9
1076	eMol25902841	−7.0	1127	CHEMBL1451971	−7.0	1178	eMol24546968	−6.9
1077	eMol26263652	−7.0	1128	CHEMBL18944	−7.0	1179	eMol25810157	−6.9
1078	eMol27202894	−7.0	1129	CHEMBL3805136	−7.0	1180	eMol25813419	−6.9
1079	eMol30104216	−7.0	1130	CHEMBL3819605	−7.0	1181	eMol25913361	−6.9
1080	eMol3091619	−7.0	1131	eMol14069413	−7.0	1182	eMol26141662	−6.9
1081	eMol5538242	−7.0	1132	eMol20352703	−7.0	1183	eMol26249494	−6.9
1082	eMol565910	−7.0	1133	eMol23239776	−7.0	1184	eMol26256475	−6.9
1083	eMol935992	−7.0	1134	eMol25896475	−7.0	1185	eMol26264167	−6.9
1084	eMol9989242	−7.0	1135	eMol25902133	−7.0	1186	eMol26300497	−6.9
1085	CHEMBL1303106	−7.0	1136	eMol25929538	−7.0	1187	eMol26310555	−6.9
1086	eMol1170739	−7.0	1137	eMol25963073	−7.0	1188	eMol27202884	−6.9
1087	eMol19076366	−7.0	1138	eMol25968661	−7.0	1189	eMol276247954	−6.9
1088	eMol206875356	−7.0	1139	eMol26141678	−7.0	1190	eMol300978919	−6.9
1089	eMol24008012	−7.0	1140	eMol26189265	−7.0	1191	eMol35693189	−6.9
1090	eMol25954676	−7.0	1141	eMol26295757	−7.0	1192	eMol36898082	−6.9
1091	eMol25961619	−7.0	1142	eMol26313219	−7.0	1193	eMol679750	−6.9
1092	eMol26245266	−7.0	1143	eMol26327169	−7.0	1194	eMol72964183	−6.9
1093	eMol26290986	−7.0	1144	eMol27202900	−7.0	1195	eMol935934	−6.9
1094	eMol26312834	−7.0	1145	eMol276247964	−7.0	1196	CHEMBL1867264	−6.9
1095	eMol26314441	−7.0	1146	eMol300206844	−7.0	1197	CHEMBL2297819	−6.9
1096	eMol26398726	−7.0	1147	eMol300261457	−7.0	1198	CHEMBL3818554	−6.9
1097	eMol27202758	−7.0	1148	eMol30083452	−7.0	1199	eMol16374969	−6.9
1098	eMol300021201	−7.0	1149	eMol36015616	−7.0	1200	eMol206963343	−6.9
1099	eMol300273851	−7.0	1150	eMol560309	−7.0	1201	eMol207355368	−6.9
1100	eMol36015642	−7.0	1151	eMol15924858	−6.9	1202	eMol23246252	−6.9
1101	eMol45787893	−7.0	1152	eMol1683469	−6.9	1203	eMol25902478	−6.9
1102	eMol4735810	−7.0	1153	eMol18551390	−6.9	1204	eMol27202274	−6.9
1103	eMol561781	−7.0	1154	eMol19076694	−6.9	1205	eMol27202906	−6.9
1104	eMol561985	−7.0	1155	eMol20368452	−6.9	1206	eMol277677808	−6.9
1105	eMol6777617	−7.0	1156	eMol206910454	−6.9	1207	eMol2981967	−6.9
1106	CHEMBL1554351	−7.0	1157	eMol26262090	−6.9	1208	eMol4713349	−6.9
1107	CHEMBL3806234	−7.0	1158	eMol26310743	−6.9	1209	CHEMBL1405217	−6.9
1108	CHEMBL3819289	−7.0	1159	eMol26313445	−6.9	1210	CHEMBL1437240	−6.9
1109	CHEMBL4091026	−7.0	1160	eMol27045535	−6.9	1211	CHEMBL3818631	−6.9
1110	eMol15983252	−7.0	1161	eMol27202324	−6.9	1212	eMol17205607	−6.9
1111	eMol16390197	−7.0	1162	eMol27202744	−6.9	1213	eMol20379434	−6.9
1112	eMol18588453	−7.0	1163	eMol276247956	−6.9	1214	eMol206846147	−6.9
1113	eMol19076492	−7.0	1164	eMol2987076	−6.9	1215	eMol206907645	−6.9

Table S1. *Continued.*

No.	Compound Name/Code	Docking Score (kcal/mol)	No.	Compound Name/Code	Docking Score (kcal/mol)	No.	Compound Name/Code	Docking Score (kcal/mol)
1216	eMol23166688	−6.9	1267	eMol25962247	−6.9	1323	eMol2124007	−6.9
1217	eMol24430221	−6.9	1268	eMol26253980	−6.9	1324	eMol2192224	−6.9
1218	eMol24518467	−6.9	1269	eMol27202334	−6.9	1325	eMol24100760	−6.9
1219	eMol24520575	−6.9	1270	eMol27202782	−6.9	1326	eMol25813397	−6.9
1220	eMol25770140	−6.9	1271	eMol36015106	−6.9	1327	eMol25813443	−6.9
1221	eMol25795727	−6.9	1272	eMol681896	−6.9	1328	eMol25938613	−6.9
1222	eMol2588283	−6.9	1273	CHEMBL1380757	−6.9	1329	eMol26272160	−6.9
1223	eMol26242020	−6.9	1274	CHEMBL1521258	−6.9	1330	eMol27187248	−6.9
1224	eMol26262644	−6.9	1275	CHEMBL1597816	−6.9	1331	eMol27202392	−6.9
1225	eMol26316338	−6.9	1276	eMol1454668	−6.9	1332	eMol27202394	−6.9
1226	eMol26323441	−6.9	1277	eMol20333678	−6.9	1333	eMol27202400	−6.9
1227	eMol26339205	−6.9	1278	eMol206964698	−6.9	1334	eMol27202794	−6.9
1228	eMol27202226	−6.9	1279	eMol23198915	−6.9	1335	eMol27202904	−6.9
1229	eMol3005040	−6.9	1280	eMol24507755	−6.9	1336	eMol27670260	−6.9
1230	eMol32813186	−6.9	1281	eMol24525232	−6.9	1337	eMol2768992	−6.9
1231	eMol36015820	−6.9	1282	eMol25810165	−6.9	1338	eMol31399220	−6.9
1232	eMol44059508	−6.9	1283	eMol25896463	−6.9	1339	eMol46445438	−6.9
1233	eMol4475278	−6.9	1284	eMol25903653	−6.9	1340	CHEMBL1573403	−6.9
1234	eMol4482176	−6.9	1285	eMol26141674	−6.9	1341	CHEMBL1708737	−6.9
1235	eMol46509915	−6.9	1286	eMol26285794	−6.9	1342	CHEMBL3804897	−6.9
1236	eMol4708095	−6.9	1287	eMol26322685	−6.9	1343	CHEMBL3818915	−6.9
1237	CHEMBL1376830	−6.9	1288	eMol314783893	−6.9	1344	eMol1751356	−6.9
1238	CHEMBL1642185	−6.9	1289	eMol316838531	−6.9	1345	eMol20369159	−6.9
1239	CHEMBL3804835	−6.9	1290	eMol3458659	−6.9	1346	eMol206906676	−6.9
1240	eMol177234561	−6.9	1291	eMol4095805	−6.9	1347	eMol206962769	−6.9
1241	eMol18614804	−6.9	1292	eMol43745140	−6.9	1348	eMol24535222	−6.9
1242	eMol18939007	−6.9	1293	eMol45560661	−6.9	1349	eMol2454468	−6.9
1243	eMol19076402	−6.9	1294	eMol46509911	−6.9	1350	eMol25770138	−6.9
1244	eMol206843418	−6.9	1295	eMol4702761	−6.9	1351	eMol25816249	−6.9
1245	eMol24537876	−6.9	1296	eMol4729332	−6.9	1352	eMol25881264	−6.9
1246	eMol25829407	−6.9	1297	CHEMBL1215800	−6.9	1353	eMol26249794	−6.9
1247	eMol26141694	−6.9	1298	CHEMBL3818900	−6.9	1354	eMol27202342	−6.9
1248	eMol26288755	−6.9	1299	eMol1170743	−6.9	1355	eMol2992544	−6.9
1249	eMol26296745	−6.9	1300	eMol20344947	−6.9	1356	eMol36015274	−6.9
1250	eMol27009455	−6.9	1301	eMol206843458	−6.9	1357	eMol36015492	−6.9
1251	eMol27187452	−6.9	1302	eMol24518839	−6.9	1358	eMol43772496	−6.9
1252	eMol27202270	−6.9	1303	eMol25797913	−6.9	1359	eMol935732	−6.9
1253	eMol300120324	−6.9	1304	eMol26276724	−6.9	1360	CHEMBL1215718	−6.8
1254	eMol36016024	−6.9	1305	eMol26312476	−6.9	1361	CHEMBL1473864	−6.8
1255	eMol4729129	−6.9	1306	eMol26336500	−6.9	1362	CHEMBL3805000	−6.8
1256	eMol588436	−6.9	1307	eMol27202204	−6.9	1363	CHEMBL3805183	−6.8
1257	eMol6152648	−6.9	1308	eMol27202490	−6.9	1364	eMol16352261	−6.8
1258	CHEMBL1423559	−6.9	1314	eMol936008	−6.9	1365	eMol1753924	−6.8
1259	CHEMBL1551465	−6.9	1315	eMol97777935	−6.9	1366	eMol19076612	−6.8
1260	CHEMBL3806000	−6.9	1316	CHEMBL1365625	−6.9	1367	eMol206843434	−6.8
1261	CHEMBL3818963	−6.9	1317	CHEMBL1563292	−6.9	1368	eMol206907719	−6.8
1262	CHEMBL3819529	−6.9	1318	CHEMBL3805374	−6.9	1369	eMol2390664	−6.8
1263	eMol16352167	−6.9	1319	CHEMBL3819159	−6.9	1370	eMol25881326	−6.8
1264	eMol19031727	−6.9	1320	CHEMBL46934	−6.9	1371	eMol26196310	−6.8
1265	eMol20368839	−6.9	1321	eMol16352175	−6.9	1372	eMol26313588	−6.8
1266	eMol2087020	−6.9	1322	eMol206843438	−6.9	1373	eMol26353495	−6.8

Table S1. *Continued.*

No.	Compound Name/Code	Docking Score (kcal/mol)	No.	Compound Name/Code	Docking Score (kcal/mol)	No.	Compound Name/Code	Docking Score (kcal/mol)
1374	eMol26773948	−6.8	1425	eMol25915192	−6.8	1476	eMol27186978	−6.8
1375	eMol27187362	−6.8	1426	eMol26132228	−6.8	1477	eMol27202214	−6.8
1376	eMol27202216	−6.8	1427	eMol26275086	−6.8	1478	eMol27202316	−6.8
1377	eMol27202470	−6.8	1428	eMol26312734	−6.8	1479	eMol27202498	−6.8
1378	eMol300151404	−6.8	1429	eMol26313903	−6.8	1480	eMol27202516	−6.8
1379	eMol32246513	−6.8	1430	eMol27202302	−6.8	1481	eMol27203792	−6.8
1380	eMol36015640	−6.8	1431	eMol27202420	−6.8	1482	eMol28321868	−6.8
1381	eMol36016150	−6.8	1432	eMol27202512	−6.8	1483	eMol29955345	−6.8
1382	eMol43745867	−6.8	1433	eMol27202522	−6.8	1484	eMol3416565	−6.8
1383	eMol45585830	−6.8	1434	eMol27202796	−6.8	1485	eMol45560665	−6.8
1384	eMol45948876	−6.8	1435	eMol27202810	−6.8	1486	eMol4559848	−6.8
1385	eMol48957687	−6.8	1436	eMol276221097	−6.8	1487	eMol5444783	−6.8
1386	CHEMBL1574680	−6.8	1437	eMol303143674	−6.8	1488	eMol579900	−6.8
1387	CHEMBL2263322	−6.8	1438	eMol3052109	−6.8	1489	eMol681904	−6.8
1388	CHEMBL2297666	−6.8	1439	eMol4472622	−6.8	1490	eMol99063354	−6.8
1389	CHEMBL3805091	−6.8	1440	eMol4701187	−6.8	1491	CHEMBL1983712	−6.8
1390	CHEMBL3805234	−6.8	1441	eMol565950	−6.8	1492	eMol16351695	−6.8
1391	CHEMBL3806297	−6.8	1442	CHEMBL295884	−6.8	1493	eMol1755604	−6.8
1392	eMol16351581	−6.8	1443	CHEMBL3819359	−6.8	1494	eMol206843432	−6.8
1393	eMol16351857	−6.8	1444	eMol18809799	−6.8	1495	eMol206907070	−6.8
1394	eMol20379714	−6.8	1445	eMol19076600	−6.8	1496	eMol24537526	−6.8
1395	eMol25902666	−6.8	1446	eMol207161823	−6.8	1497	eMol2464575	−6.8
1396	eMol26262986	−6.8	1447	eMol24518650	−6.8	1498	eMol25775761	−6.8
1397	eMol26299003	−6.8	1448	eMol24529894	−6.8	1499	eMol25810093	−6.8
1398	eMol26311192	−6.8	1449	eMol24530962	−6.8	1500	eMol25813423	−6.8
1399	eMol26344402	−6.8	1450	eMol25828609	−6.8	1501	eMol26300679	−6.8
1400	eMol27045529	−6.8	1451	eMol25881336	−6.8	1502	eMol27045531	−6.8
1401	eMol27202284	−6.8	1452	eMol26270390	−6.8	1503	eMol27202354	−6.8
1402	eMol27202390	−6.8	1453	eMol27187286	−6.8	1504	eMol27202508	−6.8
1403	eMol27202472	−6.8	1454	eMol27187438	−6.8	1505	eMol27202526	−6.8
1404	eMol27202488	−6.8	1455	eMol27202298	−6.8	1506	eMol27202774	−6.8
1405	eMol27202518	−6.8	1456	eMol27202344	−6.8	1507	eMol43704541	−6.8
1406	eMol43789923	−6.8	1457	eMol27203752	−6.8	1508	eMol4698777	−6.8
1407	eMol4740690	−6.8	1458	eMol300132718	−6.8	1509	eMol5445037	−6.8
1408	eMol5444735	−6.8	1459	eMol43474659	−6.8	1510	eMol579240	−6.8
1409	eMol588270	−6.8	1460	CHEMBL1472653	−6.8	1511	CHEMBL1317703	−6.8
1410	CHEMBL1214866	−6.8	1461	CHEMBL1474371	−6.8	1512	CHEMBL1376406	−6.8
1411	CHEMBL1510504	−6.8	1462	CHEMBL1902190	−6.8	1513	CHEMBL1501960	−6.8
1412	CHEMBL1716475	−6.8	1463	CHEMBL3417627	−6.8	1514	CHEMBL1502049	−6.8
1413	CHEMBL43701	−6.8	1464	eMol13526748	−6.8	1515	eMol12904258	−6.8
1414	CHEMBL594345	−6.8	1465	eMol17186560	−6.8	1516	eMol1745976	−6.8
1415	eMol1120278	−6.8	1466	eMol18551298	−6.8	1517	eMol20347819	−6.8
1416	eMol1749194	−6.8	1467	eMol19076670	−6.8	1518	eMol20348534	−6.8
1417	eMol1764832	−6.8	1468	eMol206977589	−6.8	1519	eMol20382776	−6.8
1418	eMol20333666	−6.8	1469	eMol206985300	−6.8	1520	eMol206904385	−6.8
1419	eMol20354854	−6.8	1470	eMol2236870	−6.8	1521	eMol206906244	−6.8
1420	eMol206910858	−6.8	1471	eMol23158354	−6.8	1522	eMol24526415	−6.8
1421	eMol23247689	−6.8	1472	eMol24100006	−6.8	1523	eMol24545861	−6.8
1422	eMol24519266	−6.8	1473	eMol24518499	−6.8	1524	eMol25875519	−6.8
1423	eMol24540588	−6.8	1474	eMol26264896	−6.8	1525	eMol26244563	−6.8
1424	eMol24641150	−6.8	1475	eMol27115464	−6.8	1526	eMol26259033	−6.8

Table S1. *Continued.*

No.	Compound Name/Code	Docking Score (kcal/mol)	No.	Compound Name/Code	Docking Score (kcal/mol)	No.	Compound Name/Code	Docking Score (kcal/mol)
1527	eMol26312406	−6.8	1578	CHEMBL1355953	−6.8	1629	eMol43777008	−6.7
1528	eMol26338914	−6.8	1579	CHEMBL4060648	−6.8	1630	eMol4535449	−6.7
1529	eMol27490297	−6.8	1580	eMol16407928	−6.8	1631	eMol49205494	−6.7
1530	eMol36015298	−6.8	1581	eMol18536845	−6.8	1632	eMol5509851	−6.7
1531	eMol43731438	−6.8	1582	eMol19764608	−6.8	1633	CHEMBL1357400	−6.7
1532	eMol5445003	−6.8	1583	eMol232769854	−6.8	1634	CHEMBL1414779	−6.7
1533	eMol561553	−6.8	1584	eMol23983178	−6.8	1635	CHEMBL3806144	−6.7
1534	eMol561867	−6.8	1585	eMol24432101	−6.8	1636	eMol1035523	−6.7
1535	eMol7261106	−6.8	1586	eMol25153702	−6.8	1637	eMol16379399	−6.7
1536	eMol7274676	−6.8	1587	eMol25816253	−6.8	1638	eMol16390191	−6.7
1537	eMol936252	−6.8	1588	eMol25819229	−6.8	1639	eMol16856	−6.7
1538	CHEMBL1399555	−6.8	1589	eMol27045413	−6.8	1640	eMol18532109	−6.7
1539	eMol16379069	−6.8	1590	eMol27045585	−6.8	1641	eMol19076832	−6.7
1540	eMol16379735	−6.8	1591	eMol27202196	−6.8	1642	eMol20354735	−6.7
1541	eMol1676253	−6.8	1592	eMol27202326	−6.8	1643	eMol206843416	−6.7
1542	eMol1751618	−6.8	1593	eMol27202514	−6.8	1644	eMol206847221	−6.7
1543	eMol20382225	−6.8	1594	eMol27202740	−6.8	1645	eMol206992993	−6.7
1544	eMol206977831	−6.8	1595	eMol31340346	−6.8	1646	eMol20732599	−6.7
1545	eMol24427974	−6.8	1596	eMol4418979	−6.8	1647	eMol25782835	−6.7
1546	eMol24530395	−6.8	1597	eMol46526926	−6.8	1648	eMol25784119	−6.7
1547	eMol2480990	−6.8	1598	CHEMBL1214798	−6.7	1649	eMol25817109	−6.7
1548	eMol25813405	−6.8	1599	CHEMBL416260	−6.7	1650	eMol26052954	−6.7
1549	eMol25903601	−6.8	1600	eMol20376367	−6.7	1651	eMol26141632	−6.7
1550	eMol26295370	−6.8	1601	eMol20381008	−6.7	1652	eMol26267262	−6.7
1551	eMol26308952	−6.8	1602	eMol24453856	−6.7	1653	eMol26298841	−6.7
1552	eMol27045571	−6.8	1603	eMol25810169	−6.7	1654	eMol27202908	−6.7
1553	eMol27187238	−6.8	1604	eMol25902941	−6.7	1655	eMol300135787	−6.7
1554	eMol27187318	−6.8	1605	eMol25937625	−6.7	1656	eMol3460427	−6.7
1555	eMol27187472	−6.8	1606	eMol27202266	−6.7	1657	eMol36015846	−6.7
1556	eMol27202764	−6.8	1607	eMol27202510	−6.7	1658	eMol36016030	−6.7
1557	eMol27490289	−6.8	1608	eMol27202780	−6.7	1659	eMol3651760	−6.7
1558	eMol30104226	−6.8	1609	eMol300397022	−6.7	1660	eMol42849976	−6.7
1559	eMol33332787	−6.8	1610	eMol317731902	−6.7	1661	eMol4709725	−6.7
1560	eMol36015124	−6.8	1611	eMol36015372	−6.7	1662	CHEMBL1413460	−6.7
1561	eMol36015848	−6.8	1612	eMol36015814	−6.7	1663	CHEMBL1440095	−6.7
1562	eMol36734201	−6.8	1613	eMol42925165	−6.7	1664	CHEMBL1595228	−6.7
1563	eMol46210808	−6.8	1614	eMol4717060	−6.7	1665	CHEMBL4068913	−6.7
1564	eMol588384	−6.8	1615	eMol5445007	−6.7	1666	eMol13785933	−6.7
1565	eMol935668	−6.8	1616	eMol560055	−6.7	1667	eMol16352169	−6.7
1566	CHEMBL1492662	−6.8	1617	eMol561535	−6.7	1668	eMol1681902	−6.7
1567	CHEMBL1516504	−6.8	1618	eMol562333	−6.7	1669	eMol18588437	−6.7
1568	eMol1125990	−6.8	1619	CHEMBL1215798	−6.7	1670	eMol18954506	−6.7
1569	eMol1681774	−6.8	1620	CHEMBL1237285	−6.7	1671	eMol193764290	−6.7
1570	eMol206964622	−6.8	1621	CHEMBL1601068	−6.7	1672	eMol20358311	−6.7
1571	eMol206980093	−6.8	1622	CHEMBL3818685	−6.7	1673	eMol208112836	−6.7
1572	eMol24541186	−6.8	1623	eMol1749678	−6.7	1674	eMol23154795	−6.7
1573	eMol25813421	−6.8	1624	eMol19076368	−6.7	1675	eMol25784517	−6.7
1574	eMol25959987	−6.8	1625	eMol20358115	−6.7	1676	eMol25963297	−6.7
1575	eMol27202278	−6.8	1626	eMol25810017	−6.7	1677	eMol26314205	−6.7
1576	eMol27202356	−6.8	1627	eMol27187280	−6.7	1678	eMol27485970	−6.7
1577	eMol3581771	−6.8	1628	eMol27202414	−6.7	1679	eMol36015368	−6.7
1548	eMol25813405	−6.8	1599	CHEMBL416260	−6.7	1650	eMol26052954	−6.7

Table S1. *Continued.*

No.	Compound Name/Code	Docking Score (kcal/mol)	No.	Compound Name/Code	Docking Score (kcal/mol)	No.	Compound Name/Code	Docking Score (kcal/mol)
1680	eMol36015832	−6.7	1731	eMol36015102	−6.7	1782	eMol300252505	−6.7
1681	eMol4730653	−6.7	1732	eMol36015520	−6.7	1783	eMol3494756	−6.7
1682	eMol5444865	−6.7	1733	eMol36016040	−6.7	1784	eMol36913016	−6.7
1683	eMol561447	−6.7	1734	eMol36734203	−6.7	1785	eMol43743366	−6.7
1684	eMol624281	−6.7	1735	eMol45932361	−6.7	1786	eMol4710992	−6.7
1685	eMol679752	−6.7	1736	eMol560185	−6.7	1787	eMol4737014	−6.7
1686	CHEMBL1214790	−6.7	1737	eMol935816	−6.7	1788	eMol53754573	−6.7
1687	CHEMBL1476683	−6.7	1738	CHEMBL1214797	−6.7	1789	eMol54826500	−6.7
1688	CHEMBL1866012	−6.7	1739	CHEMBL1427466	−6.7	1790	eMol565990	−6.7
1689	CHEMBL1872793	−6.7	1740	CHEMBL2134755	−6.7	1791	CHEMBL1593176	−6.7
1690	CHEMBL3819643	−6.7	1741	CHEMBL301308	−6.7	1792	CHEMBL1870195	−6.7
1691	CHEMBL4085469	−6.7	1742	eMol1415360	−6.7	1793	CHEMBL37653	−6.7
1692	eMol1444423	−6.7	1743	eMol16352263	−6.7	1794	CHEMBL38176	−6.7
1693	eMol16351865	−6.7	1744	eMol16390485	−6.7	1795	eMol1119962	−6.7
1694	eMol16351871	−6.7	1745	eMol16805743	−6.7	1796	eMol11970394	−6.7
1695	eMol16370299	−6.7	1746	eMol18551296	−6.7	1797	eMol1266025	−6.7
1696	eMol18550690	−6.7	1747	eMol18879479	−6.7	1798	eMol16351573	−6.7
1697	eMol18780688	−6.7	1748	eMol20337454	−6.7	1799	eMol16758472	−6.7
1698	eMol206924376	−6.7	1749	eMol20382098	−6.7	1800	eMol206910448	−6.7
1699	eMol2180510	−6.7	1750	eMol23238997	−6.7	1801	eMol25813407	−6.7
1700	eMol24555667	−6.7	1751	eMol25901997	−6.7	1802	eMol25896465	−6.7
1701	eMol25815831	−6.7	1752	eMol26281046	−6.7	1803	eMol25942258	−6.7
1702	eMol25902460	−6.7	1753	eMol26337174	−6.7	1804	eMol26141624	−6.7
1703	eMol26141660	−6.7	1754	eMol27187474	−6.7	1805	eMol27187228	−6.7
1704	eMol26293000	−6.7	1755	eMol27202202	−6.7	1806	eMol27202330	−6.7
1705	eMol26310199	−6.7	1756	eMol27202456	−6.7	1807	eMol27202746	−6.7
1706	eMol26319897	−6.7	1757	eMol27202492	−6.7	1808	eMol27202962	−6.7
1707	eMol26344183	−6.7	1758	eMol30341805	−6.7	1809	eMol300229303	−6.7
1708	eMol27115470	−6.7	1759	eMol33346437	−6.7	1810	eMol3004720	−6.7
1709	eMol27202440	−6.7	1760	eMol36734043	−6.7	1811	eMol3381941	−6.7
1710	eMol27202800	−6.7	1761	eMol579396	−6.7	1812	eMol3457653	−6.7
1711	eMol27202922	−6.7	1762	eMol7294872	−6.7	1813	eMol43751233	−6.7
1712	eMol300145258	−6.7	1763	eMol98023415	−6.7	1814	eMol4696216	−6.7
1713	eMol32246803	−6.7	1764	CHEMBL1214791	−6.7	1815	eMol560307	−6.7
1714	eMol36016026	−6.7	1765	CHEMBL3805351	−6.7	1816	eMol7635460	−6.7
1715	eMol7271648	−6.7	1766	CHEMBL4075913	−6.7	1817	CHEMBL3805445	−6.7
1716	eMol956529	−6.7	1767	eMol16352273	−6.7	1818	eMol14855102	−6.7
1717	CHEMBL1449683	−6.7	1768	eMol19076340	−6.7	1819	eMol15245309	−6.7
1718	CHEMBL301330	−6.7	1769	eMol20364228	−6.7	1820	eMol18808193	−6.7
1719	CHEMBL3819609	−6.7	1770	eMol20374086	−6.7	1821	eMol206977399	−6.7
1720	eMol1458294	−6.7	1771	eMol20382812	−6.7	1822	eMol207368461	−6.7
1721	eMol19076334	−6.7	1772	eMol23155886	−6.7	1823	eMol2213417	−6.7
1722	eMol206843414	−6.7	1773	eMol23244991	−6.7	1824	eMol24531994	−6.7
1723	eMol26141650	−6.7	1774	eMol24427994	−6.7	1825	eMol25910177	−6.7
1724	eMol26326538	−6.7	1775	eMol25881344	−6.7	1826	eMol26264081	−6.7
1725	eMol27187404	−6.7	1776	eMol26141340	−6.7	1827	eMol26284998	−6.7
1726	eMol27202292	−6.7	1777	eMol26141622	−6.7	1828	eMol26309549	−6.7
1727	eMol27202364	−6.7	1778	eMol26248577	−6.7	1829	eMol26312290	−6.7
1728	eMol27202408	−6.7	1779	eMol27187214	−6.7	1830	eMol26325538	−6.7
1729	eMol32392848	−6.7	1780	eMol27202308	−6.7	1831	eMol26779126	−6.7
1730	eMol3401751	−6.7	1781	eMol276247938	−6.7	1832	eMol27187346	−6.7
1701	eMol25815831	−6.7	1752	eMol26281046	−6.7	1803	eMol25942258	−6.7

Table S1. *Continued.*

No.	Compound Name/Code	Docking Score (kcal/mol)	No.	Compound Name/Code	Docking Score (kcal/mol)	No.	Compound Name/Code	Docking Score (kcal/mol)
1833	eMol30721735	−6.7	1884	eMol560181	−6.6	1935	eMol7269426	−6.6
1834	eMol43754815	−6.7	1885	eMol7261256	−6.6	1936	eMol96989666	−6.6
1835	eMol4557017	−6.7	1886	CHEMBL1581170	−6.6	1937	CHEMBL1547176	−6.6
1836	eMol46541996	−6.7	1887	CHEMBL1984743	−6.6	1938	CHEMBL1889180	−6.6
1837	eMol562393	−6.7	1888	CHEMBL3818386	−6.6	1939	CHEMBL2355688	−6.6
1838	eMol6157526	−6.7	1889	eMol11879378	−6.6	1940	CHEMBL3805622	−6.6
1839	eMol7268112	−6.7	1890	eMol16352159	−6.6	1941	CHEMBL3819491	−6.6
1840	eMol956427	−6.7	1891	eMol16352189	−6.6	1942	eMol11663659	−6.6
1841	CHEMBL1516226	−6.6	1892	eMol19031808	−6.6	1943	eMol20360019	−6.6
1842	CHEMBL1601841	−6.6	1893	eMol19076342	−6.6	1944	eMol20360818	−6.6
1843	CHEMBL1903920	−6.6	1894	eMol19764926	−6.6	1945	eMol206962178	−6.6
1844	CHEMBL3818857	−6.6	1895	eMol20382919	−6.6	1946	eMol206978287	−6.6
1845	eMol16351859	−6.6	1896	eMol24107121	−6.6	1947	eMol206987243	−6.6
1846	eMol16351867	−6.6	1897	eMol24547204	−6.6	1948	eMol2409707	−6.6
1847	eMol2423428	−6.6	1898	eMol2488724	−6.6	1949	eMol25961959	−6.6
1848	eMol24545576	−6.6	1899	eMol25963261	−6.6	1950	eMol27202412	−6.6
1849	eMol25775755	−6.6	1900	eMol27202314	−6.6	1951	eMol27202484	−6.6
1850	eMol25810167	−6.6	1901	eMol27202500	−6.6	1952	eMol27202814	−6.6
1851	eMol25813411	−6.6	1902	eMol27202528	−6.6	1953	eMol27202844	−6.6
1852	eMol25967144	−6.6	1903	eMol27202892	−6.6	1954	eMol29565470	−6.6
1853	eMol27045525	−6.6	1904	eMol3443403	−6.6	1955	eMol5509853	−6.6
1854	eMol27187442	−6.6	1905	eMol36898008	−6.6	1956	eMol5538254	−6.6
1855	eMol27202206	−6.6	1906	eMol565896	−6.6	1957	eMol560071	−6.6
1856	eMol27202340	−6.6	1907	eMol7701728	−6.6	1958	eMol581330	−6.6
1857	eMol27202770	−6.6	1908	CHEMBL1214867	−6.6	1959	eMol7260196	−6.6
1858	eMol30104210	−6.6	1909	CHEMBL1418314	−6.6	1960	eMol936014	−6.6
1859	eMol43771200	−6.6	1910	CHEMBL1516180	−6.6	1961	eMol98980408	−6.6
1860	eMol45951607	−6.6	1911	eMol11687945	−6.6	1962	eMol99520559	−6.6
1861	eMol50575071	−6.6	1912	eMol16351575	−6.6	1963	CHEMBL1490844	−6.6
1862	eMol565972	−6.6	1913	eMol16407954	−6.6	1964	CHEMBL1880647	−6.6
1863	eMol579916	−6.6	1914	eMol206843424	−6.6	1965	eMol16390181	−6.6
1864	CHEMBL1402158	−6.6	1915	eMol206978315	−6.6	1966	eMol17193486	−6.6
1865	CHEMBL1903268	−6.6	1916	eMol206993297	−6.6	1967	eMol18900038	−6.6
1866	CHEMBL297579	−6.6	1917	eMol24520907	−6.6	1968	eMol206904367	−6.6
1867	eMol13734651	−6.6	1918	eMol24526901	−6.6	1969	eMol25782831	−6.6
1868	eMol16379439	−6.6	1919	eMol25797961	−6.6	1970	eMol25829419	−6.6
1869	eMol20381366	−6.6	1920	eMol25908587	−6.6	1971	eMol25962071	−6.6
1870	eMol20383035	−6.6	1921	eMol27202386	−6.6	1972	eMol26260063	−6.6
1871	eMol206843410	−6.6	1922	eMol27202566	−6.6	1973	eMol26340085	−6.6
1872	eMol206970436	−6.6	1923	eMol27202788	−6.6	1974	eMol26594563	−6.6
1873	eMol24427888	−6.6	1924	eMol27202798	−6.6	1975	eMol27045277	−6.6
1874	eMol25771176	−6.6	1925	eMol27490345	−6.6	1976	eMol27187316	−6.6
1875	eMol25926749	−6.6	1926	eMol3315989	−6.6	1977	eMol27202966	−6.6
1876	eMol26243624	−6.6	1927	eMol36015342	−6.6	1978	eMol3455505	−6.6
1877	eMol26288251	−6.6	1928	eMol43744872	−6.6	1979	eMol43732496	−6.6
1878	eMol27202352	−6.6	1929	eMol45705372	−6.6	1980	CHEMBL1473628	−6.6
1879	eMol27202802	−6.6	1930	eMol46210816	−6.6	1981	CHEMBL3818892	−6.6
1880	eMol2992494	−6.6	1931	eMol46370141	−6.6	1982	CHEMBL3914501	−6.6
1881	eMol3252720	−6.6	1932	eMol4703775	−6.6	1983	eMol16351607	−6.6
1882	eMol3670123	−6.6	1933	eMol4718173	−6.6	1984	eMol16352163	−6.6
1883	eMol5444963	−6.6	1934	eMol6263469	−6.6	1985	eMol16786052	−6.6
1854	eMol27187442	−6.6	1905	eMol36898008	−6.6	1956	eMol5538254	−6.6

Table S1. *Continued.*

No.	Compound Name/Code	Docking Score (kcal/mol)	No.	Compound Name/Code	Docking Score (kcal/mol)	No.	Compound Name/Code	Docking Score (kcal/mol)
1986	eMol19076700	−6.6	2016	eMol27202426	−6.6	2067	eMol1237695	−6.5
1987	eMol206843365	−6.6	2017	eMol27202502	−6.6	2068	eMol18536433	−6.5
1988	eMol2386034	−6.6	2018	eMol30104206	−6.6	2069	eMol19076362	−6.5
1989	eMol24519974	−6.6	2019	eMol36016676	−6.6	2070	eMol20380980	−6.5
1990	eMol26339810	−6.6	2020	eMol45645578	−6.6	2071	eMol23982014	−6.5
1991	eMol27202210	−6.6	2021	eMol4567983	−6.6	2072	eMol2513700	−6.5
1992	eMol27202978	−6.6	2022	eMol46210768	−6.6	2073	eMol2519315	−6.5
1993	eMol2889094	−6.6	2023	eMol4727438	−6.6	2074	eMol25813413	−6.5
1994	eMol289941482	−6.6	2024	eMol6467547	−6.6	2075	eMol25881262	−6.5
1995	eMol2993559	−6.6	2025	eMol7259996	−6.6	2076	eMol26350874	−6.5
1996	eMol3459687	−6.6	2026	eMol7266104	−6.6	2077	eMol27045523	−6.5
1997	eMol36015734	−6.6	2027	CHEMBL1475827	−6.6	2078	eMol27187266	−6.5
1998	eMol36016022	−6.6	2028	CHEMBL249301	−6.6	2079	eMol27202200	−6.5
1999	eMol44057501	−6.6	2029	CHEMBL297442	−6.6	2080	eMol27202530	−6.5
2000	eMol561659	−6.6	2030	eMol16352161	−6.6	2081	eMol36015714	−6.5
2001	CHEMBL1513163	−6.6	2031	eMol1834904	−6.6	2082	eMol36015812	−6.5
2002	CHEMBL1610757	−6.6	2032	eMol18551350	−6.6	2083	eMol36016036	−6.5
2003	CHEMBL3805404	−6.6	2033	eMol206904341	−6.6	2084	eMol36016052	−6.5
2004	eMol1032463	−6.6	2034	eMol206906698	−6.6	2085	eMol36733911	−6.5
2005	eMol16351585	−6.6	2035	eMol206992362	−6.6	2086	eMol36898044	−6.5
2006	eMol16351605	−6.6	2036	eMol25825123	−6.6	2087	eMol45803170	−6.5
2007	eMol206961858	−6.6	2037	eMol25966292	−6.6	2088	eMol561977	−6.5
2008	eMol206985304	−6.6	2038	eMol26263883	−6.6	2089	eMol566052	−6.5
2009	eMol207355363	−6.6	2039	eMol26337698	−6.6	2090	eMol956197	−6.5
2010	eMol25784123	−6.6	2040	eMol317395728	−6.6	2091	CHEMBL111006	−6.5
2011	eMol25813409	−6.6	2041	eMol33346431	−6.6	2092	CHEMBL118001	−6.5
2012	eMol25813427	−6.6	2042	eMol44218497	−6.6	2093	CHEMBL1895705	−6.5
2013	eMol26195582	−6.6	2043	eMol4727713	−6.6	2094	CHEMBL3935405	−6.5
2014	eMol27187274	−6.6	2044	eMol4730084	−6.6	2095	CHEMBL561127	−6.5
1994	eMol289941482	−6.6	2045	eMol587920	−6.6	2096	eMol16352191	−6.5
1995	eMol2993559	−6.6	2046	eMol956475	−6.6	2097	eMol16759756	−6.5
1996	eMol3459687	−6.6	2047	CHEMBL3818297	−6.6	2098	eMol19031731	−6.5
1997	eMol36015734	−6.6	2048	eMol19351378	−6.6	2099	eMol206910196	−6.5
1998	eMol36016022	−6.6	2049	eMol206962232	−6.6	2100	eMol206969636	−6.5
1999	eMol44057501	−6.6	2050	eMol27038267	−6.6	2101	eMol207116159	−6.5
2000	eMol561659	−6.6	2051	eMol27045559	−6.6	2102	eMol23245699	−6.5
2001	CHEMBL1513163	−6.6	2052	eMol27202286	−6.6	2103	eMol25896431	−6.5
2002	CHEMBL1610757	−6.6	2053	eMol27202462	−6.6	2104	eMol26195584	−6.5
2003	CHEMBL3805404	−6.6	2054	eMol29642547	−6.6	2105	eMol27187386	−6.5
2004	eMol1032463	−6.6	2055	eMol3172250	−6.6	2106	eMol27187470	−6.5
2005	eMol16351585	−6.6	2056	eMol36010133	−6.6	2107	eMol27509217	−6.5
2006	eMol16351605	−6.6	2057	eMol36016622	−6.6	2108	eMol2881132	−6.5
2007	eMol206961858	−6.6	2058	eMol43772416	−6.6	2109	eMol300020871	−6.5
2008	eMol206985304	−6.6	2059	eMol46210820	−6.6	2110	eMol43743380	−6.5
2009	eMol207355363	−6.6	2060	eMol561519	−6.6	2111	eMol43995461	−6.5
2010	eMol25784123	−6.6	2061	CHEMBL1475371	−6.5	2112	eMol4706823	−6.5
2011	eMol25813409	−6.6	2062	CHEMBL245490	−6.5	2113	eMol4716299	−6.5
2012	eMol25813427	−6.6	2063	CHEMBL3417654	−6.5	2114	eMol4728654	−6.5
2013	eMol26195582	−6.6	2064	CHEMBL53995	−6.5	2115	eMol560189	−6.5
2014	eMol27187274	−6.6	2065	CHEMBL565080	−6.5	2116	eMol581246	−6.5
2015	eMol27202328	−6.6	2066	eMol1165995	−6.5	2117	eMol639662	−6.5
2007	eMol206961858	−6.6	2037	eMol25966292	−6.6	2088	eMol561977	−6.5

Table S1. *Continued.*

No.	Compound Name/Code	Docking Score (kcal/mol)	No.	Compound Name/Code	Docking Score (kcal/mol)	No.	Compound Name/Code	Docking Score (kcal/mol)
2118	eMol8547353	−6.5	2169	CHEMBL1373044	−6.5	2220	eMol5444931	−6.5
2119	CHEMBL300024	−6.5	2170	CHEMBL1882018	−6.5	2221	eMol7281428	−6.5
2120	CHEMBL314385	−6.5	2171	CHEMBL4076110	−6.5	2222	CHEMBL1310211	−6.5
2121	eMol11758466	−6.5	2172	eMol1120544	−6.5	2223	CHEMBL1434827	−6.5
2122	eMol16351577	−6.5	2173	eMol1386911	−6.5	2224	CHEMBL1592831	−6.5
2123	eMol177539683	−6.5	2174	eMol16352171	−6.5	2225	CHEMBL298576	−6.5
2124	eMol206843485	−6.5	2175	eMol24520475	−6.5	2226	CHEMBL3817977	−6.5
2125	eMol22370932	−6.5	2176	eMol25813401	−6.5	2227	eMol11371397	−6.5
2126	eMol23236781	−6.5	2177	eMol25813415	−6.5	2228	eMol13502306	−6.5
2127	eMol24152474	−6.5	2178	eMol25813435	−6.5	2229	eMol16352257	−6.5
2128	eMol24524791	−6.5	2179	eMol25881328	−6.5	2230	eMol18823378	−6.5
2129	eMol25813431	−6.5	2180	eMol26286432	−6.5	2231	eMol20375967	−6.5
2130	eMol25881340	−6.5	2181	eMol27187342	−6.5	2232	eMol206906624	−6.5
2131	eMol25962331	−6.5	2182	eMol27341385	−6.5	2233	eMol206977577	−6.5
2132	eMol26184916	−6.5	2183	eMol30185530	−6.5	2234	eMol206994514	−6.5
2133	eMol27202430	−6.5	2184	eMol3063037	−6.5	2235	eMol25807807	−6.5
2134	eMol3204435	−6.5	2185	eMol3501366	−6.5	2236	eMol25810159	−6.5
2135	eMol36733941	−6.5	2186	eMol36015620	−6.5	2237	eMol27187032	−6.5
2136	eMol36734171	−6.5	2187	eMol36733903	−6.5	2238	eMol3206345	−6.5
2137	eMol5444999	−6.5	2188	eMol44519141	−6.5	2239	eMol36013033	−6.5
2138	CHEMBL1415081	−6.5	2189	eMol4726940	−6.5	2240	eMol36015822	−6.5
2139	CHEMBL1514215	−6.5	2190	eMol5538220	−6.5	2241	eMol42762132	−6.5
2140	CHEMBL1903537	−6.5	2191	eMol5538898	−6.5	2242	eMol5444995	−6.5
2141	CHEMBL563538	−6.5	2192	eMol6869460	−6.5	2243	CHEMBL301313	−6.5
2142	eMol11903726	−6.5	2193	eMol98690075	−6.5	2244	eMol1035485	−6.5
2143	eMol16373087	−6.5	2194	CHEMBL113883	−6.5	2245	eMol18879511	−6.5
2144	eMol16390221	−6.5	2195	CHEMBL1443220	−6.5	2246	eMol206843430	−6.5
2145	eMol16407934	−6.5	2196	CHEMBL1497333	−6.5	2247	eMol206924378	−6.5
2146	eMol1679112	−6.5	2197	CHEMBL260456	−6.5	2248	eMol206969606	−6.5
2147	eMol19382530	−6.5	2198	CHEMBL51165	−6.5	2249	eMol22375727	−6.5
2148	eMol2001733	−6.5	2199	CHEMBL53479	−6.5	2250	eMol26141688	−6.5
2149	eMol20344244	−6.5	2200	eMol16407962	−6.5	2251	eMol26268026	−6.5
2150	eMol206978824	−6.5	2201	eMol1673994	−6.5	2252	eMol27187466	−6.5
2151	eMol23982214	−6.5	2202	eMol20382826	−6.5	2253	eMol300261713	−6.5
2152	eMol24427912	−6.5	2203	eMol206843412	−6.5	2254	eMol3327781	−6.5
2153	eMol25844601	−6.5	2204	eMol25810097	−6.5	2255	eMol3485283	−6.5
2154	eMol26242028	−6.5	2205	eMol25813425	−6.5	2256	eMol3517784	−6.5
2155	eMol26248549	−6.5	2206	eMol25817107	−6.5	2257	eMol44603525	−6.5
2156	eMol27045467	−6.5	2207	eMol25957029	−6.5	2258	eMol45787891	−6.5
2157	eMol27187234	−6.5	2208	eMol26254010	−6.5	2259	eMol4727426	−6.5
2158	eMol27202310	−6.5	2209	eMol26291793	−6.5	2260	CHEMBL185809	−6.5
2159	eMol3087147	−6.5	2210	eMol27045479	−6.5	2261	CHEMBL3805880	−6.5
2160	eMol36636763	−6.5	2211	eMol27045553	−6.5	2262	eMol11070727	−6.5
2161	eMol4538051	−6.5	2212	eMol27045583	−6.5	2263	eMol1149891	−6.5
2162	eMol45930049	−6.5	2213	eMol27202416	−6.5	2264	eMol11816884	−6.5
2163	eMol5444633	−6.5	2214	eMol2991923	−6.5	2265	eMol16351681	−6.5
2164	eMol566608	−6.5	2215	eMol36734029	−6.5	2266	eMol18931802	−6.5
2165	eMol581404	−6.5	2216	eMol42927497	−6.5	2267	eMol206963325	−6.5
2166	eMol6514347	−6.5	2217	eMol45904698	−6.5	2268	eMol26349730	−6.5
2167	eMol7291156	−6.5	2218	eMol4718135	−6.5	2269	eMol27187320	−6.5
2168	CHEMBL1323375	−6.5	2219	eMol4727659	−6.5	2270	eMol27202424	−6.5
2139	CHEMBL1514215	−6.5	2190	eMol5538220	−6.5	2241	eMol42762132	−6.5

Table S1. *Continued.*

No.	Compound Name/Code	Docking Score (kcal/mol)	No.	Compound Name/Code	Docking Score (kcal/mol)	No.	Compound Name/Code	Docking Score (kcal/mol)
2271	eMol27202446	−6.5	2322	eMol15301394	−6.4	2373	eMol3255850	−6.4
2272	eMol27202486	−6.5	2323	eMol1679964	−6.4	2374	eMol32661616	−6.4
2273	eMol27202536	−6.5	2324	eMol206962711	−6.4	2375	eMol43995617	−6.4
2274	eMol29968406	−6.5	2325	eMol24306794	−6.4	2376	eMol46210770	−6.4
2275	eMol300149359	−6.5	2326	eMol25810105	−6.4	2377	eMol7266174	−6.4
2276	eMol33338072	−6.5	2327	eMol26299887	−6.4	2378	eMol9815169	−6.4
2277	eMol35387961	−6.5	2328	eMol27187282	−6.4	2379	CHEMBL3417641	−6.4
2278	eMol36010145	−6.5	2329	eMol27202230	−6.4	2380	eMol16374771	−6.4
2279	eMol36015328	−6.5	2330	eMol27202786	−6.4	2381	eMol193274586	−6.4
2280	eMol36015854	−6.5	2331	eMol316926407	−6.4	2382	eMol20377949	−6.4
2281	eMol36016050	−6.5	2332	eMol32246985	−6.4	2383	eMol24525072	−6.4
2282	eMol36898026	−6.5	2333	eMol3407217	−6.4	2384	eMol26291212	−6.4
2283	eMol43746855	−6.5	2334	eMol3501354	−6.4	2385	eMol26343081	−6.4
2284	eMol566444	−6.5	2335	eMol36898002	−6.4	2386	eMol27202212	−6.4
2285	CHEMBL189170	−6.5	2336	eMol4467282	−6.4	2387	eMol27202404	−6.4
2286	CHEMBL3804860	−6.5	2337	eMol561361	−6.4	2388	eMol27202418	−6.4
2287	CHEMBL3818548	−6.5	2338	eMol679600	−6.4	2389	eMol27202468	−6.4
2288	eMol16373089	−6.5	2339	CHEMBL1214865	−6.4	2390	eMol27349409	−6.4
2289	eMol16385710	−6.5	2340	CHEMBL3806281	−6.4	2391	eMol29623495	−6.4
2290	eMol1679132	−6.5	2341	eMol1131454	−6.4	2392	eMol3245568	−6.4
2291	eMol18550756	−6.5	2342	eMol16379821	−6.4	2393	eMol34103610	−6.4
2292	eMol2166967	−6.5	2343	eMol16385692	−6.4	2394	eMol36015842	−6.4
2293	eMol25810173	−6.5	2344	eMol1682570	−6.4	2395	eMol3663282	−6.4
2294	eMol27202194	−6.5	2345	eMol23180310	−6.4	2396	eMol5444549	−6.4
2295	eMol27202370	−6.5	2346	eMol23254399	−6.4	2397	CHEMBL1321779	−6.4
2296	eMol27202976	−6.5	2347	eMol26131908	−6.4	2398	CHEMBL1577901	−6.4
2297	eMol3518572	−6.5	2348	eMol27182628	−6.4	2399	CHEMBL294968	−6.4
2298	eMol36015838	−6.5	2349	eMol3055183	−6.4	2400	CHEMBL3417643	−6.4
2299	eMol44017453	−6.5	2350	eMol3316007	−6.4	2401	CHEMBL3818714	−6.4
2300	eMol46210810	−6.5	2351	eMol3707946	−6.4	2402	eMol1448891	−6.4
2301	eMol5444547	−6.5	2352	eMol5445035	−6.4	2403	eMol19248288	−6.4
2302	eMol5779863	−6.5	2353	eMol7270136	−6.4	2404	eMol26288149	−6.4
2303	eMol6806127	−6.5	2354	CHEMBL1396361	−6.4	2405	eMol27186996	−6.4
2304	eMol206985650	−6.4	2355	CHEMBL1593196	−6.4	2406	eMol27202294	−6.4
2305	eMol26141664	−6.4	2356	CHEMBL184749	−6.4	2407	eMol27202538	−6.4
2306	eMol26236636	−6.4	2357	CHEMBL3417618	−6.4	2408	eMol27202806	−6.4
2307	eMol27202220	−6.4	2358	CHEMBL3818360	−6.4	2409	eMol27202890	−6.4
2308	eMol27202276	−6.4	2359	eMol11905854	−6.4	2410	eMol2871294	−6.4
2309	eMol27490285	−6.4	2360	eMol16407926	−6.4	2411	eMol2992552	−6.4
2310	eMol2768996	−6.4	2361	eMol16408016	−6.4	2412	eMol300250258	−6.4
2311	eMol3376801	−6.4	2362	eMol16408020	−6.4	2413	eMol316452059	−6.4
2312	eMol36015840	−6.4	2363	eMol17163712	−6.4	2414	eMol3443675	−6.4
2313	eMol36015962	−6.4	2364	eMol25816257	−6.4	2415	eMol3494000	−6.4
2314	eMol45705672	−6.4	2365	eMol25966290	−6.4	2416	eMol36015116	−6.4
2315	eMol4759218	−6.4	2366	eMol26289584	−6.4	2417	eMol36733933	−6.4
2316	eMol5445013	−6.4	2367	eMol27187034	−6.4	2418	eMol43731752	−6.4
2317	CHEMBL1391688	−6.4	2368	eMol27202748	−6.4	2419	eMol43743368	−6.4
2318	CHEMBL1553786	−6.4	2369	eMol30104222	−6.4	2420	eMol46210772	−6.4
2319	CHEMBL3804979	−6.4	2370	eMol316975064	−6.4	2421	eMol4694025	−6.4
2320	CHEMBL51620	−6.4	2371	eMol31780057	−6.4	2422	eMol561611	−6.4
2321	eMol13491303	−6.4	2372	eMol3220074	−6.4	2423	CHEMBL1215651	−6.4
2292	eMol2166967	−6.5	2343	eMol16385692	−6.4	2394	eMol36015842	−6.4

Table S1. *Continued.*

No.	Compound Name/Code	Docking Score (kcal/mol)	No.	Compound Name/Code	Docking Score (kcal/mol)	No.	Compound Name/Code	Docking Score (kcal/mol)
2424	eMol16056917	−6.4	2475	eMol16379145	−6.4	2526	eMol11671108	−6.3
2425	eMol16379139	−6.4	2476	eMol16379809	−6.4	2527	eMol19031792	−6.3
2426	eMol18536843	−6.4	2477	eMol16390077	−6.4	2528	eMol206843422	−6.3
2427	eMol24021853	−6.4	2478	eMol23254331	−6.4	2529	eMol24302059	−6.3
2428	eMol26196308	−6.4	2479	eMol26347234	−6.4	2530	eMol24534942	−6.3
2429	eMol26349111	−6.4	2480	eMol27045491	−6.4	2531	eMol26315552	−6.3
2430	eMol27202228	−6.4	2481	eMol27202428	−6.4	2532	eMol27202964	−6.3
2431	eMol27202812	−6.4	2482	eMol27202816	−6.4	2533	eMol34181785	−6.3
2432	eMol29585788	−6.4	2483	eMol29633215	−6.4	2534	eMol36010131	−6.3
2433	eMol36015522	−6.4	2484	eMol30104214	−6.4	2535	eMol36015330	−6.3
2434	eMol45585828	−6.4	2485	eMol35700126	−6.4	2536	eMol36016278	−6.3
2435	eMol4700479	−6.4	2486	eMol36015614	−6.4	2537	eMol36016614	−6.3
2436	eMol50159532	−6.4	2487	eMol43745108	−6.4	2538	eMol43747047	−6.3
2437	eMol581334	−6.4	2488	eMol44123579	−6.4	2539	eMol46346846	−6.3
2438	CHEMBL1366795	−6.4	2489	eMol45951547	−6.4	2540	eMol4702936	−6.3
2439	CHEMBL1608564	−6.4	2490	eMol5444515	−6.4	2541	eMol52129783	−6.3
2440	CHEMBL3959539	−6.4	2491	eMol5444959	−6.4	2542	eMol5538570	−6.3
2441	CHEMBL47230	−6.4	2492	eMol6523821	−6.4	2543	eMol7289730	−6.3
2442	eMol11893182	−6.4	2493	CHEMBL1345911	−6.4	2544	CHEMBL1169938	−6.3
2443	eMol19250818	−6.4	2494	CHEMBL299336	−6.4	2545	CHEMBL1394032	−6.3
2444	eMol20350994	−6.4	2495	CHEMBL50538	−6.4	2546	CHEMBL341356	−6.3
2445	eMol23246486	−6.4	2496	eMol11915751	−6.4	2547	CHEMBL49924	−6.3
2446	eMol23248155	−6.4	2497	eMol16352259	−6.4	2548	eMol100292265	−6.3
2447	eMol24473893	−6.4	2498	eMol20333676	−6.4	2549	eMol1748351	−6.3
2448	eMol25797965	−6.4	2499	eMol20351269	−6.4	2550	eMol18550708	−6.3
2449	eMol27202368	−6.4	2500	eMol206907679	−6.4	2551	eMol26141690	−6.3
2450	eMol276247966	−6.4	2501	eMol22050213	−6.4	2552	eMol26249989	−6.3
2451	eMol300994278	−6.4	2502	eMol24423650	−6.4	2553	eMol27182514	−6.3
2452	eMol32246425	−6.4	2503	eMol24539609	−6.4	2554	eMol27187000	−6.3
2453	eMol36010141	−6.4	2504	eMol26141636	−6.4	2555	eMol27187420	−6.3
2454	eMol36015364	−6.4	2505	eMol26263630	−6.4	2556	eMol27202222	−6.3
2455	eMol36015864	−6.4	2506	eMol26297652	−6.4	2557	eMol27202366	−6.3
2456	eMol36016154	−6.4	2507	eMol27187354	−6.4	2558	eMol29456700	−6.3
2457	eMol3666911	−6.4	2508	eMol27187408	−6.4	2559	eMol2997342	−6.3
2458	eMol4388249	−6.4	2509	eMol27202374	−6.4	2560	eMol3131192	−6.3
2459	eMol4515790	−6.4	2510	eMol27202448	−6.4	2561	eMol32170345	−6.3
2460	eMol5444429	−6.4	2511	eMol27490301	−6.4	2562	eMol3464492	−6.3
2461	eMol5444627	−6.4	2512	eMol32246825	−6.4	2563	eMol3523182	−6.3
2462	eMol5444961	−6.4	2513	eMol3352043	−6.4	2564	eMol36015110	−6.3
2463	eMol581222	−6.4	2514	eMol3380583	−6.4	2565	eMol36015528	−6.3
2464	eMol7259360	−6.4	2515	eMol36015340	−6.4	2566	eMol43716283	−6.3
2465	eMol7281766	−6.4	2516	eMol36379003	−6.4	2567	eMol43757857	−6.3
2466	ChEBI94303	−6.4	2517	eMol36733939	−6.4	2568	eMol679698	−6.3
2467	CHEMBL1567670	−6.4	2518	eMol42849980	−6.4	2569	eMol7272218	−6.3
2468	CHEMBL422227	−6.4	2519	eMol565722	−6.4	2570	eMol7282414	−6.3
2469	CHEMBL50824	−6.4	2520	eMol7274760	−6.4	2571	eMol7284218	−6.3
2470	eMol129040	−6.4	2521	eMol7282976	−6.4	2572	CHEMBL110431	−6.3
2471	eMol1319929	−6.4	2522	CHEMBL1358305	−6.3	2573	CHEMBL1214796	−6.3
2472	eMol1386917	−6.4	2523	CHEMBL1555962	−6.3	2574	CHEMBL1877088	−6.3
2473	eMol1518824	−6.4	2524	CHEMBL3397268	−6.3	2575	eMol1444337	−6.3
2474	eMol16056959	−6.4	2525	CHEMBL418632	−6.3	2576	eMol16379079	−6.3
2445	eMol23246486	−6.4	2496	eMol11915751	−6.4	2547	CHEMBL49924	−6.3

Table S1. *Continued.*

No.	Compound Name/Code	Docking Score (kcal/mol)	No.	Compound Name/Code	Docking Score (kcal/mol)	No.	Compound Name/Code	Docking Score (kcal/mol)
2577	eMol16379613	−6.3	2628	eMol206977208	−6.3	2679	eMol26242030	−6.3
2578	eMol18899052	−6.3	2629	eMol24423574	−6.3	2680	eMol26245163	−6.3
2579	eMol23255741	−6.3	2630	eMol25775765	−6.3	2681	eMol26249746	−6.3
2580	eMol24113467	−6.3	2631	eMol25896467	−6.3	2682	eMol27202296	−6.3
2581	eMol25813433	−6.3	2632	eMol26141634	−6.3	2683	eMol27490295	−6.3
2582	eMol26283738	−6.3	2633	eMol27187480	−6.3	2684	eMol27490303	−6.3
2583	eMol27182880	−6.3	2634	eMol27202494	−6.3	2685	eMol27490307	−6.3
2584	eMol27202478	−6.3	2635	eMol277684393	−6.3	2686	eMol3393385	−6.3
2585	eMol31857901	−6.3	2636	eMol3017868	−6.3	2687	eMol35672400	−6.3
2586	eMol31950403	−6.3	2637	eMol36015338	−6.3	2688	eMol36016046	−6.3
2587	eMol3302941	−6.3	2638	eMol42713805	−6.3	2689	eMol36016282	−6.3
2588	eMol36016020	−6.3	2639	eMol784260	−6.3	2690	eMol43749119	−6.3
2589	eMol43995169	−6.3	2640	CHEMBL1898021	−6.3	2691	eMol4727272	−6.3
2590	eMol45904696	−6.3	2641	CHEMBL3397270	−6.3	2692	CHEMBL1700745	−6.3
2591	eMol48213031	−6.3	2642	CHEMBL3417621	−6.3	2693	CHEMBL1892332	−6.3
2592	eMol51984479	−6.3	2643	CHEMBL50207	−6.3	2694	CHEMBL298073	−6.3
2593	eMol5444431	−6.3	2644	eMol11069169	−6.3	2695	CHEMBL3627817	−6.3
2594	eMol5476297	−6.3	2645	eMol16379789	−6.3	2696	CHEMBL54233	−6.3
2595	eMol5660295	−6.3	2646	eMol16867315	−6.3	2697	eMol11990345	−6.3
2596	eMol6491039	−6.3	2647	eMol1749410	−6.3	2698	eMol1456432	−6.3
2597	CHEMBL1902987	−6.3	2648	eMol2169306	−6.3	2699	eMol16375739	−6.3
2598	CHEMBL2296351	−6.3	2649	eMol24113595	−6.3	2700	eMol16379719	−6.3
2599	CHEMBL597034	−6.3	2650	eMol26141520	−6.3	2701	eMol206921739	−6.3
2600	eMol1319931	−6.3	2651	eMol26313303	−6.3	2702	eMol206965396	−6.3
2601	eMol16373093	−6.3	2652	eMol26351308	−6.3	2703	eMol24104760	−6.3
2602	eMol1763812	−6.3	2653	eMol27187510	−6.3	2704	eMol27202804	−6.3
2603	eMol2308402	−6.3	2654	eMol27202346	−6.3	2705	eMol31991509	−6.3
2604	eMol24112863	−6.3	2655	eMol29577066	−6.3	2706	eMol34103848	−6.3
2605	eMol24507725	−6.3	2656	eMol30308339	−6.3	2707	eMol34206757	−6.3
2606	eMol24659821	−6.3	2657	eMol316473463	−6.3	2708	eMol36015788	−6.3
2607	eMol2482511	−6.3	2658	eMol36010125	−6.3	2709	eMol36016044	−6.3
2608	eMol26141620	−6.3	2659	eMol36015672	−6.3	2710	eMol36016122	−6.3
2609	eMol26570013	−6.3	2660	eMol43686162	−6.3	2711	eMol36016422	−6.3
2610	eMol27187014	−6.3	2661	eMol43749045	−6.3	2712	eMol36016572	−6.3
2611	eMol27202458	−6.3	2662	eMol43771176	−6.3	2713	eMol43777076	−6.3
2612	eMol27202838	−6.3	2663	eMol45932359	−6.3	2714	eMol4694984	−6.3
2613	eMol29959342	−6.3	2664	eMol4896150	−6.3	2715	eMol5785536	−6.3
2614	eMol32014364	−6.3	2665	eMol5839003	−6.3	2716	eMol7267392	−6.3
2615	eMol36015646	−6.3	2666	eMol7259022	−6.3	2717	CHEMBL1214792	−6.3
2616	eMol45787959	−6.3	2667	CHEMBL1442224	−6.3	2718	CHEMBL1887151	−6.3
2617	CHEMBL1879611	−6.3	2668	CHEMBL1716610	−6.3	2719	CHEMBL2140826	−6.3
2618	CHEMBL1971499	−6.3	2669	CHEMBL1999721	−6.3	2720	CHEMBL3417650	−6.3
2619	CHEMBL3759501	−6.3	2670	CHEMBL51005	−6.3	2721	eMol13522798	−6.3
2620	CHEMBL3955772	−6.3	2671	eMol101721906	−6.3	2722	eMol16760834	−6.3
2621	CHEMBL400335	−6.3	2672	eMol16374577	−6.3	2723	eMol16768525	−6.3
2622	CHEMBL50895	−6.3	2673	eMol16407950	−6.3	2724	eMol179424331	−6.3
2623	eMol13396564	−6.3	2674	eMol18551172	−6.3	2725	eMol19248286	−6.3
2624	eMol1438752	−6.3	2675	eMol25909136	−6.3	2726	eMol206843460	−6.3
2625	eMol16379783	−6.3	2676	eMol26141642	−6.3	2727	eMol206911215	−6.3
2626	eMol18961318	−6.3	2677	eMol26141692	−6.3	2728	eMol25813403	−6.3
2627	eMol206910400	−6.3	2678	eMol26141696	−6.3	2729	eMol25889438	−6.3
2598	CHEMBL2296351	−6.3	2649	eMol24113595	−6.3	2700	eMol16379719	−6.3

Table S1. *Continued.*

No.	Compound Name/Code	Docking Score (kcal/mol)	No.	Compound Name/Code	Docking Score (kcal/mol)	No.	Compound Name/Code	Docking Score (kcal/mol)
2730	eMol27202476	−6.3	2781	eMol27202856	−6.2	2832	eMol1749778	−6.2
2731	eMol27350313	−6.3	2782	eMol277684403	−6.2	2833	eMol206904461	−6.2
2732	eMol35670816	−6.3	2783	eMol29272190	−6.2	2834	eMol25797959	−6.2
2733	eMol3792963	−6.3	2784	eMol31758474	−6.2	2835	eMol27202474	−6.2
2734	eMol43943011	−6.3	2785	eMol32245085	−6.2	2836	eMol33346439	−6.2
2735	eMol4505656	−6.3	2786	eMol36015358	−6.2	2837	eMol3518574	−6.2
2736	eMol4736890	−6.3	2787	eMol36015498	−6.2	2838	eMol3651674	−6.2
2737	eMol4839170	−6.3	2788	eMol36016042	−6.2	2839	eMol43780682	−6.2
2738	eMol565320	−6.3	2789	eMol36016160	−6.2	2840	eMol4461665	−6.2
2739	CHEMBL1325661	−6.3	2790	eMol43731196	−6.2	2841	eMol4540512	−6.2
2740	CHEMBL1545153	−6.3	2791	eMol46572914	−6.2	2842	eMol579696	−6.2
2741	CHEMBL1600359	−6.3	2792	eMol4887803	−6.2	2843	eMol587574	−6.2
2742	CHEMBL1882595	−6.3	2793	eMol624287	−6.2	2844	CHEMBL1560726	−6.2
2743	CHEMBL1901940	−6.3	2794	eMol7276794	−6.2	2845	CHEMBL3644258	−6.2
2744	CHEMBL3099881	−6.3	2795	eMol9989330	−6.2	2846	CHEMBL3819208	−6.2
2745	CHEMBL48826	−6.3	2796	CHEMBL3417652	−6.2	2847	eMol11696283	−6.2
2746	eMol1201946	−6.3	2797	eMol17485893	−6.2	2848	eMol13542778	−6.2
2747	eMol13550711	−6.3	2798	eMol25816263	−6.2	2849	eMol16374765	−6.2
2748	eMol16351583	−6.3	2799	eMol25963003	−6.2	2850	eMol16379793	−6.2
2749	eMol16374759	−6.3	2800	eMol29639001	−6.2	2851	eMol16379835	−6.2
2750	eMol16407932	−6.3	2801	eMol3155052	−6.2	2852	eMol1746769	−6.2
2751	eMol1682098	−6.3	2802	eMol3244958	−6.2	2853	eMol206985138	−6.2
2752	eMol19031739	−6.3	2803	eMol34147565	−6.2	2854	eMol25909732	−6.2
2753	eMol20382915	−6.3	2804	eMol42849978	−6.2	2855	eMol26243041	−6.2
2754	eMol27045537	−6.3	2805	eMol43678568	−6.2	2856	eMol26348427	−6.2
2755	eMol27202974	−6.3	2806	eMol43685794	−6.2	2857	eMol27182890	−6.2
2756	eMol3024862	−6.3	2807	eMol43757813	−6.2	2858	eMol27202332	−6.2
2757	eMol33332789	−6.3	2808	eMol44074398	−6.2	2859	eMol3050679	−6.2
2758	eMol3517993	−6.3	2809	eMol46210814	−6.2	2860	eMol3165810	−6.2
2759	eMol35670712	−6.3	2810	eMol4935136	−6.2	2861	eMol34126656	−6.2
2760	eMol35670806	−6.3	2811	eMol5445011	−6.2	2862	eMol3516732	−6.2
2761	eMol36016412	−6.3	2812	eMol7289098	−6.2	2863	eMol36015356	−6.2
2762	eMol36016546	−6.3	2813	eMol7293496	−6.2	2864	eMol36734199	−6.2
2763	eMol4718351	−6.3	2814	CHEMBL1499094	−6.2	2865	eMol44000007	−6.2
2764	eMol5444399	−6.3	2815	eMol11939519	−6.2	2866	eMol5444827	−6.2
2765	eMol5444655	−6.3	2816	eMol14273668	−6.2	2867	eMol5445017	−6.2
2766	eMol8066589	−6.3	2817	eMol193281590	−6.2	2868	eMol7266492	−6.2
2767	CHEMBL1301253	−6.2	2818	eMol1995647	−6.2	2869	CHEMBL1478140	−6.2
2768	CHEMBL1305555	−6.2	2819	eMol206843420	−6.2	2870	CHEMBL1514702	−6.2
2769	CHEMBL1443325	−6.2	2820	eMol25938966	−6.2	2871	CHEMBL296038	−6.2
2770	CHEMBL364326	−6.2	2821	eMol26230180	−6.2	2872	CHEMBL3804951	−6.2
2771	CHEMBL49199	−6.2	2822	eMol26288616	−6.2	2873	eMol2123648	−6.2
2772	eMol11054214	−6.2	2823	eMol27202952	−6.2	2874	eMol2210792	−6.2
2773	eMol16373091	−6.2	2824	eMol30078849	−6.2	2875	eMol2388346	−6.2
2774	eMol16379797	−6.2	2825	eMol36015866	−6.2	2876	eMol24093211	−6.2
2775	eMol18897534	−6.2	2826	eMol36510871	−6.2	2877	eMol24113477	−6.2
2776	eMol18940279	−6.2	2827	eMol43782296	−6.2	2878	eMol24505508	−6.2
2777	eMol206907052	−6.2	2828	eMol4486344	−6.2	2879	eMol26264906	−6.2
2778	eMol25902692	−6.2	2829	eMol45706150	−6.2	2880	eMol27202198	−6.2
2779	eMol26250756	−6.2	2830	eMol7260242	−6.2	2881	eMol27202406	−6.2
2780	eMol27186994	−6.2	2831	CHEMBL3923521	−6.2	2882	eMol32163535	−6.2
2751	eMol1682098	−6.3	2802	eMol3244958	−6.2	2853	eMol206985138	−6.2

Table S1. *Continued.*

No.	Compound Name/Code	Docking Score (kcal/mol)	No.	Compound Name/Code	Docking Score (kcal/mol)	No.	Compound Name/Code	Docking Score (kcal/mol)
2883	eMol36733919	−6.2	2934	eMol11921274	−6.2	2985	eMol32661622	−6.1
2884	eMol43743426	−6.2	2935	eMol13485875	−6.2	2986	eMol3518576	−6.1
2885	eMol43774431	−6.2	2936	eMol16351807	−6.2	2987	eMol36010129	−6.1
2886	eMol45922019	−6.2	2937	eMol17219415	−6.2	2988	eMol36888157	−6.1
2887	eMol5538224	−6.2	2938	eMol20382754	−6.2	2989	eMol42616136	−6.1
2888	CHEMBL281172	−6.2	2939	eMol2362615	−6.2	2990	eMol7297106	−6.1
2889	CHEMBL298152	−6.2	2940	eMol25784537	−6.2	2991	eMol935440	−6.1
2890	CHEMBL3936506	−6.2	2941	eMol25784541	−6.2	2992	eMol9814195	−6.1
2891	CHEMBL42991	−6.2	2942	eMol27202452	−6.2	2993	CHEMBL1473299	−6.1
2892	CHEMBL50206	−6.2	2943	eMol32661606	−6.2	2994	CHEMBL344096	−6.1
2893	eMol11874754	−6.2	2944	eMol3374251	−6.2	2995	CHEMBL3805207	−6.1
2894	eMol16379617	−6.2	2945	eMol34147303	−6.2	2996	eMol1118798	−6.1
2895	eMol1673934	−6.2	2946	eMol36010157	−6.2	2997	eMol1266015	−6.1
2896	eMol2025066	−6.2	2947	eMol36015756	−6.2	2998	eMol17485895	−6.1
2897	eMol20341799	−6.2	2948	eMol36015934	−6.2	2999	eMol179180654	−6.1
2898	eMol32247725	−6.2	2949	eMol36015942	−6.2	3000	eMol19031804	−6.1
2899	eMol34207047	−6.2	2950	eMol3988552	−6.2	3001	eMol25814743	−6.1
2900	eMol36010087	−6.2	2951	eMol4695032	−6.2	3002	eMol25819227	−6.1
2901	eMol36015844	−6.2	2952	eMol4714641	−6.2	3003	eMol25825139	−6.1
2902	eMol36016018	−6.2	2953	eMol50288672	−6.2	3004	eMol26236634	−6.1
2903	eMol43416567	−6.2	2954	eMol5444631	−6.2	3005	eMol27045475	−6.1
2904	eMol43704543	−6.2	2955	eMol5444939	−6.2	3006	eMol27202772	−6.1
2905	eMol43777016	−6.2	2956	eMol7267352	−6.2	3007	eMol27202950	−6.1
2906	eMol5444507	−6.2	2957	CHEMBL1214795	−6.2	3008	eMol31832997	−6.1
2907	eMol82729703	−6.2	2958	CHEMBL260470	−6.2	3009	eMol33657715	−6.1
2908	CHEMBL1304839	−6.2	2959	eMol16056885	−6.2	3010	eMol36015132	−6.1
2909	CHEMBL50303	−6.2	2960	eMol16379583	−6.2	3011	eMol36015606	−6.1
2910	eMol1221018	−6.2	2961	eMol18551360	−6.2	3012	eMol36016612	−6.1
2911	eMol13484898	−6.2	2962	eMol18877796	−6.2	3013	eMol36888187	−6.1
2912	eMol19032400	−6.2	2963	eMol2234154	−6.2	3014	eMol4550433	−6.1
2913	eMol232791660	−6.2	2964	eMol27202790	−6.2	3015	eMol4709963	−6.1
2914	eMol24432133	−6.2	2965	eMol2889096	−6.2	3016	eMol5444425	−6.1
2915	eMol24453864	−6.2	2966	eMol30719809	−6.2	3017	eMol5444433	−6.1
2916	eMol25810033	−6.2	2967	eMol36015730	−6.2	3018	eMol5538292	−6.1
2917	eMol26251102	−6.2	2968	eMol43995579	−6.2	3019	eMol684014	−6.1
2918	eMol27187020	−6.2	2969	eMol559945	−6.2	3020	eMol7287216	−6.1
2919	eMol27202450	−6.2	2970	eMol681842	−6.2	3021	CHEMBL3805895	−6.1
2920	eMol301525116	−6.2	2971	eMol7288900	−6.2	3022	CHEMBL47554	−6.1
2921	eMol3536592	−6.2	2972	eMol8858539	−6.2	3023	eMol11677024	−6.1
2922	eMol36015306	−6.2	2973	CHEMBL1331239	−6.1	3024	eMol16379077	−6.1
2923	eMol36015826	−6.2	2974	CHEMBL19730	−6.1	3025	eMol2306401	−6.1
2924	eMol36016552	−6.2	2975	CHEMBL3945396	−6.1	3026	eMol24113837	−6.1
2925	eMol36016678	−6.2	2976	CHEMBL52119	−6.1	3027	eMol24659819	−6.1
2926	eMol36733945	−6.2	2977	CHEMBL53036	−6.1	3028	eMol2467293	−6.1
2927	eMol4731515	−6.2	2978	eMol13858043	−6.1	3029	eMol27187350	−6.1
2928	eMol5444853	−6.2	2979	eMol16379067	−6.1	3030	eMol34126716	−6.1
2929	eMol579572	−6.2	2980	eMol20347647	−6.1	3031	eMol36015312	−6.1
2930	CHEMBL184051	−6.2	2981	eMol27045587	−6.1	3032	eMol36909907	−6.1
2931	CHEMBL297908	−6.2	2982	eMol279157786	−6.1	3033	eMol43744868	−6.1
2932	CHEMBL3644257	−6.2	2983	eMol316931780	−6.1	3034	eMol44045654	−6.1
2933	CHEMBL52963	−6.2	2984	eMol32246751	−6.1	3035	eMol5444329	−6.1
2904	eMol43704543	−6.2	2955	eMol5444939	−6.2	3006	eMol27202772	−6.1

Table S1. *Continued.*

No.	Compound Name/Code	Docking Score (kcal/mol)	No.	Compound Name/Code	Docking Score (kcal/mol)	No.	Compound Name/Code	Docking Score (kcal/mol)
3036	eMol5444617	−6.1	3087	eMol49023259	−6.1	3138	eMol5444443	−6.1
3037	eMol681908	−6.1	3088	eMol52239931	−6.1	3139	eMol5444449	−6.1
3038	CHEMBL298261	−6.1	3089	eMol561665	−6.1	3140	eMol5444855	−6.1
3039	CHEMBL3417657	−6.1	3090	eMol562441	−6.1	3141	CHEMBL1415373	−6.1
3040	eMol16379633	−6.1	3091	eMol8310757	−6.1	3142	CHEMBL1548408	−6.1
3041	eMol16795339	−6.1	3092	eMol98685524	−6.1	3143	CHEMBL3088320	−6.1
3042	eMol26348928	−6.1	3093	CHEMBL1307530	−6.1	3144	eMol1035519	−6.1
3043	eMol27202834	−6.1	3094	CHEMBL1437582	−6.1	3145	eMol1226533	−6.1
3044	eMol27202854	−6.1	3095	CHEMBL3417630	−6.1	3146	eMol13084309	−6.1
3045	eMol2993561	−6.1	3096	CHEMBL54011	−6.1	3147	eMol16351861	−6.1
3046	eMol29970720	−6.1	3097	eMol2208787	−6.1	3148	eMol18930908	−6.1
3047	eMol3315627	−6.1	3098	eMol2222259	−6.1	3149	eMol1990456	−6.1
3048	eMol3398875	−6.1	3099	eMol2306586	−6.1	3150	eMol20382752	−6.1
3049	eMol35670480	−6.1	3100	eMol27186988	−6.1	3151	eMol27485968	−6.1
3050	eMol36016424	−6.1	3101	eMol27187200	−6.1	3152	eMol29991603	−6.1
3051	eMol36016438	−6.1	3102	eMol27202436	−6.1	3153	eMol300148591	−6.1
3052	eMol36016640	−6.1	3103	eMol27202928	−6.1	3154	eMol3052111	−6.1
3053	eMol36016646	−6.1	3104	eMol27202930	−6.1	3155	eMol317379879	−6.1
3054	eMol5444611	−6.1	3105	eMol2742270	−6.1	3156	eMol32245541	−6.1
3055	eMol5445001	−6.1	3106	eMol27490349	−6.1	3157	eMol32246329	−6.1
3056	eMol5538612	−6.1	3107	eMol2992536	−6.1	3158	eMol36733917	−6.1
3057	eMol7280788	−6.1	3108	eMol3315543	−6.1	3159	eMol43789927	−6.1
3058	eMol7293044	−6.1	3109	eMol36015534	−6.1	3160	eMol4388901	−6.1
3059	eMol8858469	−6.1	3110	eMol36015810	−6.1	3161	eMol4716784	−6.1
3060	eMol936598	−6.1	3111	eMol36016554	−6.1	3162	eMol49902893	−6.1
3061	eMol98721312	−6.1	3112	eMol36898000	−6.1	3163	eMol5444615	−6.1
3062	CHEMBL1520424	−6.1	3113	eMol44590310	−6.1	3164	eMol5444647	−6.1
3063	CHEMBL1711522	−6.1	3114	eMol4694279	−6.1	3165	eMol5538248	−6.1
3064	CHEMBL1885709	−6.1	3115	eMol4702837	−6.1	3166	eMol5538440	−6.1
3065	CHEMBL3417648	−6.1	3116	eMol49186053	−6.1	3167	eMol5827739	−6.1
3066	CHEMBL45717	−6.1	3117	eMol5538746	−6.1	3168	eMol629457	−6.1
3067	eMol11936467	−6.1	3118	CHEMBL1406694	−6.1	3169	CHEMBL1449112	−6.1
3068	eMol13452524	−6.1	3119	eMol11943360	−6.1	3170	CHEMBL297686	−6.1
3069	eMol20374348	−6.1	3120	eMol16374583	−6.1	3171	CHEMBL3417642	−6.1
3070	eMol2213399	−6.1	3121	eMol179170457	−6.1	3172	CHEMBL3951235	−6.1
3071	eMol24428010	−6.1	3122	eMol2209089	−6.1	3173	eMol16374795	−6.1
3072	eMol24652558	−6.1	3123	eMol23993871	−6.1	3174	eMol16379071	−6.1
3073	eMol25775757	−6.1	3124	eMol25782469	−6.1	3175	eMol19385162	−6.1
3074	eMol25877073	−6.1	3125	eMol26656725	−6.1	3176	eMol1975589	−6.1
3075	eMol27187348	−6.1	3126	eMol27202784	−6.1	3177	eMol25816245	−6.1
3076	eMol27202434	−6.1	3127	eMol27490351	−6.1	3178	eMol27182636	−6.1
3077	eMol27202832	−6.1	3128	eMol28321918	−6.1	3179	eMol27202482	−6.1
3078	eMol27490629	−6.1	3129	eMol3021957	−6.1	3180	eMol27202938	−6.1
3079	eMol3053706	−6.1	3130	eMol3264054	−6.1	3181	eMol3063576	−6.1
3080	eMol3400337	−6.1	3131	eMol36015426	−6.1	3182	eMol3091693	−6.1
3081	eMol36015978	−6.1	3132	eMol36015510	−6.1	3183	eMol3397107	−6.1
3082	eMol36016626	−6.1	3133	eMol36016666	−6.1	3184	eMol36015988	−6.1
3083	eMol43686132	−6.1	3134	eMol36734197	−6.1	3185	eMol36016416	−6.1
3084	eMol4477570	−6.1	3135	eMol36913018	−6.1	3186	eMol4694992	−6.1
3085	eMol45696249	−6.1	3136	eMol43752173	−6.1	3187	eMol5444391	−6.1
3086	eMol4573222	−6.1	3137	eMol43780890	−6.1	3188	eMol5444525	−6.1
3057	eMol7280788	−6.1	3108	eMol3315543	−6.1	3159	eMol43789927	−6.1

Table S1. *Continued.*

No.	Compound Name/Code	Docking Score (kcal/mol)	No.	Compound Name/Code	Docking Score (kcal/mol)	No.	Compound Name/Code	Docking Score (kcal/mol)
3189	eMol5444779	−6.1	3240	eMol20337436	−6.0	3291	eMol25784125	−6.0
3190	eMol5445019	−6.1	3241	eMol2126263	−6.0	3292	eMol26188103	−6.0
3191	eMol566446	−6.1	3242	eMol24109710	−6.0	3293	eMol27202954	−6.0
3192	eMol587772	−6.1	3243	eMol24484845	−6.0	3294	eMol31758530	−6.0
3193	eMol7288156	−6.1	3244	eMol24526125	−6.0	3295	eMol32245537	−6.0
3194	CHEMBL1572860	−6.1	3245	eMol25810047	−6.0	3296	eMol3316876	−6.0
3195	CHEMBL1736020	−6.1	3246	eMol26141668	−6.0	3297	eMol3423798	−6.0
3196	eMol11049916	−6.1	3247	eMol27187296	−6.0	3298	eMol36016292	−6.0
3197	eMol14071710	−6.1	3248	eMol313096295	−6.0	3299	eMol36636761	−6.0
3198	eMol17487545	−6.1	3249	eMol3457575	−6.0	3300	eMol43745160	−6.0
3199	eMol193274204	−6.1	3250	eMol36015690	−6.0	3301	eMol43757701	−6.0
3200	eMol2130267	−6.1	3251	eMol45560599	−6.0	3302	eMol45687695	−6.0
3201	eMol25784121	−6.1	3252	eMol5444609	−6.0	3303	eMol565260	−6.0
3202	eMol26244399	−6.1	3253	eMol6812204	−6.0	3304	eMol579976	−6.0
3203	eMol27202830	−6.1	3254	CHEMBL1320203	−6.0	3305	eMol6546367	−6.0
3204	eMol27490347	−6.1	3255	CHEMBL1492493	−6.0	3306	eMol7266946	−6.0
3205	eMol3048978	−6.1	3256	CHEMBL296457	−6.0	3307	CHEMBL1333010	−6.0
3206	eMol3318318	−6.1	3257	eMol101721903	−6.0	3308	CHEMBL1875720	−6.0
3207	eMol3460521	−6.1	3258	eMol11063247	−6.0	3309	CHEMBL3926487	−6.0
3208	eMol36015668	−6.1	3259	eMol11827681	−6.0	3310	eMol11920572	−6.0
3209	eMol36015710	−6.1	3260	eMol11908175	−6.0	3311	eMol11924298	−6.0
3210	eMol75947178	−6.1	3261	eMol18841974	−6.0	3312	eMol13536327	−6.0
3211	CHEMBL1390719	−6.0	3262	eMol24170571	−6.0	3313	eMol16378913	−6.0
3212	CHEMBL300718	−6.0	3263	eMol2498271	−6.0	3314	eMol16379715	−6.0
3213	eMol12034278	−6.0	3264	eMol25823463	−6.0	3315	eMol16379733	−6.0
3214	eMol13870199	−6.0	3265	eMol25881352	−6.0	3316	eMol19031541	−6.0
3215	eMol16374761	−6.0	3266	eMol27495723	−6.0	3317	eMol26339153	−6.0
3216	eMol16668429	−6.0	3267	eMol2992822	−6.0	3318	eMol27187016	−6.0
3217	eMol1684723	−6.0	3268	eMol32247779	−6.0	3319	eMol27202318	−6.0
3218	eMol17487345	−6.0	3269	eMol36015604	−6.0	3320	eMol27202480	−6.0
3219	eMol19041072	−6.0	3270	eMol3651370	−6.0	3321	eMol3460171	−6.0
3220	eMol19076610	−6.0	3271	eMol36898006	−6.0	3322	eMol3492428	−6.0
3221	eMol19567048	−6.0	3272	eMol43744852	−6.0	3323	eMol36015722	−6.0
3222	eMol206907990	−6.0	3273	eMol43779180	−6.0	3324	eMol36015958	−6.0
3223	eMol22755694	−6.0	3274	eMol5777105	−6.0	3325	eMol4382848	−6.0
3224	eMol24004870	−6.0	3275	eMol579208	−6.0	3326	eMol4395800	−6.0
3225	eMol24113537	−6.0	3276	eMol7270508	−6.0	3327	eMol4693783	−6.0
3226	eMol25583051	−6.0	3277	eMol98536537	−6.0	3328	eMol5444503	−6.0
3227	eMol27182870	−6.0	3278	CHEMBL1475496	−6.0	3329	eMol935476	−6.0
3228	eMol27202840	−6.0	3279	CHEMBL1488729	−6.0	3330	CHEMBL3417626	−6.0
3229	eMol27202972	−6.0	3280	CHEMBL1536346	−6.0	3331	CHEMBL3444374	−6.0
3230	eMol33328676	−6.0	3281	CHEMBL43557	−6.0	3332	CHEMBL3806217	−6.0
3231	eMol3397089	−6.0	3282	eMol1149945	−6.0	3333	CHEMBL43877	−6.0
3232	eMol36010135	−6.0	3283	eMol1415846	−6.0	3334	eMol172296216	−6.0
3233	eMol36015936	−6.0	3284	eMol16378909	−6.0	3335	eMol206976528	−6.0
3234	eMol36733947	−6.0	3285	eMol16407976	−6.0	3336	eMol2212585	−6.0
3235	eMol43747061	−6.0	3286	eMol18831935	−6.0	3337	eMol2438554	−6.0
3236	eMol4383786	−6.0	3287	eMol18923614	−6.0	3338	eMol26141626	−6.0
3237	eMol46210774	−6.0	3288	eMol2232828	−6.0	3339	eMol27045489	−6.0
3238	eMol5444499	−6.0	3289	eMol23999017	−6.0	3340	eMol3277055	−6.0
3239	CHEMBL3417625	−6.0	3290	eMol24113623	−6.0	3341	eMol3351148	−6.0
3210	eMol75947178	−6.1	3261	eMol18841974	−6.0	3312	eMol13536327	−6.0

Table S1. *Continued.*

No.	Compound Name/Code	Docking Score (kcal/mol)	No.	Compound Name/Code	Docking Score (kcal/mol)	No.	Compound Name/Code	Docking Score (kcal/mol)
3342	eMol3395457	−6.0	3393	eMol2992524	−6.0	3444	eMol4544980	−6.0
3343	eMol3499188	−6.0	3394	eMol3111079	−6.0	3445	eMol4694293	−6.0
3344	eMol35670178	−6.0	3395	eMol3119032	−6.0	3446	eMol4711170	−6.0
3345	eMol36015970	−6.0	3396	eMol32245409	−6.0	3447	eMol5444385	−6.0
3346	eMol36016116	−6.0	3397	eMol32246319	−6.0	3448	eMol5444405	−6.0
3347	eMol36733931	−6.0	3398	eMol3254168	−6.0	3449	eMol5444553	−6.0
3348	eMol36733937	−6.0	3399	eMol33344239	−6.0	3450	eMol7276298	−6.0
3349	eMol36888153	−6.0	3400	eMol36016004	−6.0	3451	eMol7283516	−6.0
3350	eMol44073193	−6.0	3401	eMol36016280	−6.0	3452	eMol72989932	−6.0
3351	eMol5444923	−6.0	3402	eMol36016538	−6.0	3453	eMol8853953	−6.0
3352	eMol6546369	−6.0	3403	eMol560173	−6.0	3454	eMol97962232	−6.0
3353	eMol674701	−6.0	3404	eMol566772	−6.0	3455	eMol98985756	−6.0
3354	eMol936508	−6.0	3405	eMol7289978	−6.0	3456	CHEMBL1347075	−5.9
3355	CHEMBL85147	−6.0	3406	CHEMBL1391450	−6.0	3457	CHEMBL85119	−5.9
3356	eMol11071012	−6.0	3407	CHEMBL361653	−6.0	3458	eMol1201940	−5.9
3357	eMol11828091	−6.0	3408	CHEMBL3805543	−6.0	3459	eMol1749926	−5.9
3358	eMol16379193	−6.0	3409	CHEMBL3805810	−6.0	3460	eMol25938161	−5.9
3359	eMol16379649	−6.0	3410	CHEMBL46620	−6.0	3461	eMol26141630	−5.9
3360	eMol16379827	−6.0	3411	eMol11896654	−6.0	3462	eMol27202850	−5.9
3361	eMol16379865	−6.0	3412	eMol11939765	−6.0	3463	eMol3252276	−5.9
3362	eMol27490343	−6.0	3413	eMol1681159	−6.0	3464	eMol36015366	−5.9
3363	eMol29662423	−6.0	3414	eMol27045563	−6.0	3465	eMol36015634	−5.9
3364	eMol32247207	−6.0	3415	eMol27182530	−6.0	3466	eMol36015670	−5.9
3365	eMol3585513	−6.0	3416	eMol27186992	−6.0	3467	eMol36015736	−5.9
3366	eMol36015644	−6.0	3417	eMol27202822	−6.0	3468	eMol36016548	−5.9
3367	eMol36015744	−6.0	3418	eMol32014360	−6.0	3469	eMol3651372	−5.9
3368	eMol36016290	−6.0	3419	eMol3255018	−6.0	3470	eMol43716255	−5.9
3369	eMol36016638	−6.0	3420	eMol36015696	−6.0	3471	eMol5444409	−5.9
3370	eMol36619357	−6.0	3421	eMol36016016	−6.0	3472	eMol5444919	−5.9
3371	eMol44073265	−6.0	3422	eMol36016644	−6.0	3473	eMol5447791	−5.9
3372	eMol44131056	−6.0	3423	eMol43772250	−6.0	3474	eMol5538604	−5.9
3373	eMol4473164	−6.0	3424	eMol48938138	−6.0	3475	eMol5538882	−5.9
3374	eMol5444511	−6.0	3425	eMol5444323	−6.0	3476	eMol579138	−5.9
3375	eMol5444837	−6.0	3426	eMol5444513	−6.0	3477	eMol7263572	−5.9
3376	eMol5444857	−6.0	3427	eMol7277736	−6.0	3478	eMol7280576	−5.9
3377	eMol5538878	−6.0	3428	CHEMBL1482443	−6.0	3479	CHEMBL1327048	−5.9
3378	eMol6829772	−6.0	3429	eMol24428102	−6.0	3480	CHEMBL1453502	−5.9
3379	eMol7272446	−6.0	3430	eMol25814749	−6.0	3481	CHEMBL1462195	−5.9
3380	eMol1026757	−6.0	3431	eMol26241386	−6.0	3482	CHEMBL3417620	−5.9
3381	eMol1077066	−6.0	3432	eMol27182364	−6.0	3483	CHEMBL46368	−5.9
3382	eMol1198254	−6.0	3433	eMol27202432	−6.0	3484	eMol1448893	−5.9
3383	eMol16374763	−6.0	3434	eMol27490299	−6.0	3485	eMol16379635	−5.9
3384	eMol1878750	−6.0	3435	eMol27490305	−6.0	3486	eMol16379679	−5.9
3385	eMol2388279	−6.0	3436	eMol29272184	−6.0	3487	eMol16379795	−5.9
3386	eMol25797963	−6.0	3437	eMol3073582	−6.0	3488	eMol23982156	−5.9
3387	eMol25813429	−6.0	3438	eMol3461011	−6.0	3489	eMol25797945	−5.9
3388	eMol25814739	−6.0	3439	eMol36015992	−6.0	3490	eMol2709463	−5.9
3389	eMol26333881	−6.0	3440	eMol36898040	−6.0	3491	eMol27202860	−5.9
3390	eMol27045581	−6.0	3441	eMol43757699	−6.0	3492	eMol29274292	−5.9
3391	eMol27202534	−6.0	3442	eMol43771188	−6.0	3493	eMol30117396	−5.9
3392	eMol27202944	−6.0	3443	eMol44012299	−6.0	3494	eMol30308489	−5.9
3363	eMol29662423	−6.0	3414	eMol27045563	−6.0	3465	eMol36015634	−5.9

Table S1. *Continued.*

No.	Compound Name/Code	Docking Score (kcal/mol)	No.	Compound Name/Code	Docking Score (kcal/mol)	No.	Compound Name/Code	Docking Score (kcal/mol)
3495	eMol31758354	−5.9	3546	eMol48996032	−5.9	3597	eMol2045101	−5.9
3496	eMol32245651	−5.9	3547	eMol5445039	−5.9	3598	eMol26141652	−5.9
3497	eMol3308467	−5.9	3548	eMol5527763	−5.9	3599	eMol2992478	−5.9
3498	eMol36016558	−5.9	3549	eMol560175	−5.9	3600	eMol3007749	−5.9
3499	eMol36016632	−5.9	3550	eMol7285010	−5.9	3601	eMol36010137	−5.9
3500	eMol36733927	−5.9	3551	CHEMBL1409464	−5.9	3602	eMol36010139	−5.9
3501	eMol43745052	−5.9	3552	CHEMBL1503062	−5.9	3603	eMol36016014	−5.9
3502	eMol43772256	−5.9	3553	CHEMBL3417622	−5.9	3604	eMol43420012	−5.9
3503	eMol43775420	−5.9	3554	CHEMBL3417651	−5.9	3605	eMol43691638	−5.9
3504	eMol44519731	−5.9	3555	CHEMBL3627819	−5.9	3606	eMol4708865	−5.9
3505	eMol5444325	−5.9	3556	CHEMBL3805842	−5.9	3607	eMol5444505	−5.9
3506	eMol5444521	−5.9	3557	CHEMBL42254	−5.9	3608	eMol5444929	−5.9
3507	eMol5444937	−5.9	3558	eMol16375243	−5.9	3609	eMol8599848	−5.9
3508	eMol6798950	−5.9	3559	eMol16757822	−5.9	3610	eMol98035511	−5.9
3509	eMol7669354	−5.9	3560	eMol18832810	−5.9	3611	eMol98295135	−5.9
3510	eMol8858445	−5.9	3561	eMol2045239	−5.9	3612	CHEMBL116635	−5.9
3511	CHEMBL1368478	−5.9	3562	eMol206984662	−5.9	3613	CHEMBL1480909	−5.9
3512	CHEMBL1497366	−5.9	3563	eMol24102384	−5.9	3614	CHEMBL1570569	−5.9
3513	eMol16056937	−5.9	3564	eMol27202524	−5.9	3615	CHEMBL184700	−5.9
3514	eMol19076760	−5.9	3565	eMol3061940	−5.9	3616	CHEMBL3806223	−5.9
3515	eMol206993232	−5.9	3566	eMol3525797	−5.9	3617	eMol11897152	−5.9
3516	eMol24110312	−5.9	3567	eMol36015428	−5.9	3618	eMol16351881	−5.9
3517	eMol25816331	−5.9	3568	eMol36016012	−5.9	3619	eMol16374603	−5.9
3518	eMol27187010	−5.9	3569	eMol36016152	−5.9	3620	eMol19076762	−5.9
3519	eMol27202218	−5.9	3570	eMol43731528	−5.9	3621	eMol206977250	−5.9
3520	eMol27202946	−5.9	3571	eMol43745064	−5.9	3622	eMol27115416	−5.9
3521	eMol30083536	−5.9	3572	eMol48982458	−5.9	3623	eMol27187292	−5.9
3522	eMol3245586	−5.9	3573	eMol581336	−5.9	3624	eMol27202396	−5.9
3523	eMol3379409	−5.9	3574	eMol679602	−5.9	3625	eMol32247059	−5.9
3524	eMol36015702	−5.9	3575	eMol76020498	−5.9	3626	eMol34126722	−5.9
3525	eMol36016164	−5.9	3576	eMol956537	−5.9	3627	eMol36015618	−5.9
3526	eMol36016634	−5.9	3577	eMol98451737	−5.9	3628	eMol36015938	−5.9
3527	eMol36016662	−5.9	3578	CHEMBL3251278	−5.9	3629	eMol36015990	−5.9
3528	eMol43745897	−5.9	3579	CHEMBL3303662	−5.9	3630	eMol36016532	−5.9
3529	eMol5444915	−5.9	3580	CHEMBL3417623	−5.9	3631	eMol5538886	−5.9
3530	eMol6770480	−5.9	3581	CHEMBL3417653	−5.9	3632	eMol5747647	−5.9
3531	eMol6844944	−5.9	3582	CHEMBL52027	−5.9	3633	eMol7276176	−5.9
3532	eMol7276886	−5.9	3583	eMol206962509	−5.9	3634	CHEMBL1416395	−5.9
3533	eMol7294930	−5.9	3584	eMol27202438	−5.9	3635	CHEMBL1529015	−5.9
3534	CHEMBL1559259	−5.9	3585	eMol32246641	−5.9	3636	CHEMBL3417619	−5.9
3535	eMol11905852	−5.9	3586	eMol34147555	−5.9	3637	CHEMBL3818256	−5.9
3536	eMol16378915	−5.9	3587	eMol5444389	−5.9	3638	eMol20351100	−5.9
3537	eMol27490283	−5.9	3588	eMol5538744	−5.9	3639	eMol2123188	−5.9
3538	eMol277684421	−5.9	3589	eMol685354	−5.9	3640	eMol2194421	−5.9
3539	eMol3120283	−5.9	3590	CHEMBL1323430	−5.9	3641	eMol25895035	−5.9
3540	eMol32417568	−5.9	3591	CHEMBL42492	−5.9	3642	eMol27202376	−5.9
3541	eMol3484149	−5.9	3592	CHEMBL50183	−5.9	3643	eMol27202820	−5.9
3542	eMol36015388	−5.9	3593	eMol101338171	−5.9	3644	eMol2992538	−5.9
3543	eMol3628332	−5.9	3594	eMol16379655	−5.9	3645	eMol32551016	−5.9
3544	eMol43779182	−5.9	3595	eMol16755860	−5.9	3646	eMol36010119	−5.9
3545	eMol43943035	−5.9	3596	eMol17182671	−5.9	3647	eMol36015538	−5.9

Table S1. *Continued.*

No.	Compound Name/Code	Docking Score (kcal/mol)	No.	Compound Name/Code	Docking Score (kcal/mol)	No.	Compound Name/Code	Docking Score (kcal/mol)
3648	eMol36015946	−5.9	3699	eMol7273458	−5.8	3750	eMol16801545	−5.8
3649	eMol36016624	−5.9	3700	CHEMBL296471	−5.8	3751	eMol233065671	−5.8
3650	eMol4396702	−5.9	3701	CHEMBL3805423	−5.8	3752	eMol24102744	−5.8
3651	eMol5444965	−5.9	3702	eMol16375283	−5.8	3753	eMol25808001	−5.8
3652	eMol5445015	−5.9	3703	eMol16379191	−5.8	3754	eMol25810019	−5.8
3653	CHEMBL2180424	−5.9	3704	eMol179165020	−5.8	3755	eMol27045477	−5.8
3654	CHEMBL3417644	−5.9	3705	eMol27187196	−5.8	3756	eMol27186986	−5.8
3655	eMol11492617	−5.9	3706	eMol27202942	−5.8	3757	eMol27187058	−5.8
3656	eMol11651355	−5.9	3707	eMol29272178	−5.8	3758	eMol30499969	−5.8
3657	eMol13457575	−5.9	3708	eMol3015060	−5.8	3759	eMol32246797	−5.8
3658	eMol1991716	−5.9	3709	eMol36015446	−5.8	3760	eMol32259086	−5.8
3659	eMol2234330	−5.9	3710	eMol36016136	−5.8	3761	eMol3247755	−5.8
3660	eMol24116061	−5.9	3711	eMol36016418	−5.8	3762	eMol3473706	−5.8
3661	eMol27045573	−5.9	3712	eMol36016576	−5.8	3763	eMol36015394	−5.8
3662	eMol27202826	−5.9	3713	eMol36016630	−5.8	3764	eMol36015628	−5.8
3663	eMol3060124	−5.9	3714	eMol43758409	−5.8	3765	eMol36733921	−5.8
3664	eMol35670746	−5.9	3715	eMol566748	−5.8	3766	eMol43779178	−5.8
3665	eMol36015662	−5.9	3716	eMol7297616	−5.8	3767	eMol4718807	−5.8
3666	eMol43749023	−5.9	3717	eMol8599285	−5.8	3768	eMol6844630	−5.8
3667	eMol45705052	−5.9	3718	CHEMBL1412503	−5.8	3769	eMol7265596	−5.8
3668	eMol5444781	−5.9	3719	CHEMBL1596292	−5.8	3770	eMol7280232	−5.8
3669	eMol5444941	−5.9	3720	CHEMBL3805156	−5.8	3771	CHEMBL1386169	−5.8
3670	eMol560295	−5.9	3721	eMol16351969	−5.8	3772	CHEMBL1612982	−5.8
3671	eMol7262416	−5.9	3722	eMol17485771	−5.8	3773	eMol11910455	−5.8
3672	eMol7275912	−5.9	3723	eMol2330945	−5.8	3774	eMol1201944	−5.8
3673	CHEMBL1349955	−5.8	3724	eMol25784525	−5.8	3775	eMol16379757	−5.8
3674	CHEMBL1551011	−5.8	3725	eMol27045549	−5.8	3776	eMol18837122	−5.8
3675	CHEMBL298186	−5.8	3726	eMol27202372	−5.8	3777	eMol2211247	−5.8
3676	CHEMBL36436	−5.8	3727	eMol27202464	−5.8	3778	eMol24102224	−5.8
3677	eMol16056895	−5.8	3728	eMol27202824	−5.8	3779	eMol25810025	−5.8
3678	eMol16351863	−5.8	3729	eMol279062638	−5.8	3780	eMol29946079	−5.8
3679	eMol16379727	−5.8	3730	eMol34126654	−5.8	3781	eMol29970678	−5.8
3680	eMol18567164	−5.8	3731	eMol36015418	−5.8	3782	eMol32246637	−5.8
3681	eMol18915564	−5.8	3732	eMol36888077	−5.8	3783	eMol3243276	−5.8
3682	eMol20337406	−5.8	3733	eMol3707337	−5.8	3784	eMol3378047	−5.8
3683	eMol27349471	−5.8	3734	eMol43686098	−5.8	3785	eMol36015404	−5.8
3684	eMol32245395	−5.8	3735	eMol4479471	−5.8	3786	eMol43692862	−5.8
3685	eMol34173242	−5.8	3736	eMol560051	−5.8	3787	eMol43757807	−5.8
3686	eMol3445274	−5.8	3737	eMol579892	−5.8	3788	CHEMBL1528422	−5.8
3687	eMol36015524	−5.8	3738	eMol5803186	−5.8	3789	CHEMBL2355657	−5.8
3688	eMol36016276	−5.8	3739	eMol8311949	−5.8	3790	eMol11949268	−5.8
3689	eMol36016286	−5.8	3740	eMol935358	−5.8	3791	eMol16385658	−5.8
3690	eMol36734015	−5.8	3741	ChEBI120532	−5.8	3792	eMol17487549	−5.8
3691	eMol44831971	−5.8	3742	CHEMBL1484192	−5.8	3793	eMol18899622	−5.8
3692	eMol5444441	−5.8	3743	CHEMBL1501308	−5.8	3794	eMol23736354	−5.8
3693	eMol5444873	−5.8	3744	CHEMBL1541968	−5.8	3795	eMol24062462	−5.8
3694	eMol54877800	−5.8	3745	CHEMBL44983	−5.8	3796	eMol25946128	−5.8
3695	eMol5538872	−5.8	3746	eMol1114360	−5.8	3797	eMol289941373	−5.8
3696	eMol5538874	−5.8	3747	eMol11942269	−5.8	3798	eMol29272180	−5.8
3697	eMol624279	−5.8	3748	eMol1346671	−5.8	3799	eMol3458277	−5.8
3698	eMol7268950	−5.8	3749	eMol1454694	−5.8	3800	eMol36015676	−5.8
3669	eMol5444941	−5.9	3720	CHEMBL3805156	−5.8	3771	CHEMBL1386169	−5.8

Table S1. *Continued.*

No.	Compound Name/Code	Docking Score (kcal/mol)	No.	Compound Name/Code	Docking Score (kcal/mol)	No.	Compound Name/Code	Docking Score (kcal/mol)
3801	eMol43757795	−5.8	3852	eMol3203801	−5.8	3903	eMol5444651	−5.7
3802	eMol4439491	−5.8	3853	eMol32867972	−5.8	3904	eMol5538230	−5.7
3803	eMol5444737	−5.8	3854	eMol3518209	−5.8	3905	eMol5538572	−5.7
3804	eMol5538750	−5.8	3855	eMol36015982	−5.8	3906	eMol578940	−5.7
3805	eMol11897144	−5.8	3856	eMol36016550	−5.8	3907	eMol8858379	−5.7
3806	eMol179165023	−5.8	3857	eMol36888147	−5.8	3908	eMol98677038	−5.7
3807	eMol18880257	−5.8	3858	eMol43224757	−5.8	3909	CHEMBL1430145	−5.7
3808	eMol23016524	−5.8	3859	eMol560067	−5.8	3910	eMol101341286	−5.7
3809	eMol24053582	−5.8	3860	eMol587732	−5.8	3911	eMol16378087	−5.7
3810	eMol26762435	−5.8	3861	eMol7290742	−5.8	3912	eMol1748573	−5.7
3811	eMol36015302	−5.8	3862	eMol1456137	−5.8	3913	eMol179164950	−5.7
3812	eMol36015768	−5.8	3863	eMol16351587	−5.8	3914	eMol24427988	−5.7
3813	eMol36015964	−5.8	3864	eMol16379197	−5.8	3915	eMol24538112	−5.7
3814	eMol42925185	−5.8	3865	eMol24428322	−5.8	3916	eMol2478254	−5.7
3815	eMol45932363	−5.8	3866	eMol25150648	−5.8	3917	eMol26273901	−5.7
3816	eMol5444833	−5.8	3867	eMol27115412	−5.8	3918	eMol3389097	−5.7
3817	eMol560053	−5.8	3868	eMol29947096	−5.8	3919	eMol3517782	−5.7
3818	CHEMBL1414275	−5.8	3869	eMol3118180	−5.8	3920	eMol36016636	−5.7
3819	CHEMBL46990	−5.8	3870	eMol32245479	−5.8	3921	eMol36733951	−5.7
3820	eMol1120516	−5.8	3871	eMol32246741	−5.8	3922	eMol43709145	−5.7
3821	eMol1149913	−5.8	3872	eMol3473526	−5.8	3923	eMol43754301	−5.7
3822	eMol16379631	−5.8	3873	eMol36015738	−5.8	3924	eMol43780754	−5.7
3823	eMol1684016	−5.8	3874	eMol36015818	−5.8	3925	eMol5444835	−5.7
3824	eMol177240154	−5.8	3875	eMol43777018	−5.8	3926	eMol5538900	−5.7
3825	eMol1996956	−5.8	3876	eMol44070271	−5.8	3927	eMol555458	−5.7
3826	eMol26291695	−5.8	3877	eMol5527727	−5.8	3928	eMol581338	−5.7
3827	eMol2708943	−5.8	3878	eMol5538566	−5.8	3929	eMol681870	−5.7
3828	eMol27485958	−5.8	3879	eMol7272094	−5.8	3930	CHEMBL1323102	−5.7
3829	eMol300748351	−5.8	3880	CHEMBL299758	−5.7	3931	CHEMBL3251274	−5.7
3830	eMol33328686	−5.8	3881	eMol1456572	−5.7	3932	CHEMBL3302804	−5.7
3831	eMol3377803	−5.8	3882	eMol15983238	−5.7	3933	CHEMBL3417656	−5.7
3832	eMol3550246	−5.8	3883	eMol16378895	−5.7	3934	CHEMBL3805990	−5.7
3833	eMol36015998	−5.8	3884	eMol29274406	−5.7	3935	eMol11905834	−5.7
3834	eMol36016402	−5.8	3885	eMol29945943	−5.7	3936	eMol1201942	−5.7
3835	eMol36016570	−5.8	3886	eMol29987856	−5.7	3937	eMol19834537	−5.7
3836	eMol36734157	−5.8	3887	eMol3063065	−5.7	3938	eMol2009845	−5.7
3837	eMol43706397	−5.8	3888	eMol316607523	−5.7	3939	eMol206994786	−5.7
3838	eMol4569646	−5.8	3889	eMol32246223	−5.7	3940	eMol24659537	−5.7
3839	eMol4769967	−5.8	3890	eMol33328746	−5.7	3941	eMol25017853	−5.7
3840	eMol581218	−5.8	3891	eMol3453673	−5.7	3942	eMol27202858	−5.7
3841	eMol7707303	−5.8	3892	eMol36016288	−5.7	3943	eMol276247962	−5.7
3842	CHEMBL295198	−5.8	3893	eMol36888099	−5.7	3944	eMol29574834	−5.7
3843	CHEMBL3805373	−5.8	3894	eMol7260540	−5.7	3945	eMol300994022	−5.7
3844	eMol16352275	−5.8	3895	CHEMBL1999927	−5.7	3946	eMol32245663	−5.7
3845	eMol16375907	−5.8	3896	CHEMBL3417647	−5.7	3947	eMol36010097	−5.7
3846	eMol16379189	−5.8	3897	eMol1129158	−5.7	3948	eMol36016430	−5.7
3847	eMol1754894	−5.8	3898	eMol16375745	−5.7	3949	eMol36016530	−5.7
3848	eMol19874354	−5.8	3899	eMol27187224	−5.7	3950	eMol42728099	−5.7
3849	eMol2490920	−5.8	3900	eMol27202940	−5.7	3951	eMol43678570	−5.7
3850	eMol27115418	−5.8	3901	eMol3165472	−5.7	3952	eMol43747049	−5.7
3851	eMol3091717	−5.8	3902	eMol36015334	−5.7	3953	eMol45908565	−5.7
3822	eMol16379631	−5.8	3873	eMol36015738	−5.8	3924	eMol43780754	−5.7

Table S1. *Continued.*

No.	Compound Name/Code	Docking Score (kcal/mol)	No.	Compound Name/Code	Docking Score (kcal/mol)	No.	Compound Name/Code	Docking Score (kcal/mol)
3954	CHEMBL1512259	−5.7	4005	eMol36015836	−5.7	4056	eMol1266027	−5.7
3955	eMol11980724	−5.7	4006	eMol36015850	−5.7	4057	eMol15983254	−5.7
3956	eMol179165017	−5.7	4007	eMol36016436	−5.7	4058	eMol16351885	−5.7
3957	eMol179330987	−5.7	4008	eMol44059490	−5.7	4059	eMol24080844	−5.7
3958	eMol2405173	−5.7	4009	eMol45789241	−5.7	4060	eMol24147635	−5.7
3959	eMol257850519	−5.7	4010	eMol5444653	−5.7	4061	eMol2709017	−5.7
3960	eMol30682572	−5.7	4011	eMol5444731	−5.7	4062	eMol27182526	−5.7
3961	eMol31873562	−5.7	4012	CHEMBL37887	−5.7	4063	eMol29272194	−5.7
3962	eMol3191734	−5.7	4013	CHEMBL3805719	−5.7	4064	eMol29956351	−5.7
3963	eMol4124876	−5.7	4014	CHEMBL42510	−5.7	4065	eMol32089964	−5.7
3964	eMol4492362	−5.7	4015	CHEMBL44909	−5.7	4066	eMol3315429	−5.7
3965	eMol5444793	−5.7	4016	eMol13870135	−5.7	4067	eMol3402145	−5.7
3966	eMol7293790	−5.7	4017	eMol16379739	−5.7	4068	eMol34147575	−5.7
3967	eMol98035508	−5.7	4018	eMol18936964	−5.7	4069	eMol3581283	−5.7
3968	CHEMBL1413103	−5.7	4019	eMol25825133	−5.7	4070	eMol3581435	−5.7
3969	CHEMBL1443164	−5.7	4020	eMol26264984	−5.7	4071	eMol36015408	−5.7
3970	CHEMBL1701550	−5.7	4021	eMol29963253	−5.7	4072	eMol36015966	−5.7
3971	CHEMBL3251265	−5.7	4022	eMol32245227	−5.7	4073	eMol36016002	−5.7
3972	CHEMBL3303952	−5.7	4023	eMol32246219	−5.7	4074	eMol36016272	−5.7
3973	CHEMBL3962862	−5.7	4024	eMol3314151	−5.7	4075	eMol36016600	−5.7
3974	eMol16379537	−5.7	4025	eMol3450783	−5.7	4076	eMol5538560	−5.7
3975	eMol16776762	−5.7	4026	eMol36015858	−5.7	4077	eMol679756	−5.7
3976	eMol206992985	−5.7	4027	eMol36016000	−5.7	4078	CHEMBL3417631	−5.6
3977	eMol24423982	−5.7	4028	eMol36016006	−5.7	4079	eMol13396536	−5.6
3978	eMol25881320	−5.7	4029	eMol36016420	−5.7	4080	eMol19269633	−5.6
3979	eMol2666505	−5.7	4030	eMol36016616	−5.7	4081	eMol24113033	−5.6
3980	eMol2881396	−5.7	4031	eMol36888165	−5.7	4082	eMol2588259	−5.6
3981	eMol31758390	−5.7	4032	eMol43747057	−5.7	4083	eMol26132226	−5.6
3982	eMol32245285	−5.7	4033	eMol43751241	−5.7	4084	eMol300090481	−5.6
3983	eMol3246420	−5.7	4034	eMol43754319	−5.7	4085	eMol36016404	−5.6
3984	eMol32738498	−5.7	4035	eMol46563820	−5.7	4086	eMol36016426	−5.6
3985	eMol3323420	−5.7	4036	eMol581354	−5.7	4087	eMol43731600	−5.6
3986	eMol3581639	−5.7	4037	CHEMBL1408481	−5.7	4088	eMol5444643	−5.6
3987	eMol50316116	−5.7	4038	CHEMBL413095	−5.7	4089	eMol5444921	−5.6
3988	eMol5538600	−5.7	4039	eMol16379791	−5.7	4090	eMol587912	−5.6
3989	eMol5538748	−5.7	4040	eMol1764260	−5.7	4091	eMol681846	−5.6
3990	eMol5803268	−5.7	4041	eMol2005049	−5.7	4092	eMol6844632	−5.6
3991	eMol6546341	−5.7	4042	eMol3059233	−5.7	4093	CHEMBL1299662	−5.6
3992	eMol670804	−5.7	4043	eMol3165424	−5.7	4094	CHEMBL3417624	−5.6
3993	eMol678803	−5.7	4044	eMol32245281	−5.7	4095	CHEMBL45997	−5.6
3994	eMol101338165	−5.7	4045	eMol36015704	−5.7	4096	eMol16351805	−5.6
3995	eMol11383493	−5.7	4046	eMol36015852	−5.7	4097	eMol18567168	−5.6
3996	eMol11689326	−5.7	4047	eMol36733943	−5.7	4098	eMol24111627	−5.6
3997	eMol14012794	−5.7	4048	eMol3707309	−5.7	4099	eMol24428014	−5.6
3998	eMol16379805	−5.7	4049	eMol43692886	−5.7	4100	eMol27452042	−5.6
3999	eMol23989036	−5.7	4050	eMol5444927	−5.7	4101	eMol27485950	−5.6
4000	eMol26287348	−5.7	4051	eMol565392	−5.7	4102	eMol2961107	−5.6
4001	eMol27115428	−5.7	4052	eMol579530	−5.7	4103	eMol3063476	−5.6
4002	eMol27187212	−5.7	4053	CHEMBL289053	−5.7	4104	eMol3222998	−5.6
4003	eMol3154639	−5.7	4054	CHEMBL398633	−5.7	4105	eMol32247631	−5.6
4004	eMol36015698	−5.7	4055	eMol1098662	−5.7	4106	eMol3652708	−5.6
3975	eMol16776762	−5.7	4026	eMol36015858	−5.7	4077	eMol679756	−5.7

Table S1. *Continued.*

No.	Compound Name/Code	Docking Score (kcal/mol)	No.	Compound Name/Code	Docking Score (kcal/mol)	No.	Compound Name/Code	Docking Score (kcal/mol)
4107	eMol5444825	−5.6	4158	eMol5444741	−5.6	4209	eMol34117146	−5.6
4108	eMol5538422	−5.6	4159	eMol5444821	−5.6	4210	eMol36015370	−5.6
4109	eMol7276046	−5.6	4160	eMol7262778	−5.6	4211	eMol36016628	−5.6
4110	eMol7720322	−5.6	4161	eMol936680	−5.6	4212	eMol36734039	−5.6
4111	eMol8227994	−5.6	4162	eMol11067809	−5.6	4213	eMol43716279	−5.6
4112	eMol99154804	−5.6	4163	eMol13406955	−5.6	4214	eMol44027807	−5.6
4113	CHEMBL1363412	−5.6	4164	eMol13856757	−5.6	4215	eMol5538274	−5.6
4114	CHEMBL185819	−5.6	4165	eMol15983296	−5.6	4216	eMol5538596	−5.6
4115	CHEMBL298877	−5.6	4166	eMol16378849	−5.6	4217	eMol561929	−5.6
4116	CHEMBL3805394	−5.6	4167	eMol16387280	−5.6	4218	eMol7297548	−5.6
4117	eMol1035451	−5.6	4168	eMol3118142	−5.6	4219	CHEMBL1361934	−5.6
4118	eMol16374525	−5.6	4169	eMol3471424	−5.6	4220	CHEMBL1409728	−5.6
4119	eMol17177598	−5.6	4170	eMol3531069	−5.6	4221	CHEMBL1502873	−5.6
4120	eMol23985110	−5.6	4171	eMol36015660	−5.6	4222	CHEMBL287739	−5.6
4121	eMol34103732	−5.6	4172	eMol36016568	−5.6	4223	CHEMBL46580	−5.6
4122	eMol3554360	−5.6	4173	eMol5538442	−5.6	4224	eMol11071094	−5.6
4123	eMol36015348	−5.6	4174	eMol562339	−5.6	4225	eMol16374967	−5.6
4124	eMol36015412	−5.6	4175	CHEMBL1333939	−5.6	4226	eMol1755498	−5.6
4125	eMol36015666	−5.6	4176	eMol15961299	−5.6	4227	eMol1996958	−5.6
4126	eMol36016406	−5.6	4177	eMol16387194	−5.6	4228	eMol2312222	−5.6
4127	eMol36016414	−5.6	4178	eMol3091455	−5.6	4229	eMol25936931	−5.6
4128	eMol5476123	−5.6	4179	eMol36016010	−5.6	4230	eMol3091595	−5.6
4129	eMol5538734	−5.6	4180	eMol36016434	−5.6	4231	eMol3165452	−5.6
4130	eMol579888	−5.6	4181	eMol36016536	−5.6	4232	eMol3247843	−5.6
4131	eMol581332	−5.6	4182	eMol53746272	−5.6	4233	eMol3252590	−5.6
4132	eMol98984071	−5.6	4183	eMol5444841	−5.6	4234	eMol36015332	−5.6
4133	CHEMBL46221	−5.6	4184	eMol561465	−5.6	4235	eMol36015708	−5.6
4134	eMol100505980	−5.6	4185	CHEMBL277367	−5.6	4236	eMol36016660	−5.6
4135	eMol11772107	−5.6	4186	eMol11665251	−5.6	4237	eMol565358	−5.6
4136	eMol1411602	−5.6	4187	eMol11857514	−5.6	4238	eMol7274020	−5.6
4137	eMol16351787	−5.6	4188	eMol16379199	−5.6	4239	eMol7289486	−5.6
4138	eMol1681157	−5.6	4189	eMol23169915	−5.6	4240	CHEMBL1310951	−5.5
4139	eMol24512694	−5.6	4190	eMol27490353	−5.6	4241	CHEMBL3192694	−5.5
4140	eMol27182632	−5.6	4191	eMol2992804	−5.6	4242	eMol11996719	−5.5
4141	eMol30248265	−5.6	4192	eMol3192660	−5.6	4243	eMol16715583	−5.5
4142	eMol36015996	−5.6	4193	eMol36015824	−5.6	4244	eMol179164999	−5.5
4143	eMol36016400	−5.6	4194	eMol36015868	−5.6	4245	eMol207218450	−5.5
4144	eMol6120463	−5.6	4195	eMol36016048	−5.6	4246	eMol24000041	−5.5
4145	eMol7261208	−5.6	4196	eMol5444555	−5.6	4247	eMol24659823	−5.5
4146	eMol11939349	−5.6	4197	eMol5444745	−5.6	4248	eMol25948294	−5.5
4147	eMol16379137	−5.6	4198	eMol5444823	−5.6	4249	eMol26236444	−5.5
4148	eMol177226996	−5.6	4199	eMol588330	−5.6	4250	eMol300233846	−5.5
4149	eMol18917833	−5.6	4200	eMol935616	−5.6	4251	eMol3394121	−5.5
4150	eMol189971515	−5.6	4201	eMol98750076	−5.6	4252	eMol36016028	−5.5
4151	eMol2277798	−5.6	4202	CHEMBL1538079	−5.6	4253	eMol36016620	−5.5
4152	eMol24659539	−5.6	4203	eMol1125948	−5.6	4254	eMol36888107	−5.5
4153	eMol2888718	−5.6	4204	eMol11942097	−5.6	4255	eMol43753923	−5.5
4154	eMol31758306	−5.6	4205	eMol16379641	−5.6	4256	eMol4495299	−5.5
4155	eMol36015682	−5.6	4206	eMol24107019	−5.6	4257	CHEMBL37758	−5.5
4156	eMol36016284	−5.6	4207	eMol3320388	−5.6	4258	eMol1259454	−5.5
4157	eMol43747097	−5.6	4208	eMol3375755	−5.6	4259	eMol13444090	−5.5
4128	eMol5476123	−5.6	4179	eMol36016010	−5.6	4230	eMol3091595	−5.6

Table S1. *Continued.*

No.	Compound Name/Code	Docking Score (kcal/mol)	No.	Compound Name/Code	Docking Score (kcal/mol)	No.	Compound Name/Code	Docking Score (kcal/mol)
4260	eMol16656843	−5.5	4311	eMol5538580	−5.5	4362	CHEMBL108678	−5.5
4261	eMol16786048	−5.5	4312	eMol5829456	−5.5	4363	eMol11922227	−5.5
4262	eMol24117398	−5.5	4313	eMol11900836	−5.5	4364	eMol16379571	−5.5
4263	eMol26298847	−5.5	4314	eMol13464851	−5.5	4365	eMol2466422	−5.5
4264	eMol27045473	−5.5	4315	eMol16274923	−5.5	4366	eMol30662258	−5.5
4265	eMol27485926	−5.5	4316	eMol17485889	−5.5	4367	eMol32245953	−5.5
4266	eMol2991951	−5.5	4317	eMol1753662	−5.5	4368	eMol33328728	−5.5
4267	eMol3105499	−5.5	4318	eMol2399769	−5.5	4369	eMol36015664	−5.5
4268	eMol49047598	−5.5	4319	eMol27115434	−5.5	4370	eMol36016428	−5.5
4269	eMol6514227	−5.5	4320	eMol30576034	−5.5	4371	eMol36016432	−5.5
4270	eMol7291792	−5.5	4321	eMol679702	−5.5	4372	eMol36016556	−5.5
4271	CHEMBL1994932	−5.5	4322	eMol7261308	−5.5	4373	eMol43745168	−5.5
4272	CHEMBL2238498	−5.5	4323	eMol7281506	−5.5	4374	eMol43757693	−5.5
4273	CHEMBL37829	−5.5	4324	eMol7639977	−5.5	4375	eMol5444867	−5.5
4274	eMol16351803	−5.5	4325	CHEMBL276947	−5.5	4376	eMol7285922	−5.5
4275	eMol24115063	−5.5	4326	CHEMBL3251273	−5.5	4377	CHEMBL1583040	−5.5
4276	eMol317058156	−5.5	4327	CHEMBL3304410	−5.5	4378	eMol1764760	−5.5
4277	eMol31758520	−5.5	4328	eMol13542776	−5.5	4379	eMol25621652	−5.5
4278	eMol32480666	−5.5	4329	eMol26236690	−5.5	4380	eMol2992506	−5.5
4279	eMol36015612	−5.5	4330	eMol2992800	−5.5	4381	eMol36015382	−5.5
4280	eMol36015686	−5.5	4331	eMol3247471	−5.5	4382	eMol36015720	−5.5
4281	eMol36016408	−5.5	4332	eMol3385015	−5.5	4383	eMol43754663	−5.5
4282	eMol5538598	−5.5	4333	eMol36015374	−5.5	4384	eMol5444327	−5.5
4283	eMol560297	−5.5	4334	eMol4260980	−5.5	4385	eMol579954	−5.5
4284	eMol6514345	−5.5	4335	eMol43745020	−5.5	4386	eMol587978	−5.5
4285	eMol695171	−5.5	4336	eMol5444341	−5.5	4387	eMol7271458	−5.5
4286	CHEMBL1524621	−5.5	4337	eMol5444625	−5.5	4388	eMol8267093	−5.5
4287	CHEMBL3954127	−5.5	4338	eMol5444785	−5.5	4389	CHEMBL283373	−5.4
4288	eMol179170445	−5.5	4339	eMol6810339	−5.5	4390	CHEMBL287914	−5.4
4289	eMol27202924	−5.5	4340	eMol7262724	−5.5	4391	CHEMBL3908563	−5.4
4290	eMol36624923	−5.5	4341	CHEMBL3251272	−5.5	4392	CHEMBL43067	−5.4
4291	eMol43692860	−5.5	4342	CHEMBL3302444	−5.5	4393	eMol2212509	−5.4
4292	eMol5538490	−5.5	4343	eMol11936155	−5.5	4394	eMol23982998	−5.4
4293	eMol5538498	−5.5	4344	eMol13998590	−5.5	4395	eMol33345106	−5.4
4294	eMol5538876	−5.5	4345	eMol1764882	−5.5	4396	eMol36898042	−5.4
4295	eMol581300	−5.5	4346	eMol23625944	−5.5	4397	eMol5444787	−5.4
4296	eMol7277304	−5.5	4347	eMol24006861	−5.5	4398	CHEMBL1363639	−5.4
4297	CHEMBL277894	−5.5	4348	eMol24432113	−5.5	4399	eMol13518872	−5.4
4298	eMol1120542	−5.5	4349	eMol29944972	−5.5	4400	eMol13924352	−5.4
4299	eMol16379651	−5.5	4350	eMol3594414	−5.5	4401	eMol16782365	−5.4
4300	eMol2462968	−5.5	4351	eMol36015376	−5.5	4402	eMol1749616	−5.4
4301	eMol2477726	−5.5	4352	eMol36015648	−5.5	4403	eMol24174701	−5.4
4302	eMol26295378	−5.5	4353	eMol36733923	−5.5	4404	eMol25656108	−5.4
4303	eMol27187222	−5.5	4354	eMol36733929	−5.5	4405	eMol27187262	−5.4
4304	eMol29274294	−5.5	4355	eMol43686126	−5.5	4406	eMol32247525	−5.4
4305	eMol31307202	−5.5	4356	eMol43716311	−5.5	4407	eMol32247621	−5.4
4306	eMol36015652	−5.5	4357	eMol43754317	−5.5	4408	eMol3252534	−5.4
4307	eMol36016274	−5.5	4358	eMol4566462	−5.5	4409	eMol36015678	−5.4
4308	eMol43222155	−5.5	4359	eMol5444755	−5.5	4410	eMol5444333	−5.4
4309	eMol43691644	−5.5	4360	eMol936264	−5.5	4411	eMol5444729	−5.4
4310	eMol5538280	−5.5	4361	eMol99058638	−5.5	4412	CHEMBL1582944	−5.4
4281	eMol36016408	−5.5	4332	eMol3385015	−5.5	4383	eMol43754663	−5.5

Table S1. *Continued.*

No.	Compound Name/Code	Docking Score (kcal/mol)	No.	Compound Name/Code	Docking Score (kcal/mol)	No.	Compound Name/Code	Docking Score (kcal/mol)
4413	CHEMBL1979224	−5.4	4464	eMol587792	−5.4	4515	eMol1125956	−5.3
4414	CHEMBL362906	−5.4	4465	eMol16757242	−5.4	4516	eMol27045257	−5.3
4415	eMol1684436	−5.4	4466	eMol17485765	−5.4	4517	eMol27045263	−5.3
4416	eMol1830750	−5.4	4467	eMol2221159	−5.4	4518	eMol3165074	−5.3
4417	eMol24145924	−5.4	4468	eMol23993395	−5.4	4519	eMol32246169	−5.3
4418	eMol26141646	−5.4	4469	eMol2493592	−5.4	4520	eMol3392027	−5.3
4419	eMol31653406	−5.4	4470	eMol3253524	−5.4	4521	eMol48982489	−5.3
4420	eMol31758546	−5.4	4471	eMol36015566	−5.4	4522	eMol5444437	−5.3
4421	eMol3232593	−5.4	4472	eMol36016606	−5.4	4523	eMol7273880	−5.3
4422	eMol3376557	−5.4	4473	eMol3652688	−5.4	4524	ChEBI114898	−5.3
4423	eMol36015354	−5.4	4474	eMol5538576	−5.4	4525	CHEMBL1499477	−5.3
4424	eMol36015750	−5.4	4475	eMol15267850	−5.4	4526	CHEMBL1902366	−5.3
4425	eMol36016658	−5.4	4476	eMol2992496	−5.4	4527	eMol16379689	−5.3
4426	eMol36016664	−5.4	4477	eMol36015638	−5.4	4528	eMol1682729	−5.3
4427	eMol3649917	−5.4	4478	eMol36015700	−5.4	4529	eMol17485891	−5.3
4428	eMol43746057	−5.4	4479	eMol36016038	−5.4	4530	eMol2022679	−5.3
4429	eMol4594606	−5.4	4480	eMol36016562	−5.4	4531	eMol2227456	−5.3
4430	eMol5527725	−5.4	4481	eMol36734195	−5.4	4532	eMol36015360	−5.3
4431	eMol5538492	−5.4	4482	eMol43744884	−5.4	4533	eMol36015932	−5.3
4432	eMol565570	−5.4	4483	eMol4423161	−5.4	4534	eMol36015980	−5.3
4433	eMol7290874	−5.4	4484	eMol7287028	−5.4	4535	eMol36016682	−5.3
4434	eMol8066827	−5.4	4485	CHEMBL1346627	−5.4	4536	eMol684044	−5.3
4435	CHEMBL2298707	−5.4	4486	CHEMBL1387694	−5.4	4537	CHEMBL3099880	−5.3
4436	eMol16370291	−5.4	4487	eMol1675470	−5.4	4538	eMol1237709	−5.3
4437	eMol24471123	−5.4	4488	eMol1683990	−5.4	4539	eMol16809465	−5.3
4438	eMol2588265	−5.4	4489	eMol17487547	−5.4	4540	eMol17485449	−5.3
4439	eMol277677811	−5.4	4490	eMol2597902	−5.4	4541	eMol18836616	−5.3
4440	eMol43368571	−5.4	4491	eMol27045547	−5.4	4542	eMol2010041	−5.3
4441	eMol5538278	−5.4	4492	eMol27485948	−5.4	4543	eMol3303379	−5.3
4442	eMol7297608	−5.4	4493	eMol3139546	−5.4	4544	eMol3521216	−5.3
4443	CHEMBL1573972	−5.4	4494	eMol31397858	−5.4	4545	eMol36015444	−5.3
4444	eMol11908173	−5.4	4495	eMol3393477	−5.4	4546	eMol4551419	−5.3
4445	eMol11943144	−5.4	4496	eMol36015834	−5.4	4547	eMol5444861	−5.3
4446	eMol17014756	−5.4	4497	eMol36016544	−5.4	4548	eMol5538554	−5.3
4447	eMol27187264	−5.4	4498	eMol36016560	−5.4	4549	eMol7279368	−5.3
4448	eMol2889626	−5.4	4499	eMol36016588	−5.4	4550	CHEMBL3402872	−5.3
4449	eMol2992808	−5.4	4500	CHEMBL3251275	−5.4	4551	eMol11935855	−5.3
4450	eMol3248581	−5.4	4501	CHEMBL3303239	−5.4	4552	eMol13496736	−5.3
4451	eMol36015194	−5.4	4502	eMol11070671	−5.4	4553	eMol32245321	−5.3
4452	eMol43754741	−5.4	4503	eMol16379435	−5.4	4554	eMol3239805	−5.3
4453	eMol15983236	−5.4	4504	eMol1993386	−5.4	4555	eMol579118	−5.3
4454	eMol27391996	−5.4	4505	eMol27372647	−5.4	4556	CHEMBL3443924	−5.3
4455	eMol2891570	−5.4	4506	eMol2992522	−5.4	4557	eMol2992834	−5.3
4456	eMol30576050	−5.4	4507	eMol43749015	−5.4	4558	eMol30081384	−5.3
4457	eMol31364087	−5.4	4508	eMol44058047	−5.4	4559	eMol36015362	−5.3
4458	eMol31758386	−5.4	4509	eMol6779880	−5.4	4560	eMol36015380	−5.3
4459	eMol3426910	−5.4	4510	eMol7259888	−5.4	4561	eMol36015828	−5.3
4460	eMol36015378	−5.4	4511	CHEMBL1196711	−5.3	4562	eMol43753953	−5.3
4461	eMol36015680	−5.4	4512	CHEMBL1424773	−5.3	4563	eMol936600	−5.3
4462	eMol36015830	−5.4	4513	CHEMBL557974	−5.3	4564	CHEMBL1452604	−5.3
4463	eMol43731224	−5.4	4514	eMol11070944	−5.3	4565	eMol27485924	−5.3
4434	eMol8066827	−5.4	4485	CHEMBL1346627	−5.4	4536	eMol684044	−5.3

Table S1. *Continued.*

No.	Compound Name/Code	Docking Score (kcal/mol)	No.	Compound Name/Code	Docking Score (kcal/mol)	No.	Compound Name/Code	Docking Score (kcal/mol)
4566	eMol34103616	−5.3	4617	eMol2002540	−5.2	4668	eMol3648258	−5.2
4567	eMol36016534	−5.3	4618	eMol20381014	−5.2	4669	eMol43747071	−5.2
4568	eMol3651748	−5.3	4619	eMol2992534	−5.2	4670	eMol560063	−5.2
4569	eMol5538602	−5.3	4620	eMol31758600	−5.2	4671	eMol587500	−5.2
4570	eMol566474	−5.3	4621	eMol32246589	−5.2	4672	eMol6844634	−5.2
4571	CHEMBL1325614	−5.3	4622	eMol36015440	−5.2	4673	CHEMBL1365141	−5.2
4572	CHEMBL3417629	−5.3	4623	eMol36016674	−5.2	4674	eMol11939763	−5.2
4573	eMol12034280	−5.3	4624	eMol36734167	−5.2	4675	eMol13857973	−5.2
4574	eMol13857885	−5.3	4625	eMol5538428	−5.2	4676	eMol16679965	−5.2
4575	eMol30739818	−5.3	4626	eMol5538740	−5.2	4677	eMol2265196	−5.2
4576	eMol33341133	−5.3	4627	eMol5538868	−5.2	4678	eMol2992510	−5.2
4577	eMol36016656	−5.3	4628	eMol679604	−5.2	4679	eMol3082150	−5.2
4578	eMol43686130	−5.3	4629	eMol936278	−5.2	4680	eMol3254728	−5.2
4579	eMol5444461	−5.3	4630	CHEMBL1495305	−5.2	4681	eMol36015816	−5.2
4580	eMol5444743	−5.3	4631	CHEMBL1525394	−5.2	4682	eMol36015860	−5.2
4581	eMol684462	−5.3	4632	eMol1372989	−5.2	4683	eMol44131054	−5.2
4582	eMol1415332	−5.3	4633	eMol15983294	−5.2	4684	eMol5445009	−5.2
4583	eMol2169394	−5.3	4634	eMol16385722	−5.2	4685	CHEMBL1336366	−5.2
4584	eMol27187412	−5.3	4635	eMol1676313	−5.2	4686	eMol16370277	−5.2
4585	eMol36015780	−5.3	4636	eMol17191180	−5.2	4687	eMol16390223	−5.2
4586	eMol4935076	−5.3	4637	eMol36016672	−5.2	4688	eMol3115949	−5.2
4587	eMol5538556	−5.3	4638	eMol36619339	−5.2	4689	eMol3331199	−5.2
4588	eMol5538702	−5.3	4639	eMol43747041	−5.2	4690	eMol36015728	−5.2
4589	eMol27490291	−5.3	4640	eMol4878607	−5.2	4691	eMol43789559	−5.2
4590	eMol3061651	−5.3	4641	eMol5444935	−5.2	4692	CHEMBL2297261	−5.2
4591	eMol36015352	−5.3	4642	eMol5538754	−5.2	4693	eMol1027541	−5.2
4592	eMol36015724	−5.3	4643	eMol9931089	−5.2	4694	eMol16379645	−5.2
4593	eMol36319682	−5.3	4644	eMol24113059	−5.2	4695	eMol16379767	−5.2
4594	eMol36733935	−5.3	4645	eMol32247521	−5.2	4696	eMol27485960	−5.2
4595	eMol43368191	−5.3	4646	eMol3775394	−5.2	4697	eMol3177115	−5.2
4596	eMol43781742	−5.3	4647	eMol43744984	−5.2	4698	eMol33751194	−5.2
4597	eMol5538282	−5.3	4648	eMol48997350	−5.2	4699	eMol36733949	−5.2
4598	eMol7278884	−5.3	4649	eMol5444629	−5.2	4700	eMol43013812	−5.2
4599	CHEMBL1421596	−5.3	4650	eMol100986457	−5.2	4701	eMol44458391	−5.2
4600	eMol16387248	−5.3	4651	eMol18832597	−5.2	4702	eMol579214	−5.2
4601	eMol16407996	−5.3	4652	eMol27492270	−5.2	4703	eMol13542782	−5.2
4602	eMol3037268	−5.3	4653	eMol3245754	−5.2	4704	eMol1454684	−5.2
4603	eMol3247263	−5.3	4654	eMol3331425	−5.2	4705	eMol24083372	−5.2
4604	eMol43744870	−5.3	4655	eMol36015622	−5.2	4706	eMol2459471	−5.2
4605	eMol5444749	−5.3	4656	eMol44089932	−5.2	4707	eMol3245382	−5.2
4606	eMol5538772	−5.3	4657	eMol4419462	−5.2	4708	eMol36015706	−5.2
4607	eMol1114358	−5.2	4658	eMol5538426	−5.2	4709	eMol5444395	−5.2
4608	eMol16379753	−5.2	4659	eMol7281382	−5.2	4710	eMol588046	−5.2
4609	eMol3118150	−5.2	4660	CHEMBL1503758	−5.2	4711	eMol7260062	−5.2
4610	eMol3418906	−5.2	4661	CHEMBL3932469	−5.2	4712	eMol7274732	−5.2
4611	eMol36015536	−5.2	4662	eMol11908179	−5.2	4713	eMol16379859	−5.1
4612	eMol36015994	−5.2	4663	eMol1674472	−5.2	4714	eMol1677693	−5.1
4613	eMol36016670	−5.2	4664	eMol1682797	−5.2	4715	eMol24100950	−5.1
4614	eMol7284830	−5.2	4665	eMol3001067	−5.2	4716	eMol5785086	−5.1
4615	eMol16390201	−5.2	4666	eMol31758466	−5.2	4717	eMol7282482	−5.1
4616	eMol1678749	−5.2	4667	eMol3176687	−5.2	4718	eMol98766494	−5.1
4587	eMol5538556	−5.3	4638	eMol36619339	−5.2	4689	eMol3331199	−5.2

Table S1. *Continued.*

No.	Compound Name/Code	Docking Score (kcal/mol)	No.	Compound Name/Code	Docking Score (kcal/mol)	No.	Compound Name/Code	Docking Score (kcal/mol)
4719	CHEMBL3942503	−5.1	4770	eMol36015624	−5.1	4821	eMol1035461	−5.0
4720	eMol115038	−5.1	4771	eMol43747053	−5.1	4822	eMol1448879	−5.0
4721	eMol18813395	−5.1	4772	eMol5538342	−5.1	4823	eMol179170454	−5.0
4722	eMol24657929	−5.1	4773	eMol624285	−5.1	4824	eMol29917277	−5.0
4723	eMol3380771	−5.1	4774	eMol13480687	−5.1	4825	eMol3523628	−5.0
4724	eMol36010363	−5.1	4775	eMol29917279	−5.1	4826	eMol5538888	−5.0
4725	eMol7291634	−5.1	4776	eMol3059181	−5.1	4827	eMol16379829	−5.0
4726	CHEMBL3251277	−5.1	4777	eMol49213685	−5.1	4828	eMol23996477	−5.0
4727	CHEMBL3302547	−5.1	4778	eMol5538756	−5.1	4829	eMol3032190	−5.0
4728	eMol15983244	−5.1	4779	CHEMBL43621	−5.1	4830	eMol3200820	−5.0
4729	eMol32246675	−5.1	4780	eMol11821373	−5.1	4831	eMol36016668	−5.0
4730	eMol3245798	−5.1	4781	eMol23999589	−5.1	4832	eMol48304474	−5.0
4731	eMol3523620	−5.1	4782	eMol2709467	−5.1	4833	eMol13512590	−5.0
4732	eMol3648256	−5.1	4783	eMol3249517	−5.1	4834	eMol29274280	−5.0
4733	eMol50319429	−5.1	4784	eMol34126726	−5.1	4835	eMol43754827	−5.0
4734	eMol5538276	−5.1	4785	eMol3480399	−5.1	4836	eMol44073147	−5.0
4735	eMol8455240	−5.1	4786	eMol3503186	−5.1	4837	eMol3493996	−5.0
4736	eMol1319937	−5.1	4787	eMol36015986	−5.1	4838	eMol36015336	−5.0
4737	eMol3048384	−5.1	4788	eMol43744964	−5.1	4839	eMol36015782	−5.0
4738	eMol32245889	−5.1	4789	eMol11864402	−5.0	4840	eMol3648252	−5.0
4739	eMol36016618	−5.1	4790	eMol3112215	−5.0	4841	eMol2409053	−5.0
4740	eMol43745176	−5.1	4791	eMol31758416	−5.0	4842	eMol27485956	−5.0
4741	eMol23999013	−5.1	4792	eMol32247467	−5.0	4843	eMol29917275	−5.0
4742	eMol2992486	−5.1	4793	eMol36016130	−5.0	4844	eMol33342600	−5.0
4743	eMol3214250	−5.1	4794	eMol43013800	−5.0	4845	eMol9959049	−5.0
4744	eMol32246429	−5.1	4795	eMol43743404	−5.0	4846	CHEMBL1543282	−4.9
4745	eMol3652086	−5.1	4796	eMol76019788	−5.0	4847	eMol1454660	−4.9
4746	eMol5444397	−5.1	4797	CHEMBL1976112	−5.0	4848	eMol34206915	−4.9
4747	eMol6833538	−5.1	4798	CHEMBL601797	−5.0	4849	eMol3658197	−4.9
4748	eMol7283790	−5.1	4799	eMol17014760	−5.0	4850	eMol29645389	−4.9
4749	CHEMBL1351050	−5.1	4800	eMol2001487	−5.0	4851	eMol43013872	−4.9
4750	eMol13520613	−5.1	4801	eMol3091469	−5.0	4852	eMol44080938	−4.9
4751	eMol31758260	−5.1	4802	eMol3376021	−5.0	4853	eMol5538486	−4.9
4752	eMol3252718	−5.1	4803	CHEMBL1388432	−5.0	4854	CHEMBL1386751	−4.9
4753	eMol3523624	−5.1	4804	eMol16385660	−5.0	4855	CHEMBL1701256	−4.9
4754	eMol43780908	−5.1	4805	eMol2889322	−5.0	4856	eMol17487551	−4.9
4755	eMol4422602	−5.1	4806	eMol2992528	−5.0	4857	eMol2881728	−4.9
4756	eMol4459106	−5.1	4807	eMol36015500	−5.0	4858	eMol32236794	−4.9
4757	eMol6833971	−5.1	4808	eMol36016528	−5.0	4859	eMol5444551	−4.9
4758	eMol13539183	−5.1	4809	eMol16379787	−5.0	4860	eMol15089032	−4.9
4759	eMol2167461	−5.1	4810	eMol31364085	−5.0	4861	eMol36898088	−4.9
4760	eMol3651756	−5.1	4811	eMol32246255	−5.0	4862	eMol43730460	−4.9
4761	eMol5538438	−5.1	4812	eMol43691630	−5.0	4863	eMol5444447	−4.9
4762	eMol5538742	−5.1	4813	eMol4904340	−5.0	4864	eMol5444733	−4.9
4763	eMol11465601	−5.1	4814	CHEMBL1500721	−5.0	4865	eMol587738	−4.9
4764	eMol16813507	−5.1	4815	CHEMBL3919247	−5.0	4866	CHEMBL3910321	−4.9
4765	eMol2264316	−5.1	4816	eMol11329944	−5.0	4867	eMol31653504	−4.9
4766	eMol316832342	−5.1	4817	eMol17014768	−5.0	4868	eMol32246579	−4.9
4767	eMol32246257	−5.1	4818	eMol18922892	−5.0	4869	eMol36015636	−4.9
4768	eMol33380364	−5.1	4819	eMol43746043	−5.0	4870	eMol36016146	−4.9
4769	eMol36015504	−5.1	4820	CHEMBL1452793	−5.0	4871	eMol50340675	−4.9
4740	eMol43745176	−5.1	4791	eMol31758416	−5.0	4842	eMol27485956	−5.0

Table S1. *Continued.*

No.	Compound Name/Code	Docking Score (kcal/mol)	No.	Compound Name/Code	Docking Score (kcal/mol)	No.	Compound Name/Code	Docking Score (kcal/mol)
4872	eMol5514743	−4.9	4915	eMol7276588	−4.8	4958	eMol2048663	−4.6
4873	eMol29942864	−4.9	4916	eMol36733899	−4.8	4959	eMol36015726	−4.6
4874	eMol36015626	−4.9	4917	CHEMBL1313582	−4.8	4960	eMol11952112	−4.6
4875	eMol36016008	−4.9	4918	eMol3048390	−4.8	4961	eMol16379883	−4.5
4876	eMol36887773	−4.9	4919	eMol5444751	−4.8	4962	eMol43691660	−4.5
4877	eMol43747101	−4.9	4920	eMol5538494	−4.8	4963	eMol5538884	−4.5
4878	eMol98058053	−4.9	4921	eMol5538738	−4.8	4964	eMol24006030	−4.5
4879	eMol36015732	−4.9	4922	eMol560365	−4.8	4965	eMol1676695	−4.5
4880	eMol43757853	−4.9	4923	CHEMBL1311188	−4.8	4966	eMol36015856	−4.5
4881	eMol11681118	−4.9	4924	eMol2992480	−4.8	4967	eMol8238421	−4.5
4882	eMol16379853	−4.9	4925	eMol1684765	−4.7	4968	eMol1178884	−4.5
4883	eMol2501901	−4.9	4926	eMol3145070	−4.7	4969	eMol27348310	−4.5
4884	eMol2992824	−4.9	4927	eMol3512950	−4.7	4970	eMol1266023	−4.5
4885	eMol32245269	−4.9	4928	eMol11747245	−4.7	4971	eMol5538892	−4.5
4886	eMol679700	−4.9	4929	eMol17014766	−4.7	4972	eMol32246633	−4.4
4887	eMol24110832	−4.8	4930	eMol16799937	−4.7	4973	eMol29274282	−4.4
4888	eMol31758564	−4.8	4931	eMol30288722	−4.7	4974	eMol43415245	−4.4
4889	eMol32245319	−4.8	4932	eMol32246207	−4.7	4975	eMol48312056	−4.4
4890	eMol32247457	−4.8	4933	eMol1454680	−4.7	4976	eMol5785084	−4.4
4891	eMol3419044	−4.8	4934	eMol16378089	−4.7	4977	eMol43746069	−4.4
4892	eMol5514745	−4.8	4935	eMol5444411	−4.7	4978	eMol30647010	−4.3
4902	eMol4448867	−4.8	4945	CHEMBL1406150	−4.6	4988	eMol17199912	−4.2
4903	eMol5444829	−4.8	4946	eMol1114366	−4.6	4989	CHEMBL3928151	−4.1
4904	eMol5444859	−4.8	4947	eMol16378097	−4.6	4990	CHEMBL1525899	−4.1
4905	eMol29274296	−4.8	4948	eMol3254458	−4.6	4991	eMol1471822	−4.1
4906	eMol5538890	−4.8	4949	eMol3473340	−4.6	4992	eMol32247517	−4.0
4907	eMol98068785	−4.8	4950	eMol3652896	−4.6	4993	CHEMBL3194843	−4.0
4908	eMol3257328	−4.8	4951	eMol5538910	−4.6	4994	eMol1023917	−4.0
4909	eMol3316910	−4.8	4952	eMol13469491	−4.6	4995	eMol207114346	−4.0
4910	eMol36016680	−4.8	4953	eMol27492328	−4.6	4996	eMol36015960	−4.0
4911	eMol3710762	−4.8	4954	eMol44548742	−4.6	4997	eMol5785216	−4.0
4912	eMol43759025	−4.8	4955	eMol5538894	−4.6	4998	eMol36731517	−3.9
4913	eMol5444435	−4.8	4956	eMol5444403	−4.6	4999	eMol13803787	−3.8
4914	eMol5538246	−4.8	4957	eMol43789551	−4.6	5000	eMol31758544	−3.7

[a] Duplicated compounds with identical InChKey could be observed.

Table S2. Estimated conventional and intermediate docking scores (in kcal/mol) for QNZ and the top 1000 potent allosteric inhibitors against mutated PBP2a allosteric site in acidic medium.[a]

No.	Compound Name/Code	Docking score (kcal/mol)		No.	Compound Name/Code	Docking score (kcal/mol)	
		Conventional	Intermediate			Conventional	Intermediate
	QNZ	−6.9	−8.0	50	eMol22447774	−8.5	−8.7
1	eMol19266609	−7.9	−9.5	51	eMol19076742	−8.6	−8.7
2	eMol24519694	−9.0	−9.5	52	CHEMBL1215650	−8.5	−8.7
3	eMol26313117	−8.9	−9.4	53	CHEMBL1579061	−8.5	−8.7
4	eMol24389393	−7.9	−9.4	54	eMol26269394	−8.5	−8.7
5	CHEMBL1214717	−9.4	−9.4	55	eMol31781482	−8.7	−8.6
6	eMol29587310	−9.4	−9.4	56	eMol23157132	−8.4	−8.6
7	eMol45585846	−9.4	−9.4	57	CHEMBL1215649	−8.5	−8.6
8	eMol23239806	−9.3	−9.4	58	eMol26196316	−8.5	−8.6
9	CHEMBL1214718	−9.2	−9.3	59	eMol30646140	−7.7	−8.6
10	eMol29597031	−9.2	−9.3	60	eMol72993476	−7.7	−8.6
11	eMol31786896	−8.4	−9.3	61	CHEMBL3818129	−8.5	−8.6
12	eMol45585848	−9.2	−9.3	62	eMol26319231	−8.2	−8.6
13	eMol23186178	−9.1	−9.3	63	eMol300994534	−8.4	−8.6
14	eMol2403964	−8.7	−9.3	64	eMol48938145	−8.4	−8.6
15	eMol19076714	−8.4	−9.2	65	CHEMBL1214713	−8.2	−8.6
16	eMol31297102	−7.9	−9.2	66	CHEMBL1384683	−8.2	−8.6
17	eMol26293960	−8.1	−9.2	67	eMol26270654	−8.2	−8.6
18	eMol19076718	−8.9	−9.1	68	eMol29633913	−7.8	−8.6
19	CHEMBL1534883	−9.0	−9.1	69	eMol33366267	−7.9	−8.6
20	eMol1344114	−9.0	−9.1	70	eMol45622743	−7.8	−8.6
21	eMol18542844	−9.1	−9.1	71	eMol75958504	−7.9	−8.6
22	CHEMBL3819187	−7.8	−9.1	72	eMol23246696	−8.5	−8.6
23	eMol30323633	−9.0	−9.1	73	CHEMBL1558437	−8.2	−8.6
24	CHEMBL1214794	−8.9	−9.0	74	eMol26242050	−8.1	−8.6
25	eMol26196290	−8.9	−9.0	75	eMol30671942	−7.9	−8.5
26	eMol45585824	−8.9	−9.0	76	eMol26242034	−8.2	−8.5
27	CHEMBL1214938	−8.7	−9.0	77	eMol26242042	−7.6	−8.5
28	eMol20375178	−8.7	−9.0	78	eMol23175530	−8.3	−8.5
29	eMol30323569	−8.6	−9.0	79	CHEMBL3805924	−8.3	−8.5
30	CHEMBL3818732	−8.0	−9.0	80	CHEMBL4103504	−8.3	−8.5
31	eMol30341049	−8.8	−8.9	81	eMol24428240	−7.2	−8.5
32	eMol43906045	−8.8	−8.9	82	eMol24519844	−8.4	−8.5
33	eMol26437582	−8.2	−8.9	83	eMol23181799	−8.3	−8.4
34	eMol19076712	−8.9	−8.9	84	eMol26242044	−8.3	−8.4
35	eMol20378769	−8.7	−8.9	85	eMol579568	−8.4	−8.4
36	CHEMBL3818584	−8.3	−8.9	86	eMol23239141	−8.3	−8.4
37	eMol3021959	−7.1	−8.9	87	CHEMBL1215080	−8.4	−8.4
38	eMol18542846	−8.8	−8.8	88	eMol23243407	−8.2	−8.4
39	eMol20342262	−7.9	−8.8	89	eMol43906059	−8.4	−8.4
40	eMol1166007	−8.7	−8.8	90	eMol587826	−8.2	−8.4
41	eMol26242018	−8.0	−8.8	91	eMol588192	−8.2	−8.4
42	eMol19076754	−8.6	−8.8	92	eMol25810077	−7.9	−8.4
43	eMol20337486	−8.8	−8.8	93	eMol27202240	−8.4	−8.4
44	eMol20379909	−8.8	−8.8	94	eMol679826	−8.3	−8.4
45	CHEMBL1215648	−8.6	−8.7	95	CHEMBL3818035	−7.9	−8.4
46	eMol26196326	−8.6	−8.7	96	eMol36912832	−7.9	−8.4
47	eMol45585842	−8.6	−8.7	97	eMol43906055	−8.0	−8.4
48	eMol23241028	−8.6	−8.7	98	eMol18962382	−8.4	−8.4
49	eMol23190351	−7.9	−8.7	99	eMol27202910	−8.3	−8.4

Table S2. *Continued.*

No.	Compound Name/Code	Docking score (kcal/mol)		No.	Compound Name/Code	Docking score (kcal/mol)	
		Conventional	Intermediate			Conventional	Intermediate
100	eMol31532412	−8.3	−8.4	152	eMol681936	−8.2	−8.2
101	eMol25810075	−8.3	−8.4	153	CHEMBL508966	−8.1	−8.2
102	eMol26264570	−8.1	−8.4	154	eMol1755402	−8.0	−8.2
103	eMol26313223	−8.3	−8.4	155	eMol24530483	−8.2	−8.2
104	eMol30248263	−8.0	−8.4	156	eMol26242038	−7.9	−8.2
105	CHEMBL1214647	−8.1	−8.4	157	eMol26320021	−7.4	−8.2
106	eMol679820	−7.2	−8.4	158	eMol566488	−8.1	−8.2
107	CHEMBL1215003	−8.2	−8.3	159	CHEMBL1215578	−8.0	−8.2
108	CHEMBL3817888	−8.1	−8.3	160	CHEMBL3818775	−8.2	−8.2
109	eMol193446288	−8.3	−8.3	161	eMol27252412	−8.1	−8.2
110	eMol27202248	−8.4	−8.3	162	eMol299980544	−8.2	−8.2
111	eMol43906053	−8.2	−8.3	163	eMol679822	−8.0	−8.2
112	CHEMBL3818572	−8.0	−8.3	164	eMol956623	−8.2	−8.2
113	CHEMBL395193	−8.3	−8.3	165	eMol11920256	−8.1	−8.2
114	eMol18542848	−8.3	−8.3	166	eMol23178762	−8.1	−8.2
115	eMol2992792	−8.1	−8.3	167	eMol48982653	−7.7	−8.2
116	eMol6263471	−8.2	−8.3	168	CHEMBL1215797	−8.0	−8.2
117	eMol26242022	−7.9	−8.3	169	eMol18550846	−8.1	−8.2
118	eMol43731476	−7.6	−8.3	170	eMol20377597	−8.0	−8.2
119	eMol46480173	−8.2	−8.3	171	eMol4566508	−7.3	−8.2
120	eMol19076598	−7.3	−8.3	172	eMol26314779	−8.1	−8.2
121	eMol20347873	−8.2	−8.3	173	eMol4558540	−8.0	−8.2
122	eMol206926236	−8.2	−8.3	174	CHEMBL3819481	−7.9	−8.2
123	eMol300094331	−8.2	−8.3	175	eMol20368046	−8.1	−8.2
124	eMol300154219	−7.3	−8.3	176	eMol23147497	−8.1	−8.2
125	eMol25810083	−8.3	−8.3	177	eMol26330545	−8.2	−8.2
126	eMol26242032	−7.9	−8.3	178	eMol29565259	−7.8	−8.2
127	eMol25810071	−8.3	−8.3	179	eMol45585838	−7.8	−8.2
128	eMol46365626	−8.2	−8.3	180	eMol679824	−8.0	−8.2
129	eMol566574	−8.1	−8.3	181	CHEMBL1214648	−8.0	−8.2
130	eMol27406062	−7.8	−8.3	182	CHEMBL1215004	−7.7	−8.2
131	CHEMBL1214935	−8.1	−8.3	183	eMol26242024	−7.7	−8.2
132	CHEMBL3818929	−8.2	−8.3	184	eMol26242026	−7.8	−8.2
133	CHEMBL518358	−8.2	−8.3	185	eMol26242052	−7.3	−8.2
134	eMol43906051	−8.1	−8.3	186	eMol29587306	−7.7	−8.2
135	CHEMBL1214936	−8.2	−8.3	187	eMol45585836	−7.7	−8.2
136	eMol26196312	−8.2	−8.3	188	eMol679556	−8.0	−8.2
137	eMol26314331	−7.5	−8.3	189	CHEMBL3582082	−7.3	−8.1
138	eMol30017880	−7.4	−8.3	190	eMol20361212	−8.1	−8.1
139	eMol45585832	−8.2	−8.3	191	eMol37002179	−7.4	−8.1
140	eMol25819217	−8.3	−8.2	192	eMol4562556	−8.1	−8.1
141	eMol26809774	−8.2	−8.2	193	eMol4578697	−7.3	−8.1
142	eMol27202258	−8.3	−8.2	194	eMol45935562	−7.5	−8.1
143	eMol46490873	−8.1	−8.2	195	eMol565850	−7.0	−8.1
144	eMol26314565	−8.2	−8.2	196	CHEMBL1601398	−8.0	−8.1
145	eMol1088331	−8.2	−8.2	197	CHEMBL3582080	−7.9	−8.1
146	eMol13856171	−8.0	−8.2	198	eMol2507718	−8.0	−8.1
147	eMol29653471	−8.2	−8.2	199	eMol256911679	−8.0	−8.1
148	eMol30323345	−9.8	−8.2	200	eMol27202348	−8.1	−8.1
149	eMol43731530	−7.3	−8.2	201	eMol301527162	−8.0	−8.1
150	eMol45622745	−8.2	−8.2	202	eMol4518648	−8.1	−8.1
151	eMol46480165	−8.2	−8.2	203	eMol4552519	−8.0	−8.1

Table S2. *Continued.*

No.	Compound Name/Code	Docking score (kcal/mol)		No.	Compound Name/Code	Docking score (kcal/mol)	
		Conventional	Intermediate			Conventional	Intermediate
204	eMol4446697	−7.7	−8.1	256	eMol25810089	−8.0	−8.0
205	eMol4577396	−8.0	−8.1	257	eMol26242040	−7.8	−8.0
206	eMol177199339	−7.9	−8.1	258	eMol26242048	−7.6	−8.0
207	eMol27202252	−8.0	−8.1	259	eMol26309353	−7.9	−8.0
208	eMol300994251	−7.9	−8.1	260	eMol4381378	−7.9	−8.0
209	eMol36912834	−8.0	−8.1	261	eMol4581546	−7.8	−8.0
210	eMol48768479	−7.9	−8.1	262	eMol50556474	−7.5	−8.0
211	eMol579756	−8.0	−8.1	263	CHEMBL2259942	−7.8	−8.0
212	eMol580072	−8.0	−8.1	264	CHEMBL3819363	−7.4	−8.0
213	eMol4566756	−7.9	−8.1	265	eMol26311024	−7.7	−8.0
214	eMol27202760	−8.0	−8.1	266	eMol43757885	−7.6	−8.0
215	eMol27202916	−7.2	−8.1	267	eMol679670	−8.0	−8.0
216	eMol29634797	−7.6	−8.1	268	CHEMBL1632342	−7.2	−8.0
217	CHEMBL1214941	−8.6	−8.1	269	eMol206910852	−7.9	−8.0
218	eMol25784531	−7.1	−8.1	270	eMol233046940	−7.7	−8.0
219	eMol27091674	−8.0	−8.1	271	eMol26313085	−7.5	−8.0
220	eMol679550	−7.9	−8.1	272	eMol46363574	−7.9	−8.0
221	CHEMBL1443074	−8.0	−8.1	273	eMol679552	−7.9	−8.0
222	eMol18940433	−8.0	−8.1	274	CHEMBL3818377	−8.4	−8.0
223	eMol27091498	−8.0	−8.1	275	eMol206911267	−7.9	−8.0
224	eMol20378767	−7.9	−8.1	276	eMol46480254	−7.9	−8.0
225	eMol27091442	−7.9	−8.1	277	eMol679664	−8.0	−8.0
226	eMol565798	−8.1	−8.1	278	eMol26263266	−7.0	−7.9
227	eMol29638777	−7.8	−8.1	280	eMol4579587	−7.7	−7.9
228	eMol4397966	−8.0	−8.1	281	eMol46210804	−7.6	−7.9
229	eMol45622749	−7.8	−8.1	282	eMol25908513	−7.9	−7.9
230	eMol579718	−8.0	−8.1	283	eMol27202994	−7.9	−7.9
231	eMol179164947	−7.8	−8.0	279	eMol4116278	−7.7	−7.9
232	eMol27202242	−8.0	−8.0	284	eMol4552457	−7.8	−7.9
233	eMol27202948	−7.4	−8.0	285	eMol565168	−8.6	−7.9
234	eMol18551342	−8.0	−8.0	286	eMol1035565	−7.9	−7.9
235	eMol206992517	−8.0	−8.0	287	eMol26259383	−7.3	−7.9
236	eMol25966646	−7.9	−8.0	288	eMol679554	−7.8	−7.9
237	eMol4549830	−7.3	−8.0	289	eMol26315830	−7.8	−7.9
238	CHEMBL3805841	−7.7	−8.0	290	eMol46480212	−7.8	−7.9
239	eMol1035521	−7.9	−8.0	291	eMol4726858	−7.6	−7.9
240	CHEMBL1214934	−7.0	−8.0	292	eMol566288	−7.8	−7.9
241	eMol26262168	−7.0	−8.0	293	eMol587628	−7.9	−7.9
242	eMol28321864	−8.0	−8.0	294	CHEMBL3817906	−7.7	−7.9
243	eMol681814	−7.9	−8.0	295	CHEMBL3819338	−7.6	−7.9
244	CHEMBL1215082	−7.4	−8.0	296	eMol24313582	−7.8	−7.9
245	CHEMBL485839	−8.0	−8.0	297	eMol26242046	−8.0	−7.9
246	eMol43906047	−7.9	−8.0	298	eMol27202958	−7.7	−7.9
247	eMol43906057	−7.4	−8.0	299	eMol27202992	−7.8	−7.9
248	eMol46367311	−7.8	−8.0	300	eMol4555657	−7.8	−7.9
249	eMol581308	−7.9	−8.0	301	eMol4572924	−7.8	−7.9
250	CHEMBL2259943	−7.9	−8.0	302	eMol56752349	−7.9	−7.9
251	eMol4581322	−8.0	−8.0	303	eMol587646	−7.5	−7.9
252	CHEMBL2259944	−8.0	−8.0	304	eMol43745241	−7.5	−7.9
253	eMol20342220	−7.8	−8.0	305	eMol4389171	−7.8	−7.9
254	eMol20342278	−7.9	−8.0	306	eMol579044	−7.4	−7.9
255	eMol25810001	−7.9	−8.0	307	CHEMBL1528362	−7.7	−7.9

Table S2. *Continued.*

No.	Compound Name/Code	Docking score (kcal/mol)		No.	Compound Name/Code	Docking score (kcal/mol)	
		Conventional	Intermediate			Conventional	Intermediate
308	eMol179164959	−7.9	−7.9	360	eMol4740224	−7.8	−7.8
309	eMol19076348	−7.7	−7.9	361	eMol588388	−7.6	−7.8
310	CHEMBL3819186	−7.8	−7.9	362	eMol561887	−7.8	−7.8
311	eMol26251042	−7.8	−7.9	363	eMol23202652	−7.8	−7.8
312	CHEMBL3819597	−7.8	−7.9	364	CHEMBL3818582	−7.1	−7.8
313	eMol26311238	−7.7	−7.9	365	eMol1429356	−7.3	−7.8
314	eMol4555647	−7.8	−7.9	366	eMol17203477	−7.5	−7.8
315	CHEMBL1212961	−7.8	−7.9	367	eMol19076364	−7.7	−7.8
316	eMol18550688	−7.8	−7.9	368	eMol46365712	−7.6	−7.8
317	eMol19267414	−7.8	−7.9	369	eMol566118	−7.5	−7.8
318	eMol193447787	−7.5	−7.9	370	eMol679668	−7.5	−7.8
319	eMol24029848	−7.5	−7.9	371	eMol8267101	−7.6	−7.8
320	eMol26242054	−7.4	−7.9	372	eMol26312078	−7.5	−7.8
321	eMol300136046	−7.8	−7.9	373	eMol300445092	−7.7	−7.8
322	eMol681860	−7.7	−7.9	374	eMol30665797	−7.7	−7.8
323	CHEMBL1215006	−7.3	−7.8	375	eMol43744848	−7.7	−7.8
324	eMol20342268	−7.7	−7.8	376	CHEMBL3817850	−7.7	−7.8
325	eMol24520354	−7.8	−7.8	377	eMol20342205	−7.7	−7.8
326	eMol27202956	−8.1	−7.8	378	eMol27202264	−7.7	−7.8
327	eMol299966748	−7.3	−7.8	379	eMol206985996	−7.7	−7.7
328	eMol314064301	−7.8	−7.8	380	eMol25965034	−7.7	−7.7
329	eMol50301465	−7.2	−7.8	381	eMol26310961	−7.7	−7.7
330	CHEMBL1215081	−7.9	−7.8	382	eMol27202268	−7.6	−7.7
331	CHEMBL1215803	−7.6	−7.8	383	eMol43906067	−7.5	−7.7
332	CHEMBL3819140	−7.6	−7.8	384	eMol25810163	−7.6	−7.7
333	eMol206970196	−7.8	−7.8	385	eMol3103759	−7.6	−7.7
334	eMol24525196	−7.7	−7.8	386	eMol58034831	−7.7	−7.7
335	eMol26196328	−7.9	−7.8	387	eMol581076	−7.4	−7.7
336	eMol4090808	−7.3	−7.8	388	eMol179165002	−7.7	−7.7
337	eMol45585844	−7.9	−7.8	389	eMol179165014	−7.7	−7.7
338	eMol4579154	−7.8	−7.8	390	eMol20337448	−7.2	−7.7
339	eMol4580802	−7.7	−7.8	391	eMol23249723	−7.7	−7.7
340	eMol562493	−7.7	−7.8	392	eMol3318312	−7.6	−7.7
341	eMol679666	−7.7	−7.8	393	eMol588272	−7.4	−7.7
342	CHEMBL1214861	−7.8	−7.8	394	eMol26141700	−7.7	−7.7
343	CHEMBL1371074	−7.7	−7.8	395	eMol27202980	−7.4	−7.7
344	eMol23208796	−7.2	−7.8	396	CHEMBL1214645	−7.3	−7.7
345	eMol24511821	−7.7	−7.8	397	CHEMBL517897	−7.7	−7.7
346	eMol25813417	−7.8	−7.8	398	eMol25955419	−7.7	−7.7
347	eMol27202742	−7.7	−7.8	399	eMol27202996	−7.3	−7.7
348	eMol566380	−7.9	−7.8	400	eMol7667302	−7.7	−7.7
349	CHEMBL323866	−7.6	−7.8	401	eMol935898	−7.2	−7.7
350	eMol208103615	−7.8	−7.8	402	CHEMBL485838	−7.6	−7.7
351	eMol26242036	−7.4	−7.8	403	eMol26189153	−7.4	−7.7
352	eMol35699388	−7.2	−7.8	404	eMol26356948	−7.4	−7.7
353	eMol35717814	−7.3	−7.8	405	eMol588168	−7.3	−7.7
354	eMol4413761	−7.7	−7.8	406	eMol27203000	−7.4	−7.7
355	CHEMBL2403015	−7.8	−7.8	407	eMol43745056	−7.3	−7.7
356	CHEMBL3819445	−8.1	−7.8	408	eMol25810081	−7.1	−7.7
357	eMol16374637	−7.5	−7.8	409	eMol3060078	−7.6	−7.7
358	eMol1751766	−7.5	−7.8	410	eMol45701494	−7.5	−7.7
359	eMol3427847	−7.5	−7.8	411	eMol16683412	−7.5	−7.7
329	eMol50301465	−7.2	−7.8	381	eMol26310961	−7.7	−7.7

Table S2. *Continued.*

No.	Compound Name/Code	Docking score (kcal/mol)		No.	Compound Name/Code	Docking score (kcal/mol)	
		Conventional	Intermediate			Conventional	Intermediate
412	eMol26405201	−7.5	−7.7	464	eMol43730464	−7.2	−7.6
413	eMol27202262	−7.6	−7.7	465	eMol4551561	−7.5	−7.6
414	eMol43731776	−7.1	−7.7	466	eMol4577787	−7.6	−7.6
415	eMol565754	−7.5	−7.7	467	eMol588256	−7.5	−7.6
416	eMol233088901	−7.5	−7.6	468	eMol9919488	−7.4	−7.6
417	eMol4574833	−7.6	−7.6	469	CHEMBL117642	−7.4	−7.6
418	eMol1675430	−7.6	−7.6	470	CHEMBL511010	−7.4	−7.6
419	eMol1764190	−7.3	−7.6	471	eMol25896479	−7.5	−7.6
420	eMol25784115	−7.6	−7.6	472	eMol43906063	−7.9	−7.6
421	eMol32369766	−7.6	−7.6	473	eMol46400681	−7.5	−7.6
422	CHEMBL3818012	−7.5	−7.6	474	eMol8238281	−7.3	−7.6
423	CHEMBL520033	−7.5	−7.6	475	CHEMBL1994876	−7.4	−7.5
424	eMol27202960	−7.5	−7.6	476	CHEMBL250917	−7.5	−7.5
425	eMol4075283	−7.3	−7.6	477	CHEMBL3817957	−7.5	−7.5
426	eMol4587779	−7.5	−7.6	478	eMol1679768	−7.5	−7.5
427	eMol956409	−7.6	−7.6	479	eMol27202984	−7.3	−7.5
428	eMol24116475	−7.5	−7.6	480	eMol4480890	−7.5	−7.5
429	eMol26327269	−7.6	−7.6	481	eMol45708568	−7.2	−7.5
430	eMol32812942	−7.4	−7.6	482	eMol1025907	−7.4	−7.5
431	eMol6753441	−7.5	−7.6	483	eMol18940061	−7.5	−7.5
432	eMol944638	−7.6	−7.6	484	eMol206875270	−7.4	−7.5
433	eMol1429358	−7.5	−7.6	485	eMol299962831	−7.4	−7.5
434	eMol179177638	−7.4	−7.6	486	CHEMBL1584455	−7.5	−7.5
435	eMol25909178	−7.7	−7.6	487	CHEMBL3819326	−7.5	−7.5
436	eMol46480052	−7.6	−7.6	488	eMol23250559	−7.5	−7.5
437	CHEMBL3818704	−7.4	−7.6	489	eMol25810127	−7.5	−7.5
438	CHEMBL397391	−7.3	−7.6	490	eMol4386746	−7.5	−7.5
439	eMol32246523	−7.2	−7.6	491	eMol45944068	−7.5	−7.5
440	eMol561655	−7.3	−7.6	492	CHEMBL1499977	−7.4	−7.5
441	eMol588102	−7.6	−7.6	493	CHEMBL3805023	−7.4	−7.5
442	eMol7635480	−7.6	−7.6	494	CHEMBL462179	−7.9	−7.5
443	CHEMBL114364	−7.5	−7.6	495	eMol1040103	−7.5	−7.5
444	eMol18958642	−7.6	−7.6	496	eMol181128729	−7.4	−7.5
445	eMol24058094	−7.4	−7.6	497	eMol19266607	−7.5	−7.5
446	eMol46509913	−7.1	−7.6	498	eMol25937897	−7.4	−7.5
447	eMol46509917	−7.6	−7.6	499	eMol26255667	−7.5	−7.5
448	eMol5779489	−7.6	−7.6	500	eMol27202250	−7.4	−7.5
449	eMol587808	−7.4	−7.6	501	eMol27202902	−7.4	−7.5
450	eMol588228	−7.2	−7.6	502	eMol2993022	−7.4	−7.5
451	CHEMBL117438	−7.2	−7.6	503	eMol300014864	−7.5	−7.5
452	CHEMBL1423402	−7.1	−7.6	504	eMol46479990	−7.5	−7.5
453	eMol206987969	−7.5	−7.6	505	eMol565838	−7.3	−7.5
454	eMol2130046	−7.2	−7.6	506	CHEMBL116817	−7.2	−7.5
455	eMol36904288	−7.1	−7.6	507	eMol206908008	−7.5	−7.5
456	eMol4448933	−7.5	−7.6	508	eMol206985560	−7.5	−7.5
457	eMol5444969	−7.2	−7.6	509	eMol24520756	−7.4	−7.5
458	eMol935130	−7.5	−7.6	510	eMol25902291	−7.2	−7.5
459	CHEMBL1214868	−7.6	−7.6	511	eMol276247970	−7.3	−7.5
460	CHEMBL3818515	−7.5	−7.6	512	eMol300990951	−7.3	−7.5
461	eMol20377433	−7.6	−7.6	513	eMol3317346	−7.2	−7.5
462	eMol26683947	−7.6	−7.6	514	eMol4485131	−7.3	−7.5
463	eMol28321866	−7.5	−7.6	515	eMol4725672	−7.4	−7.5

Table S2. *Continued.*

No.	Compound Name/Code	Docking score (kcal/mol)		No.	Compound Name/Code	Docking score (kcal/mol)	
		Conventional	Intermediate			Conventional	Intermediate
516	CHEMBL1360793	−7.4	−7.5	568	eMol4706083	−7.2	−7.4
517	CHEMBL602627	−7.5	−7.5	569	eMol16352353	−7.4	−7.4
518	eMol20356176	−7.4	−7.5	570	eMol24519100	−7.0	−7.4
519	eMol25816251	−7.5	−7.5	571	eMol26327656	−7.4	−7.4
520	eMol27091590	−7.3	−7.5	572	eMol27338418	−7.4	−7.4
521	eMol4521223	−7.4	−7.5	573	eMol46210806	−7.4	−7.4
522	eMol4567258	−7.5	−7.5	574	eMol48597407	−7.1	−7.4
523	eMol45935466	−7.5	−7.5	575	CHEMBL3818933	−7.3	−7.4
524	eMol684240	−7.3	−7.5	576	eMol1324430	−7.3	−7.4
525	CHEMBL1988177	−7.4	−7.5	577	eMol18551352	−7.2	−7.4
526	eMol25903079	−7.4	−7.5	578	eMol2166708	−7.1	−7.4
527	eMol25965334	−7.4	−7.5	579	eMol23246986	−7.2	−7.4
528	eMol26250892	−7.3	−7.5	580	eMol2385157	−7.3	−7.4
529	eMol45928112	−7.4	−7.5	581	eMol25902329	−7.4	−7.4
530	eMol46528447	−7.5	−7.5	582	eMol26219237	−7.2	−7.4
531	CHEMBL116408	−7.3	−7.5	583	eMol26246365	−7.4	−7.4
532	eMol20354197	−7.4	−7.5	584	eMol4572232	−7.1	−7.4
533	eMol2880336	−7.3	−7.5	585	eMol935636	−7.2	−7.4
534	eMol49384601	−7.5	−7.5	586	CHEMBL3805378	−7.4	−7.4
535	eMol75932399	−7.4	−7.5	587	CHEMBL3819459	−7.0	−7.4
536	eMol20337444	−7.5	−7.5	588	CHEMBL3819532	−7.4	−7.4
537	eMol24419686	−7.2	−7.5	589	eMol25903125	−7.4	−7.4
538	eMol27202290	−7.5	−7.5	590	eMol32812998	−7.1	−7.4
539	eMol3535618	−7.5	−7.5	591	eMol32813226	−7.2	−7.4
540	eMol579714	−7.3	−7.5	592	eMol32813234	−7.2	−7.4
541	eMol24526444	−7.3	−7.5	593	eMol46479912	−7.4	−7.4
542	eMol27187406	−7.5	−7.5	594	eMol4934666	−7.4	−7.4
543	eMol27202402	−7.4	−7.5	595	eMol936420	−7.1	−7.4
544	eMol31760166	−7.2	−7.5	596	CHEMBL1402619	−7.4	−7.4
545	eMol46480026	−7.4	−7.5	597	CHEMBL3818910	−7.3	−7.4
546	eMol46480194	−7.7	−7.5	598	eMol1040791	−7.3	−7.4
547	eMol2408165	−7.1	−7.4	599	eMol16351683	−7.4	−7.4
548	eMol33346433	−7.1	−7.4	600	eMol206921779	−7.2	−7.4
549	eMol565478	−7.4	−7.4	601	eMol25810171	−7.4	−7.4
550	CHEMBL455639	−7.3	−7.4	602	eMol26324339	−7.3	−7.4
551	eMol1149869	−7.0	−7.4	603	eMol27202936	−7.4	−7.4
552	eMol18550714	−7.2	−7.4	604	eMol4739467	−7.4	−7.4
553	eMol23247039	−7.4	−7.4	605	eMol72578631	−7.0	−7.4
554	eMol24524408	−7.4	−7.4	606	eMol1678605	−7.3	−7.4
555	eMol4708971	−7.3	−7.4	607	eMol19076750	−7.3	−7.4
556	eMol588158	−7.3	−7.4	608	eMol24520553	−7.1	−7.4
557	eMol936632	−7.2	−7.4	609	eMol24538139	−7.3	−7.4
558	eMol956403	−7.4	−7.4	610	eMol25829045	−7.2	−7.4
559	CHEMBL3818229	−7.2	−7.4	611	eMol26936203	−7.4	−7.4
560	eMol16056893	−7.2	−7.4	612	eMol27202398	−7.2	−7.4
561	eMol20378545	−7.4	−7.4	613	eMol276247952	−7.3	−7.4
562	eMol24427986	−7.0	−7.4	614	eMol300978663	−7.3	−7.4
563	eMol25918241	−7.3	−7.4	615	eMol4490859	−7.2	−7.4
564	eMol26316348	−7.4	−7.4	616	eMol46445765	−7.2	−7.4
565	eMol4565043	−7.0	−7.4	617	eMol206850549	−7.3	−7.4
566	eMol46479157	−7.4	−7.4	618	eMol207259512	−7.3	−7.4
567	eMol46479980	−7.4	−7.4	619	eMol299991997	−7.3	−7.4
537	eMol24419686	−7.2	−7.5	589	eMol25903125	−7.4	−7.4

Table S2. *Continued.*

No.	Compound Name/Code	Docking score (kcal/mol)		No.	Compound Name/Code	Docking score (kcal/mol)	
		Conventional	Intermediate			Conventional	Intermediate
620	eMol4732807	−7.3	−7.4	672	eMol43731198	−7.1	−7.3
621	eMol956477	−7.3	−7.4	673	eMol566406	−7.2	−7.3
622	CHEMBL3818171	−7.1	−7.4	674	eMol588242	−7.7	−7.3
623	CHEMBL579620	−7.2	−7.4	675	CHEMBL3805665	−7.1	−7.3
624	eMol1683032	−7.3	−7.4	676	CHEMBL3819311	−7.2	−7.3
625	eMol20337442	−7.1	−7.4	677	CHEMBL38330	−7.1	−7.3
626	eMol2141753	−7.2	−7.4	678	eMol11071566	−7.3	−7.3
627	eMol25968613	−7.2	−7.4	679	eMol206964256	−7.3	−7.3
628	eMol26245604	−7.3	−7.4	680	eMol23206884	−7.3	−7.3
629	eMol27202970	−7.3	−7.4	681	eMol24520802	−7.3	−7.3
630	eMol4606566	−7.2	−7.4	682	eMol25771182	−7.3	−7.3
631	eMol4715267	−7.3	−7.4	683	eMol25907499	−7.3	−7.3
632	eMol4728946	−7.3	−7.4	684	eMol25913211	−7.3	−7.3
633	CHEMBL114005	−7.1	−7.3	685	eMol26262902	−7.3	−7.3
634	eMol18961180	−7.2	−7.3	686	eMol316831112	−7.2	−7.3
635	eMol19076404	−7.4	−7.3	687	eMol4433627	−7.2	−7.3
636	eMol206907968	−7.3	−7.3	688	eMol4580053	−7.5	−7.3
637	eMol24520050	−7.3	−7.3	689	eMol24532024	−7.3	−7.3
638	eMol316344188	−7.3	−7.3	690	eMol25958221	−7.2	−7.3
639	eMol44673597	−7.3	−7.3	691	eMol26264151	−7.1	−7.3
640	eMol4698359	−7.4	−7.3	692	eMol26300005	−7.2	−7.3
641	eMol4718679	−7.2	−7.3	693	eMol26326968	−7.3	−7.3
642	eMol17203597	−7.0	−7.3	694	eMol26936207	−7.2	−7.3
643	eMol180102976	−7.3	−7.3	695	eMol27187414	−7.1	−7.3
644	eMol25797977	−7.3	−7.3	696	eMol27202912	−7.3	−7.3
645	eMol29649914	−7.3	−7.3	697	eMol579510	−7.1	−7.3
646	eMol4477952	−7.3	−7.3	698	eMol935864	−7.6	−7.3
647	eMol45622753	−7.3	−7.3	699	CHEMBL113923	−7.1	−7.3
648	CHEMBL115809	−7.3	−7.3	700	CHEMBL47157	−7.2	−7.3
649	CHEMBL116634	−7.3	−7.3	701	eMol179164953	−7.2	−7.3
650	eMol17192354	−7.2	−7.3	702	eMol20380394	−7.1	−7.3
651	eMol179164956	−7.2	−7.3	703	eMol2330950	−7.3	−7.3
652	eMol23242679	−7.3	−7.3	704	eMol25902283	−7.3	−7.3
653	eMol25901925	−7.3	−7.3	705	eMol26285150	−7.3	−7.3
654	eMol25936279	−7.3	−7.3	706	eMol27187400	−7.1	−7.3
655	eMol26230266	−7.3	−7.3	707	eMol46400679	−7.3	−7.3
656	eMol26315986	−7.3	−7.3	708	eMol4713307	−7.2	−7.3
657	eMol27252901	−7.3	−7.3	709	CHEMBL4099863	−7.2	−7.3
658	eMol4713633	−7.3	−7.3	710	eMol24521048	−7.3	−7.3
659	CHEMBL3819196	−7.1	−7.3	711	eMol25966304	−7.3	−7.3
660	eMol16797514	−8.1	−7.3	712	eMol27202388	−7.3	−7.3
661	eMol19076688	−7.3	−7.3	713	eMol27202918	−7.2	−7.3
662	eMol20375154	−7.3	−7.3	714	eMol46400677	−7.2	−7.3
663	eMol26300049	−7.3	−7.3	715	eMol4709495	−7.2	−7.3
664	eMol46370139	−7.3	−7.3	716	CHEMBL1302012	−7.1	−7.3
665	eMol51435800	−7.5	−7.3	717	CHEMBL2297150	−7.0	−7.3
666	CHEMBL1383006	−7.1	−7.3	718	CHEMBL326628	−7.2	−7.3
667	eMol17202599	−7.1	−7.3	719	CHEMBL3817866	−7.2	−7.3
668	eMol180102764	−7.2	−7.3	720	CHEMBL3818856	−7.1	−7.3
669	eMol23191317	−7.3	−7.3	721	CHEMBL577676	−7.2	−7.3
670	eMol2409922	−7.2	−7.3	722	CHEMBL585981	−7.2	−7.3
671	eMol26251302	−7.1	−7.3	723	eMol1120546	−7.2	−7.3
641	eMol4718679	−7.2	−7.3	693	eMol26326968	−7.3	−7.3

Table S2. *Continued.*

No.	Compound Name/Code	Docking score (kcal/mol)		No.	Compound Name/Code	Docking score (kcal/mol)	
		**Conventional**	**Intermediate**			**Conventional**	**Intermediate**
724	eMol18866942	−7.1	−7.3	776	eMol45133343	−7.2	−7.2
725	eMol24113359	−7.2	−7.3	777	eMol46491554	−7.2	−7.2
726	eMol25825125	−7.2	−7.3	778	eMol588078	−7.1	−7.2
727	eMol26250339	−7.1	−7.3	779	eMol7265152	−7.6	−7.2
728	eMol26285928	−7.2	−7.3	780	eMol22370884	−7.2	−7.2
729	eMol26314361	−7.2	−7.3	781	eMol24521662	−7.2	−7.2
730	eMol26569999	−7.2	−7.3	782	eMol24526765	−7.1	−7.2
731	eMol29987858	−7.0	−7.3	783	eMol2517109	−7.1	−7.2
732	eMol4408548	−7.2	−7.3	784	eMol25963663	−7.2	−7.2
733	eMol44127206	−7.4	−7.3	785	eMol26328784	−7.2	−7.2
734	eMol46479958	−7.2	−7.3	786	eMol27202280	−7.1	−7.2
735	eMol4726520	−7.2	−7.3	787	eMol27202496	−7.1	−7.2
736	CHEMBL296691	−7.2	−7.2	788	eMol27202506	−7.2	−7.2
737	eMol19076602	−7.2	−7.2	789	eMol27202540	−7.2	−7.2
738	eMol24520240	−7.2	−7.2	790	eMol30744925	−7.3	−7.2
739	eMol24521330	−7.2	−7.2	791	eMol4146706	−7.1	−7.2
740	eMol25902049	−7.1	−7.2	792	eMol43744846	−7.1	−7.2
741	eMol26285666	−7.2	−7.2	793	eMol46400687	−7.2	−7.2
742	eMol26314117	−7.1	−7.2	794	eMol46479996	−7.2	−7.2
743	eMol27202754	−7.2	−7.2	795	eMol5672543	−7.1	−7.2
744	eMol2768990	−7.2	−7.2	796	CHEMBL3805042	−7.2	−7.2
745	eMol29665906	−7.1	−7.2	797	CHEMBL3806178	−7.2	−7.2
746	eMol43731202	−7.7	−7.2	798	CHEMBL3818209	−7.1	−7.2
747	eMol46370137	−7.2	−7.2	799	eMol18550720	−7.2	−7.2
748	eMol566256	−7.5	−7.2	800	eMol206987841	−7.0	−7.2
749	eMol956435	−7.3	−7.2	801	eMol23201309	−7.2	−7.2
750	CHEMBL1559857	−7.1	−7.2	802	eMol25819233	−7.1	−7.2
751	CHEMBL3806097	−7.2	−7.2	803	eMol26314895	−7.1	−7.2
752	CHEMBL3806218	−7.1	−7.2	804	eMol26315041	−7.0	−7.2
753	eMol16351673	−7.1	−7.2	805	eMol27202282	−7.1	−7.2
754	eMol18516265	−7.2	−7.2	806	eMol27202756	−7.2	−7.2
755	eMol18550716	−7.1	−7.2	807	CHEMBL1562035	−7.1	−7.2
756	eMol23241510	−7.2	−7.2	808	eMol25912447	−7.1	−7.2
757	eMol25770408	−7.2	−7.2	809	eMol26277244	−7.2	−7.2
758	eMol26289047	−7.2	−7.2	810	eMol26292416	−7.2	−7.2
759	eMol27045407	−7.2	−7.2	811	eMol26316052	−7.2	−7.2
760	eMol27202350	−7.2	−7.2	812	eMol27202358	−7.2	−7.2
761	eMol27202914	−7.2	−7.2	813	CHEMBL34185	−7.1	−7.2
762	eMol43746869	−7.2	−7.2	814	CHEMBL3818747	−7.1	−7.2
763	eMol5444645	−7.1	−7.2	815	eMol2208925	−7.5	−7.2
764	eMol5847921	−7.2	−7.2	816	eMol2308510	−7.0	−7.2
765	CHEMBL1552848	−7.1	−7.2	817	eMol23152351	−7.0	−7.2
766	CHEMBL247353	−7.1	−7.2	818	eMol23170772	−7.2	−7.2
767	CHEMBL4079984	−7.2	−7.2	819	eMol25810099	−7.2	−7.2
768	eMol18860491	−7.5	−7.2	820	eMol26289870	−7.2	−7.2
769	eMol19076828	−7.0	−7.2	821	eMol26310907	−7.2	−7.2
770	eMol2115166	−7.1	−7.2	822	eMol27202410	−7.2	−7.2
771	eMol26293580	−7.1	−7.2	823	eMol27202762	−7.2	−7.2
772	eMol27045405	−7.2	−7.2	824	eMol206964558	−7.1	−7.2
773	eMol27202232	−7.1	−7.2	825	eMol23185851	−7.1	−7.2
774	eMol27202920	−7.2	−7.2	826	eMol23241342	−7.2	−7.2
775	eMol30322739	−7.2	−7.2	827	eMol24518463	−7.2	−7.2

Table S2. *Continued.*

No.	Compound Name/Code	Docking score (kcal/mol)		No.	Compound Name/Code	Docking score (kcal/mol)	
		Conventional	Intermediate			Conventional	Intermediate
828	eMol26330383	−7.2	−7.2	880	eMol25816255	−7.0	−7.1
829	eMol26936201	−7.1	−7.2	881	eMol26276632	−7.1	−7.1
830	eMol27045521	−7.2	−7.2	882	CHEMBL1551922	−7.1	−7.1
831	eMol36016142	−7.1	−7.2	883	CHEMBL3818499	−7.1	−7.1
832	eMol46545587	−7.1	−7.2	884	CHEMBL3818977	−7.1	−7.1
833	eMol24428006	−7.1	−7.2	885	eMol206990555	−7.1	−7.1
834	eMol25770418	−7.1	−7.2	886	eMol24524699	−7.1	−7.1
835	eMol26310653	−7.0	−7.2	887	eMol25771206	−7.1	−7.1
836	eMol26315688	−7.1	−7.2	888	eMol26286396	−7.1	−7.1
837	eMol560303	−7.1	−7.2	889	eMol26311934	−7.4	−7.1
838	CHEMBL3805407	−7.1	−7.2	890	eMol26312838	−7.1	−7.1
839	eMol24519079	−7.2	−7.2	891	eMol26314721	−7.3	−7.1
840	eMol24521248	−7.1	−7.2	892	eMol27202336	−7.1	−7.1
841	eMol26189151	−7.1	−7.2	893	eMol27202362	−7.1	−7.1
842	eMol26292060	−7.1	−7.2	894	eMol30104228	−7.1	−7.1
843	eMol26310839	−7.1	−7.2	895	eMol43731200	−8.0	−7.1
844	eMol26314359	−7.1	−7.2	896	CHEMBL3804857	−7.1	−7.1
845	eMol26936209	−7.1	−7.2	897	eMol1165997	−7.1	−7.1
846	eMol45932769	−7.1	−7.2	898	eMol16352193	−7.0	−7.1
847	eMol46534208	−7.1	−7.2	899	eMol19076826	−7.1	−7.1
848	eMol6263477	−7.1	−7.2	900	eMol206920578	−7.1	−7.1
849	eMol106491702	−7.1	−7.1	901	eMol2231344	−7.2	−7.1
850	eMol16351675	−7.1	−7.1	902	eMol25924517	−7.1	−7.1
851	eMol19076732	−7.0	−7.1	903	eMol25966294	−7.1	−7.1
852	eMol206980481	−7.1	−7.1	904	eMol316862506	−7.1	−7.1
853	eMol23242609	−7.1	−7.1	905	CHEMBL299107	−7.1	−7.1
854	eMol26313449	−7.1	−7.1	906	CHEMBL3804866	−7.1	−7.1
855	eMol26319543	−7.1	−7.1	907	CHEMBL3818626	−7.0	−7.1
856	CHEMBL1414265	−7.1	−7.1	908	eMol15983174	−7.0	−7.1
857	eMol1156520	−7.2	−7.1	909	eMol1748795	−7.7	−7.1
858	eMol20344945	−7.1	−7.1	910	eMol24520023	−7.1	−7.1
859	eMol206965980	−7.1	−7.1	911	eMol24531798	−7.1	−7.1
860	eMol26270766	−7.1	−7.1	912	eMol25913057	−7.1	−7.1
861	eMol26276502	−7.1	−7.1	913	eMol26288955	−7.1	−7.1
862	eMol4491662	−7.1	−7.1	914	eMol26326313	−7.1	−7.1
863	CHEMBL1394809	−7.0	−7.1	915	eMol31278496	−7.1	−7.1
864	eMol16351677	−7.1	−7.1	916	eMol46400683	−7.0	−7.1
865	eMol18542840	−7.1	−7.1	917	eMol566142	−7.4	−7.1
866	eMol20380392	−7.1	−7.1	918	eMol579926	−7.1	−7.1
867	eMol206843436	−7.0	−7.1	919	CHEMBL1439596	−7.1	−7.1
868	eMol24428326	−7.4	−7.1	920	CHEMBL550397	−7.1	−7.1
869	eMol25790309	−7.1	−7.1	921	eMol20378615	−7.1	−7.1
870	eMol26331720	−7.1	−7.1	922	eMol24430231	−7.1	−7.1
871	eMol26350850	−7.1	−7.1	923	eMol24531722	−7.1	−7.1
872	eMol26570001	−7.1	−7.1	924	eMol26314551	−7.0	−7.1
873	eMol27187440	−7.1	−7.1	925	eMol4709315	−7.0	−7.1
874	eMol45935468	−7.1	−7.1	926	eMol6157528	−7.0	−7.1
875	eMol588368	−7.1	−7.1	927	CHEMBL1215796	−7.1	−7.1
876	CHEMBL3818347	−7.0	−7.1	928	eMol19076734	−7.0	−7.1
877	eMol1035551	−7.1	−7.1	929	eMol20310561	−7.1	−7.1
878	eMol18550722	−7.1	−7.1	930	eMol206849669	−7.1	−7.1
879	eMol19076370	−7.0	−7.1	931	eMol25945602	−7.1	−7.1
849	eMol106491702	−7.1	−7.1	901	eMol2231344	−7.2	−7.1

Table S2. *Continued.*

No.	Compound Name/Code	Docking score (kcal/mol)		No.	Compound Name/Code	Docking score (kcal/mol)	
		Conventional	Intermediate			Conventional	Intermediate
932	eMol299994300	−7.1	−7.1	967	eMol43731774	−7.2	−6.9
933	eMol300089978	−7.1	−7.1	968	eMol3018072	−7.4	−6.9
934	eMol956263	−7.1	−7.1	969	eMol316351191	−7.5	−6.9
935	CHEMBL1373990	−7.0	−7.1	970	eMol206964210	−7.0	−6.8
936	eMol16379841	−7.0	−7.1	971	eMol27115486	−7.3	−6.8
937	eMol1752196	−7.0	−7.1	972	eMol23244410	−7.1	−6.8
938	eMol25895011	−7.0	−7.1	973	eMol33339294	−7.1	−6.7
939	eMol25913175	−7.1	−7.1	974	eMol2992508	−7.6	−6.7
940	eMol26524217	−7.0	−7.1	975	eMol4732869	−7.1	−6.7
941	eMol3033983	−7.1	−7.1	976	CHEMBL395117	−7.1	−6.6
942	eMol207355370	−7.3	−7.0	977	eMol4738130	−7.2	−6.6
943	eMol25895009	−7.0	−7.0	978	eMol25895023	−7.3	−6.5
944	eMol679754	−7.1	−7.0	979	eMol27380659	−7.8	−6.5
945	CHEMBL3818366	−7.0	−7.0	980	eMol1149947	−7.3	−6.5
946	eMol206965358	−7.0	−7.0	981	eMol27202380	−7.1	−6.5
947	eMol25901881	−7.0	−7.0	982	eMol33346435	−7.2	−6.5
948	eMol26299531	−7.0	−7.0	983	CHEMBL82690	−7.4	−6.3
949	eMol26324447	−7.0	−7.0	984	eMol11690694	−7.5	−6.3
950	eMol27202422	−7.1	−7.0	985	eMol1764376	−7.6	−6.2
951	eMol3775722	−7.0	−7.0	986	eMol2992482	−8.1	−6.2
952	CHEMBL1900041	−7.0	−7.0	987	eMol2194316	−7.1	−6.1
953	CHEMBL3804880	−7.0	−7.0	988	eMol16375743	−7.1	−6.1
954	eMol181109768	−7.2	−7.0	989	eMol36016132	−7.1	−6.0
955	eMol20356657	−7.0	−7.0	990	eMol16351883	−7.1	−5.8
956	CHEMBL2297667	−7.0	−7.0	991	eMol179165005	−7.3	−5.8
957	eMol4388132	−7.4	−7.0	992	eMol587916	−7.1	−5.7
958	eMol27202320	−7.0	−7.0	993	eMol43745829	−7.3	−5.7
959	eMol25903659	−7.1	−7.0	994	eMol2992488	−7.6	−5.6
960	eMol44548966	−7.2	−7.0	995	eMol24493983	−7.4	−5.5
961	eMol565918	−7.2	−6.9	996	eMol24427880	−7.4	−5.4
962	eMol206909136	−7.1	−6.9	997	eMol5444883	−7.1	−5.3
963	eMol581304	−7.3	−6.9	998	eMol31358919	−7.3	−5.3
964	eMol46411087	−7.4	−6.9	999	eMol3181655	−7.1	−5.1
965	CHEMBL1591720	−7.1	−6.9	1000	CHEMBL1214720	−8.8	−5.1
966	eMol27202778	−7.1	−6.9				

[a] Data ranked based on the intermediate docking scores.

Table S3. Estimated conventional, intermediate, and Expensive docking scores for QNZ and the top 250 potent allosteric inhibitors against mutated PBP2a allosteric site in acidic medium.[a]

No.	Compound Name/Code	Docking Score (kcal/mol)			No.	Compound Name/Code	Docking Score (kcal/mol)		
		Conventional	Intermediate	Expensive			Conventional	Intermediate	Expensive
	QNZ	−6.9	−8.0	−8.3	63	eMol30323633	−9.0	−9.1	−8.2
1	eMol26313223	−8.3	−8.4	−10.0	64	CHEMBL1214938	−8.7	−9.0	−8.2
2	eMol26437582	−8.2	−8.9	−9.9	65	eMol20375178	−8.7	−9.0	−8.2
3	eMol26314565	−8.2	−8.2	−9.6	66	eMol30323569	−8.6	−9.0	−8.2
4	eMol26313117	−8.9	−9.4	−9.5	67	eMol30341049	−8.8	−8.9	−8.2
5	eMol26293960	−8.1	−9.2	−9.4	68	eMol43906045	−8.8	−8.9	−8.2
6	eMol3021959	−7.1	−8.9	−9.4	69	CHEMBL3818584	−8.3	−8.9	−8.2
7	eMol29597031	−9.2	−9.3	−9.3	70	eMol20378769	−8.7	−8.9	−8.2
8	eMol26269394	−8.5	−8.7	−9.1	71	eMol20342262	−7.9	−8.8	−8.2
9	eMol27202760	−8.0	−8.1	−9.1	72	eMol19076712	−8.9	−8.9	−8.2
10	eMol26242018	−8.0	−8.8	−9.0	73	eMol18542846	−8.8	−8.8	−8.2
11	eMol29633913	−7.8	−8.6	−8.9	74	CHEMBL1214941	−8.6	−8.1	−8.1
12	CHEMBL1215080	−8.4	−8.4	−8.9	75	eMol1166007	−8.7	−8.8	−8.1
13	eMol27252412	−8.1	−8.2	−8.7	76	CHEMBL3805841	−7.7	−8.0	−8.1
14	eMol30017880	−7.4	−8.3	−8.7	77	eMol20337486	−8.8	−8.8	−8.1
15	eMol26319231	−8.2	−8.6	−8.7	78	eMol23190351	−7.9	−8.7	−8.1
16	eMol26264570	−8.1	−8.4	−8.7	79	eMol19076754	−8.6	−8.8	−8.1
17	eMol300094331	−8.2	−8.3	−8.7	80	eMol31781482	−8.7	−8.6	−8.1
18	eMol29565259	−7.8	−8.2	−8.6	81	CHEMBL1215649	−8.5	−8.6	−8.1
19	eMol27406062	−7.8	−8.3	−8.6	82	eMol26196316	−8.5	−8.6	−8.1
20	eMol299980544	−8.2	−8.2	−8.6	83	eMol33366267	−7.9	−8.6	−8.1
21	eMol27202252	−8.0	−8.1	−8.6	84	eMol75958504	−7.9	−8.6	−8.1
22	eMol29634797	−7.6	−8.1	−8.6	85	CHEMBL1215648	−8.6	−8.7	−8.1
23	eMol27202248	−8.4	−8.3	−8.6	86	eMol20379909	−8.8	−8.8	−8.1
24	CHEMBL1215082	−7.4	−8.0	−8.5	87	eMol26196326	−8.6	−8.7	−8.1
25	eMol26242042	−7.6	−8.5	−8.5	88	eMol45585842	−8.6	−8.7	−8.1
26	eMol300154219	−7.3	−8.3	−8.5	89	eMol23241028	−8.6	−8.7	−8.1
27	eMol26242026	−7.8	−8.2	−8.4	90	eMol45622743	−7.8	−8.6	−8.1
28	eMol26330545	−8.2	−8.2	−8.4	91	CHEMBL1215650	−8.5	−8.7	−8.1
29	eMol27091674	−8.0	−8.1	−8.4	92	CHEMBL1579061	−8.5	−8.7	−8.1
30	CHEMBL1215004	−7.7	−8.2	−8.4	93	eMol30646140	−7.7	−8.6	−8.1
31	eMol27091498	−8.0	−8.1	−8.4	94	eMol72993476	−7.7	−8.6	−8.1
32	eMol29587306	−7.7	−8.2	−8.4	95	eMol22447774	−8.5	−8.7	−8.1
33	eMol26314779	−8.1	−8.2	−8.3	96	eMol30248263	−8.0	−8.4	−8.0
34	eMol27202948	−7.4	−8.0	−8.3	97	eMol19076742	−8.6	−8.7	−8.0
35	eMol301527162	−8.0	−8.1	−8.3	98	eMol23157132	−8.4	−8.6	−8.0
36	eMol19266609	−7.9	−9.5	−8.2	99	eMol26242050	−8.1	−8.6	−8.0
37	eMol24519694	−9.0	−9.5	−8.2	100	CHEMBL1214713	−8.2	−8.6	−8.0
38	CHEMBL1443074	−8.0	−8.1	−8.2	101	CHEMBL1384683	−8.2	−8.6	−8.0
39	eMol18940433	−8.0	−8.1	−8.2	102	CHEMBL1558437	−8.2	−8.6	−8.0
40	CHEMBL1214717	−9.4	−9.4	−8.2	103	CHEMBL3818129	−8.5	−8.6	−8.0
41	eMol29587310	−9.4	−9.4	−8.2	104	eMol26270654	−8.2	−8.6	−8.0
42	eMol45585846	−9.4	−9.4	−8.2	105	eMol300994534	−8.4	−8.6	−8.0
43	eMol24389393	−7.9	−9.4	−8.2	106	eMol48938145	−8.4	−8.6	−8.0
44	eMol23239806	−9.3	−9.4	−8.2	107	eMol30671942	−7.9	−8.5	−8.0
45	CHEMBL1214718	−9.2	−9.3	−8.2	108	eMol23246696	−8.5	−8.6	−8.0
46	eMol45585848	−9.2	−9.3	−8.2	109	eMol26242044	−8.3	−8.4	−8.0
47	eMol31786896	−8.4	−9.3	−8.2	110	eMol36912832	−7.9	−8.4	−8.0
48	eMol2403964	−8.7	−9.3	−8.2	111	eMol26242034	−8.2	−8.5	−7.9
49	eMol23186178	−9.1	−9.3	−8.2	112	CHEMBL3805924	−8.3	−8.5	−7.9
50	eMol31297102	−7.9	−9.2	−8.2	113	eMol23175530	−8.3	−8.5	−7.9
51	eMol19076714	−8.4	−9.2	−8.2	114	eMol24519844	−8.4	−8.5	−7.9
52	eMol19076718	−8.9	−9.1	−8.2	115	CHEMBL3582080	−7.9	−8.1	−7.9
53	eMol18542844	−9.1	−9.1	−8.2	116	eMol1344114	−9.0	−9.1	−7.9
54	CHEMBL1214794	−8.9	−9.0	−8.2	117	eMol26242032	−7.9	−8.3	−7.9
55	eMol25810077	−7.9	−8.4	−8.2	118	eMol43906059	−8.4	−8.4	−7.9
56	eMol26196290	−8.9	−9.0	−8.2	119	eMol23243407	−8.2	−8.4	−7.9
57	eMol45585824	−8.9	−9.0	−8.2	120	eMol43906055	−8.0	−8.4	−7.9
58	CHEMBL3819187	−7.8	−9.1	−8.2	121	eMol579568	−8.4	−8.4	−7.8
59	CHEMBL3818732	−8.0	−9.0	−8.2	122	eMol23239141	−8.3	−8.4	−7.8
60	eMol26262168	−7.0	−8.0	−8.2	123	eMol588192	−8.2	−8.4	−7.8
61	eMol29638777	−7.8	−8.1	−8.2	124	CHEMBL4103504	−8.3	−8.5	−7.8
62	eMol46480173	−8.2	−8.3	−8.2	125	eMol23181799	−8.3	−8.4	−7.8

Table S3. *Continued***.**

No.	Compound Name/Code	Docking Score (kcal/mol)			No.	Compound Name/Code	Docking Score (kcal/mol)		
		Conventional	Intermediate	Expensive			Conventional	Intermediate	Expensive
126	eMol587826	−8.2	−8.4	−7.8	189	eMol4566508	−7.3	−8.2	−7.6
127	eMol679826	−8.3	−8.4	−7.8	190	eMol20368046	−8.1	−8.2	−7.6
128	eMol27202910	−8.3	−8.4	−7.8	191	eMol23147497	−8.1	−8.2	−7.6
129	CHEMBL3818572	−8.0	−8.3	−7.8	192	eMol679824	−8.0	−8.2	−7.6
130	eMol18962382	−8.4	−8.4	−7.8	193	eMol24428240	−7.2	−8.5	−7.6
131	eMol27202240	−8.4	−8.4	−7.8	194	eMol37002179	−7.4	−8.1	−7.6
132	eMol679820	−7.2	−8.4	−7.8	195	eMol45585838	−7.8	−8.2	−7.6
133	eMol31532412	−8.3	−8.4	−7.8	196	CHEMBL1601398	−8.0	−8.1	−7.6
134	CHEMBL1215003	−8.2	−8.3	−7.8	197	eMol2507718	−8.0	−8.1	−7.6
135	eMol25810075	−8.3	−8.4	−7.8	198	eMol26242024	−7.7	−8.2	−7.6
136	eMol2992792	−8.1	−8.3	−7.8	199	eMol4558540	−8.0	−8.2	−7.6
137	eMol43731476	−7.6	−8.3	−7.8	200	eMol256911679	−8.0	−8.1	−7.6
138	eMol43906053	−8.2	−8.3	−7.8	201	eMol4562556	−8.1	−8.1	−7.6
139	CHEMBL395193	−8.3	−8.3	−7.8	202	eMol45935562	−7.5	−8.1	−7.6
140	eMol193446288	−8.3	−8.3	−7.8	203	eMol565850	−7.0	−8.1	−7.6
141	eMol26242022	−7.9	−8.3	−7.8	204	eMol579756	−8.0	−8.1	−7.6
142	CHEMBL3817888	−8.1	−8.3	−7.7	205	eMol20361212	−8.1	−8.1	−7.5
143	eMol18542848	−8.3	−8.3	−7.7	206	eMol4446697	−7.7	−8.1	−7.5
144	eMol20347873	−8.2	−8.3	−7.7	207	eMol25784531	−7.1	−8.1	−7.5
145	eMol206926236	−8.2	−8.3	−7.7	208	eMol27202348	−8.1	−8.1	−7.5
146	eMol45622749	−7.8	−8.1	−7.7	209	eMol4518648	−8.1	−8.1	−7.5
147	eMol25810071	−8.3	−8.3	−7.7	210	eMol177199339	−7.9	−8.1	−7.5
148	eMol6263471	−8.2	−8.3	−7.7	211	eMol300994251	−7.9	−8.1	−7.5
149	CHEMBL1214647	−8.1	−8.4	−7.7	212	eMol45585836	−7.7	−8.2	−7.5
150	eMol19076598	−7.3	−8.3	−7.7	213	eMol4577396	−8.0	−8.1	−7.5
151	eMol25810083	−8.3	−8.3	−7.7	214	eMol48768479	−7.9	−8.1	−7.5
152	eMol46490873	−8.1	−8.2	−7.7	215	eMol4566756	−7.9	−8.1	−7.5
153	eMol46365626	−8.2	−8.3	−7.7	216	eMol20378767	−7.9	−8.1	−7.5
154	eMol566574	−8.1	−8.3	−7.7	217	eMol580072	−8.0	−8.1	−7.5
155	CHEMBL3818929	−8.2	−8.3	−7.7	218	eMol179164947	−7.8	−8.0	−7.5
156	CHEMBL518358	−8.2	−8.3	−7.7	219	eMol46367311	−7.8	−8.0	−7.5
157	eMol13856171	−8.0	−8.2	−7.7	220	eMol565798	−8.1	−8.1	−7.5
158	eMol26809774	−8.2	−8.2	−7.7	221	eMol27091442	−7.9	−8.1	−7.5
159	CHEMBL1214935	−8.1	−8.3	−7.7	222	eMol27202916	−7.2	−8.1	−7.5
160	eMol26242038	−7.9	−8.2	−7.7	223	eMol4397966	−8.0	−8.1	−7.5
161	eMol26314331	−7.5	−8.3	−7.7	224	eMol679550	−7.9	−8.1	−7.5
162	eMol43906051	−8.1	−8.3	−7.7	225	CHEMBL1214934	−7.0	−8.0	−7.5
163	CHEMBL1214936	−8.2	−8.3	−7.7	226	eMol25966646	−7.9	−8.0	−7.5
164	eMol26196312	−8.2	−8.3	−7.7	227	eMol27202242	−8.0	−8.0	−7.5
165	eMol27202258	−8.3	−8.2	−7.7	228	eMol28321864	−8.0	−8.0	−7.4
166	eMol45585832	−8.2	−8.3	−7.7	229	eMol4549830	−7.3	−8.0	−7.4
167	eMol681936	−8.2	−8.2	−7.7	230	eMol1035521	−7.9	−8.0	−7.4
168	eMol25819217	−8.3	−8.2	−7.6	231	eMol206992517	−8.0	−8.0	−7.4
169	eMol26320021	−7.4	−8.2	−7.6	232	eMol579718	−8.0	−8.1	−7.4
170	eMol30323345	−9.8	−8.2	−7.6	233	eMol581308	−7.9	−8.0	−7.4
171	eMol43731530	−7.3	−8.2	−7.6	234	CHEMBL485839	−8.0	−8.0	−7.4
172	eMol46480165	−8.2	−8.2	−7.6	235	eMol43906047	−7.9	−8.0	−7.4
173	eMol1088331	−8.2	−8.2	−7.6	236	eMol43906057	−7.4	−8.0	−7.4
174	eMol1755402	−8.0	−8.2	−7.6	237	eMol681814	−7.9	−8.0	−7.4
175	eMol23178762	−8.1	−8.2	−7.6	238	CHEMBL2259943	−7.9	−8.0	−7.4
176	CHEMBL3819481	−7.9	−8.2	−7.6	239	eMol36912834	−8.0	−8.1	−7.4
177	CHEMBL1215578	−8.0	−8.2	−7.6	240	eMol26242052	−7.3	−8.2	−7.4
178	CHEMBL1215797	−8.0	−8.2	−7.6	241	eMol18551342	−8.0	−8.0	−7.4
179	CHEMBL508966	−8.1	−8.2	−7.6	242	eMol4578697	−7.3	−8.1	−7.4
180	eMol20377597	−8.0	−8.2	−7.6	243	eMol4552519	−8.0	−8.1	−7.4
181	eMol24530483	−8.2	−8.2	−7.6	244	CHEMBL3818035	−7.9	−8.4	−7.4
182	eMol29653471	−8.2	−8.2	−7.6	245	eMol679822	−8.0	−8.2	−7.4
183	eMol45622745	−8.2	−8.2	−7.6	246	CHEMBL1214648	−8.0	−8.2	−7.4
184	eMol48982653	−7.7	−8.2	−7.6	247	CHEMBL3582082	−7.3	−8.1	−7.4
185	eMol566488	−8.1	−8.2	−7.6	248	CHEMBL3818775	−8.2	−8.2	−7.4
186	eMol679556	−8.0	−8.2	−7.6	249	eMol11920256	−8.1	−8.2	−7.4
187	eMol956623	−8.2	−8.2	−7.6	250	CHEMBL1534883	−9.0	−9.1	−7.4
188	eMol18550846	−8.1	−8.2	−7.6					

[a] Data ranked based on the Expensive docking score.

Table S4. Estimated conventional, intermediate, and expensive docking scores, MM-GBSA//MM binding energies and binding features of AMBER-based minimized structures for QNZ and the top 35 potent inhibitors against mutated PBP2a allosteric site in acidic medium.[a]

No.	Compound Name/Code	Docking score (kcal/mol)			MM-GBSA//MM binding energy (kcal/mol)	Binding Features[b]
		Conventional	Intermediate	Expensive		
1	QNZ	–6.9	–8.0	–8.3	−31.4	VAL277 (2.28 Å), GLU294 (1.57 Å), LYS316 (2.60, 2.72 Å)
2	eMol26313223	–8.3	–8.4	–10.0	−39.6	LYS146 (1.89 Å), LYS273 (1.94, 2.21 Å), LYS316 (2.19 Å)
3	eMol26314565	–8.2	–8.2	–9.6	−39.1	LYS146 (1.97 Å), LYS273 (2.03 Å), LYS316 (3.08 Å)
4	eMol26437582	–8.2	–8.9	–9.9	−38.2	GLU294 (1.59 Å), LYS316 (1.82 Å)
5	eMol29634797	–7.6	–8.1	–8.6	−37.1	LYS146 (2.14 Å), LYS273 (1.77, 2.46 Å), ASP295 (1.61 Å), LYS316 (1.92 Å)
6	eMol29597031	–9.2	–9.3	–9.3	−35.2	LYS146 (2.21 Å), VAL277 (1.98 Å), GLU294 (1.62 Å), LYS316 (1.80 Å)
7	eMol29633913	–7.8	–8.6	–8.9	−34.2	GLU294 (1.67 Å), LYS316 (1.83 Å)
8	eMol26314779	–8.1	–8.2	–8.3	−34.2	LYS146 (2.10 Å), LYS273 (1.90 Å), LYS316 (2.85 Å)
9	eMol26269394	–8.5	–8.7	–9.1	−31.6	LYS146 (2.01 Å), LYS273 (2.02 Å), GLU294 (1.57 Å), LYS316 (1.79 Å)
10	eMol27252412	–8.1	–8.2	–8.7	−29.8	LYS316 (2.24 Å)
11	eMol300094331	–8.2	–8.3	–8.7	−29.6	GLU294 (1.60 Å), LYS316 (1.99 Å)
12	eMol30017880	–7.4	–8.3	–8.7	−29.3	TYR105 (2.04 Å), LYS146 (2.16 Å), GLU294 (1.58 Å), LYS316 (1.86 Å)
13	eMol26242018	–8.0	–8.8	–9.0	−29.1	LYS146 (2.16 Å), GLU294 (1.58 Å), LYS316 (1.86 Å)
14	eMol27091498	–8.0	–8.1	–8.4	−29.1	LYS273 (1.92 Å), ASP295 (1.97 Å), LYS316 (1.99 Å)
15	eMol26313117	–8.9	–9.4	–9.5	−29.0	LYS146 (2.21 Å), ASP295 (2.01 Å), LYS316 (1.90 Å)
16	eMol27406062	–7.8	–8.3	–8.6	−28.6	LYS146 (2.07 Å), LYS273 (1.99 Å), GLU294 (1.60 Å), ASP295 (2.60 Å), LYS316 (1.85 Å)
17	eMol27091674	–8.0	–8.1	–8.4	−28.4	LYS273 (1.92 Å), ASP295 (1.99 Å), LYS316 (1.97 Å)

Table S4. *Continued.*

No.	Compound Name/Code	Docking score (kcal/mol)			MM-GBSA//MM binding energy (kcal/mol)	Binding Features[b]
		Conventional	Intermediate	Expensive		
18	eMol26242042	–7.6	–8.5	–8.5	−28.2	LYS146 (2.22 Å), LYS273 (2.01 Å), ASP295 (2.13 Å), LYS316 (1.86 Å)
19	eMol26242026	–7.8	–8.2	–8.4	−27.9	LYS146 (2.21 Å), LYS273 (1.99 Å), ASP295 (2.09 Å), LYS316 (1.86 Å)
20	eMol301527162	–8.0	–8.1	–8.3	−27.8	LYS273 (1.89 Å), ASP295 (1.92, 2.83 Å), LYS316 (2.36, 3.02, 3.04, 3.07 Å)
21	eMol26293960	–8.1	–9.2	–9.4	−27.4	LYS146 (2.31 Å), ASP295 (2.12 Å), LYS316 (1.90 Å)
22	eMol3021959	–7.1	–8.9	–9.4	−27.1	LYS316 (1.86 Å)
23	CHEMBL1215004	–7.7	–8.2	–8.4	−26.7	LYS146 (2.24 Å), HIS293 (2.89 Å), GLU294 (1.62 Å), ASP295 (2.04 Å), LYS316 (1.80 Å)
24	CHEMBL1215080	–8.4	–8.4	–8.9	−26.4	LYS146 (1.99 Å), LYS273 (2.01 Å), GLU294 (1.56 Å), LYS316 (1.80 Å)
25	eMol26262168	–7.0	–8.0	–8.5	−26.3	LYS146 (2.22 Å), GLU294 (1.61 Å), LYS316 (1.80 Å)
26	CHEMBL1215082	–7.4	–8.0	–8.5	−25.5	LYS146 (1.97 Å), LYS273 (2.17 Å), GLU294 (1.56 Å), LYS316 (1.81 Å)
27	eMol29565259	–7.8	–8.2	–8.6	−25.1	LYS146 (1.98 Å), LYS273 (1.95, 2.96 Å), GLU294 (1.57 Å), LYS316 (1.81 Å)
28	eMol26330545	–8.2	–8.2	–8.4	−25.1	LYS273 (2.06 Å), LYS316 (1.98 Å)
29	eMol27202248	–8.4	–8.3	–8.6	−24.5	LYS273 (2.15 Å), LYS316 (2.01 Å)
30	eMol27202760	–8.0	–8.1	–9.1	−24.5	LYS273 (1.94 Å), LYS316 (1.92 Å)
31	eMol26264570	–8.1	–8.4	–8.7	−24.3	LYS273 (2.00, 2.02 Å), GLU294 (1.62 Å), LYS316 (1.83 Å)
32	eMol27202948	–7.4	–8.0	–8.3	−24.0	LYS273 (2.30 Å), ASP295 (2.18 Å), LYS316 (2.59 Å)
33	eMol27202252	–8.0	–8.1	–8.6	−23.3	LYS316 (1.89 Å)
34	eMol299980544	–8.2	–8.2	–8.6	−23.2	LYS316 (1.96 Å)
35	eMol26319231	–8.2	–8.6	–8.7	−22.9	LYS273 (2.01 Å), LYS316 (1.95 Å)
36	eMol300154219	–7.3	–8.3	–8.5	−21.0	LYS273 (1.92, 2.45 Å), ASP295 (2.21 Å), LYS316 (2.51 Å)

[a] Data ranked based on the MM-GBSA//MM binding energy. [b] Only hydrogen bonds (in Å) were displayed.
